# Supplementary material for: Accuracy of potential diagnostic indicators for coeliac disease: a systematic review protocol
Source: BMJ Open. 2020 Oct 5;10(10):e038994. doi: 10.1136/bmjopen-2020-038994 (PMC7537462; doi:10.1136/bmjopen-2020-038994)
Supplement: Supplementary data [file bmjopen-2020-038994supp001.pdf]

Supplementary materials

Search strategy

Database: Embase <1974 to 2020 Week 05>

Search Strategy: *ARC-Coeliac-Embase-Final*

----

1 Celiac Disease/ (29729)

2 c?eliac.ti,kw. (22813)

3 c?eliac.af. and gastroenterology.ec. (22352)

4 (celiac or coeliac).ab. /freq=3 (9328)

5 or/1-4 (39462)

6 ((detect* or diagnos* or identif* or decision* or predict* or prognos* or risk or risks or stratif* or validat*) adj3 (model* or factor* or algorithm? or scor* or system or technique? or aid? or rule or rules or index or variable* or tool? or panel or criteri* or characteristic? or history or finding* or value* or assay or stratif* or biomarker?)).ti,ab,kw. (2855495)

7 ((detect* or diagnos* or identif* or decision* or predict* or prognos* or stratif* or validat*) adj3 (risk or risks or model* or factor* or algorithm? or scor* or system or technique? or aid? or rule or rules or index or variable* or tool? or panel or criteri* or characteristic? or history or finding* or value* or assay or stratif* or biomarker?)).ti,ab,kw. (2225766)

8 (clinic* adj3 (model* or algorithm? or scor* or risk or risks or rule or rules or predict* or index or tool? or panel or criteri* or decision* or stratif* or diagnos* or detect* or identif* or probabilit*)).ti,ab,kw. (632961)

9 ((multivaria* or multicomponent?) adj3 (model* or algorithm? or scor* or rule or rules or index or tool? or panel or criteri*)).ti,ab,kw. (110662)

10 clinical decision making/ or statistical model/ or algorithm/ (444594)

11 or/6-10 (3678637)

12 5 and 11 (6036)

13 *physical disease by body function/ or exp *abnormal blood pressure/ or exp *abnormal posture/ or *aerophagia/ or exp *appetite disorder/ or *asthenia/ or *asymptomatic disease/ or exp *autonomic dysfunction/ or exp *balance disorder/ or exp *blood clotting disorder/ or exp *body temperature disorder/ or exp *body weight disorder/ or exp *common cold symptom/ or exp *consciousness disorder/ or exp *constipation/ or exp *coughing/ or exp *cyanosis/ or exp *diarrhea/ or exp *disability/ or exp *"disorders of higher cerebral function"/ or exp *dysphagia/ or *eructation/ or exp *faintness/ or exp *fatigue/ or *functional disease/ or exp *growth disorder/ or exp *immunopathology/ or exp *incontinence/ or exp *infertility/ or *listlessness/ or *malaise/ or *meningism/ or exp *metabolic disorder/ or exp *micturition disorder/ or exp *motor dysfunction/ or exp *multiple organ failure/ or exp *"nausea and vomiting"/ or exp *nutritional disorder/ or exp

*pain/ or *pallor/ or exp *pregnancy disorder/ or *qi deficiency/ or *qi stagnation/ or exp *reflex disorder/ or exp *respiratory function disorder/ or exp *salivation disorder/ or exp *sensory dysfunction/ or exp *sexual dysfunction/ or exp *shock/ or exp *sleep disorder/ or exp *speech disorder/ or *weakness/ or *yang deficiency/ or *yin deficiency/ (4991033)

14 *physical disease by anatomical structure/ or exp *abdominal disease/ or exp *abnormal body build/ or exp *breast disease/ or exp *cardiovascular disease/ or exp *connective tissue disease/ or exp *digestive system disease/ or exp *ear nose throat disease/ or exp *endocrine disease/ or exp *eye disease/ or exp *"head and neck disease"/ or exp *hematologic disease/ or exp *mouth disease/ or exp *mucosal disease/ or exp *musculoskeletal disease/ or exp *neurologic disease/ or exp *pelvic disease/ or exp *respiratory tract disease/ or exp *skin disease/ or exp *soft tissue disease/ or exp *thorax disease/ or exp *urogenital tract disease/ (11959905)

15 *"physical disease by composition of body fluids, excreta and secretions"/ or exp *abnormal feces composition/ or exp *abnormal substrate concentration in blood/ or exp *abnormal urine composition/ or *dehydration/ or *hypervolemia/ (344197)

16 ((addison* adj3 (disease* or disorder* or syndrom*)) or alopecia or baldness or amenorrhea* or amenorrhoea* or oligomenorrhea* or oligomenorrhoea* or anxiety disorder* or anorexia or adrenal insufficienc* or adrenocortical insufficienc* or allerg* or anemi* or anaemi* or angina or aneurysm or ankylosing spondylitis or arthropath* or arthriti* or arthrosis or arthroses or asthma* or asthenia or ataxia or atrial fibrillation or atrophy or autoimmune disorder* or (autoimmune adj (disease* or disorder* or syndrom*)) or ataxia or avitaminosis or bonnevie ullrich or back pain or (biliary adj3 (disease* or disorder* or syndrom*)) or (bipolar adj3 disorder*) or blindness or blind loop syndrom* or (blood adj3 poisoning) or brain atroph* or ((bone or bones) adj3 (broken or disease* or disorder* or syndrom*)) or (bone* adj3 (mineral* or densit* or soft* or decay*)) or (brain adj3 (disease* or disorder* or syndrom*)) or (bronchi* adj3 (disease* or disorder* or syndrom*)) or (bowel* adj3 (disease* or disorder* or syndrom*)) or calcinosis or cancer* or carcinoma* or adenocarcinoma* or neoplasm* or neoplastic or metasta* or malignan* or tumour or tumours or tumor or tumors or (cardiac adj3 (arrest or arrhythmia* or disease* or disorder* or surg* or syndrom*)) or cardiomyopath* or ((cardiovascular or coronary) adj3 (disease* or disorder* or event* or syndrom*)) or cachexia or ((cecal or colon* or duoden*) adj3 (disease* or disorder* or syndrom*)) or cerebral palsy or (cerebro* adj3 (degenerat* or disease* or disorder* or event* or syndrom*)) or chronic obstructive disease* or cirrhosis or claudication or colic or copd or ((coordination or co-ordination) adj3 (impair* or lack)) or congenital abnormalit* or (congential adj3 (disease* or disorder* or syndrom*)) or ((connective or collagen* or skin) adj3 (disease* or disorder* or syndrom*)) or collagenous sprue or colitis or colotides or coxarthrosis or crohn* or cushing* or cyanosis or cystic fibrosis or cystitis or deaf* or deformit* or delayed puberty or dental or depressive disorder* or dysphoria or dysthymia or disabled or (physical adj3 (deform* or disab* or impair*)) or dermatitis or dermato* or dorsopath* or diabet* or down* syndrom* or duhring* or duehring* or duhrig* or duehrig* or dyscoordination or dys-coordination or dys-coordination or dys-co-ordination or dysentery or dyssynergia or dys-synergia or emaciat* or enteritis or enterocolitis* or enteropathy or dystonia or eczema or edema or oedema or elfin face sydrom* or encephalopath* or (endocrine adj3 (disease* or disorder* or syndrom*)) or enuresis or enteropath* or epilep* or (eye adj3 (disease* or disorder* or manifestation* or syndrom*)) or fatigue syndrome or chronic fatigue or failure to thrive or fibromyalgia or fibrosis or food hypersensitivity or fractures or gammaglobulinemia or gammaglobulinaemia or (gardner* adj3 (syndrom* or disease* or disorder*)) or gastritis or gastroenteritis or gout or (glomerul* adj3 (disease* or disorder* or syndrom*)) or (gonodal adj3 dysgen*) or (growth adj3 (disease* or disorder* or syndrom*)) or headache* or

((hemic or haemic or lymph*) adj3 (disease* or disorder* or syndrom*)) or hematuria or haematuria or hemophili* or haemophili* or ((hearing or visual or vision or sight) adj3 (aid* or impair* or loss)) or (heart adj3 (disease* or disorder* or syndrom* or manifestation* or myopath*)) or hemiplegi* or hepatitis or hemodialysis or haemodialysis or (heart adj3 (disease* or disorder* or failure or syndrom*)) or hiv or human immunodeficiency virus or acquired immunodeficieny syndrom* or heerfordt waldenstrom* or hidroa or hydroa or hippel lindau* or hypertensi* or hypotensi* or hyperthyroidism or hypothyroidism or hypocortisolism or hypocorticism or hypoadrenalism or hyperthyroidism or hypothyroidism or incoordination or inco-ordination or in-coordination or in-co-ordination or infertility or subfertility or sub-fertility or sterility or inflammatory disease* or inflammatory bowel disease* or incontinen* or intestinal atresi* or intussusception or irritable bowel or ischemi* or ischaemi* or fistula* or (joint adj3 (disease* or disorder* or syndrom*)) or (jejunal adj3 (disease* or disorder* or syndrom*)) or kyphosis or lav-htlv* or leukemia or leukaemia or ((liver or hepatic) adj3 (disease* or disorder* or failure or syndrom*)) or lordosis or (lung adj3 (disease* or disorder*)) or lupus or lymphoma or lymphogranuloma* or lymphadenopath* or lymphotrop* or machado joseph* or macular degeneration or malnutrition or (mental adj2 (disorder* or disease* or health or illness*)) or mania or manic or (menstr* adj3 (ceas* or disturb* or disease* or disorder* or stop* or syndrom*)) or migraine* or (mitochondrial adj3 (disease* or disorder* or syndrom*)) or movement disorder* or mucinosis or musculoskeletal or narp syndrom* or necrotizing or nephrotic* or neuromuscular or non-hodgkin* or nonhodgkin* or multiple sclerosis or myeloma or myocarditis or myocardiopath* or myopath* or (myocardi* adj3 (deteri* or disease* or disorder* or syndrom* or manifestation*)) or nephrotic syndrome* or ((neurodegenerat* or neuro-degenerat) adj3 (disease* or disorder* or syndrom*)) or ((nutritional or metabolic) adj3 (disease* or disorder* or syndrom*)) or ((organ* or kidney or stem cell) adj3 (transplant* or recipient*)) or (nervous system adj3 (disease* or disorder* or syndrom*)) or (neurological adj3 (condition* or disease* or disorder* or syndrom*)) or neuropath* or neurosarcoido* or occlusion* or obesity or obese or orthopedic* or orthopaedic* or ((esophageal or oesophageal) adj3 (disease* or disorder* or syndrom*)) or ((oral or mouth) adj3 (disease* or disorder* or syndrom* or manifestation*)) or osteo* or otitis media or otorhinolaryngolog* or otosclerosis or (pancrea* adj3 (disease* or disorder* or syndrom*)) or papulosquamous or paraplegi* or parkinson* or (peripheral adj3 (arterial or vascular or disease* or disorder* or syndrom*)) or (peritoneal adj3 (disease* or disorder* or syndrom*)) or photodermato* or pick disease* or pneumo* or polio* or polyp* or polydipsia or polyarthropath* or polyarteritis or polyarthrosis or polyneuropath* or porphyrias or (pregnancy adj3 complicat*) or premature aging or proteostasis or pseudophakia or psoriasis or parapsoriasis or prolapse or (pulmonary adj3 (disease* or disorder* or syndrom*)) or purpur* or (recurren* adj3 (abortion* or miscarr*)) or (rect* adj3 (disease* or disorder* or syndrom*)) or ((renal or kidney) adj3 (disease* or disorder* or failure or syndrom*)) or (respiratory adj3 (disease* or disorder* or syndrom*)) or reticulocytosis or retinopathy or rheumat* or ricket* or sarcoido* or sepsis or septic* or seizure* or sclerosis or scoliosis or sickle cell or ((sjogren* or sjoegren* or sicca*) adj3 (syndrom* or disease* or disorder*)) or ((skin or connective tissue) adj3 (disease* or disorder* or syndrom*)) or sleep disorder* or sleep apnea or sleep apnoea or insomnia* or dyssomnia* or hypersomnia* or spina bifida or muscular atropy or short stature* or short bowel syndrom* or (spin* adj3 (degenerat* or disease* or disorder* or syndrom*)) or (spleen* adj3 (disease* or disorder* or syndrom*)) or spondylo* or stenosis* or stoma* or (stomach adj3 (disease* or disorder* or syndrom*)) or stroke or strokes or cerebral infarct* or tetraplegi* or thyroiditis* or (thyroid* adj3 (disease* or disorder* or dysfunction* or syndrom*)) or ((tooth or teeth or enamel) adj3 (decay* or discolo* or disease* or disorder* or dysfunction* or syndrom*)) or tropical sprue or tuberculosis or (systemic adj3 (disorder* or disease* or syndrom*)) or thrombocyto* or tremor or tremors or (turner* adj3 (syndrom* or disease* or disorder*)) or ulcer* or (urogenital adj3 (disease*

or disorder* or syndrom*)) or vasculopath* or (vascular adj3 (disease* or disorder* or occlu* or syndrom*)) or vestibular or ((virus or viral or bacteri* or parasit*) adj3 (infection* or disease*)) or (wasting adj3 (disorder* or disease* or syndrom*)) or (whipple adj3 (disorder* or disease* or syndrom*)) or (william* adj3 (syndrom* or disease* or disorder*)) or zoster*).ti,ab,kw. (14232139)

17 ((digestive system* or duoden* or gastr* or intestin* or nutrition* or malabsor* or metabolic*) adj3 (disease* or disorder* or syndrom* or manifestation*)).ti,ab,kw. (254303)

18 (constipation or constipated or diarrhoea or diarrhea or (abdominal adj3 (disten* or pain or bloating or cramp or cramps)) or flatulence or meteorism or steatorrhoea or steatorrhea or ((acid base or calcium or cyanocobalamin* or electrolyte* or folate or folic acid or glucose or IgA or immunoglobulin A or iron or lactose or lipid* or phosph* or protein* or triglyceride* or vitamin* or mineral*) adj3 (disorder* or deficien* or imbalance* or insufficiency or intoleran*)) or fever* or febrile or malaise or fatigue or letharg* or exhaustion or vomiting or nausea or emesis or sickness or weight loss or wait gain or hot flashes or hot flushes or medically unexplained or unexplained symptoms or unexplained medical or (signs adj2 symptoms) or sleepiness or ((calciferol or cholecalciferol or colecalciferol or egocalciferol) adj3 (disorder* or deficien* or imbalance* or insufficiency or intoleran*)) or conditions linked or linked conditions).ti,ab,kw. (1126673)

19 (medical* morbid* or (medical* adj3 comorbid*) or (medical* adj3 co-morbid*) or multimorbid* or multi* morbid* or multi* comorbid* or multi* co-morbid* or multi* physical*).ti,ab,kw. (30923)

20 first-degree relative/ (6249)

21 ((first adj5 (relative* or relation?)) and c?eliac).ti,ab,kw. (452)

22 or/13-21 (18446309)

23 (((case? adj2 find*) or detect* or diagnos* or predict* or incidence or prevalence or seroprevalence* or risk or risk factor? or risk indicator? or screen* or test*) adj3 c?eliac*).ti,ab,kw. (7675)

24 (c?eliac and ((detect* or diagnos* or predict* or incidence or prevalence or seroprevalence* or risk or risk factor? or screen* or test*) adj2 CD)).ab. (3440)

25 (((association? or associated or indicator? or indicated or predict*) adj3 (risk? or symptom* or disease? or disorder? or histor*)) and c?eliac).ti,ab,kw. (4503)

26 ((association between or relationship between) and c?eliac?).ti,ab,kw. (2279)

27 *celiac disease/di [Diagnosis] (5931)

28 celiac disease/ and (*diagnosis/ or *differential diagnosis/ or *seroprevalence/ or *screening/ or *screening test/ or *incidence/ or *prevalence/) (1064)

29 celiac disease/ and ("prediction and forecasting"/ or prediction/ or predictive validity/ or predictive value/) (755)

30 or/23-29 (16111)

31 5 and 22 and 30 (15090)

32 (((double* or dual*) adj5 diagnos* adj5 c?eliac?) or (comorbid* adj5 c?eliac)).ti,ab,kw. (85)

33 (high risk adj5 c?eliac?).ti,ab,kw. (103)

34 (famil* histor* adj2 c?eliac).ti,ab,kw. (93)

35 (children adj of* adj5 c?eliac).ti,ab,kw. (30)

36 celiac disease test kit/ (11)

37 or/32-36 (315)

38 12 or 31 or 37 (17603)

39 limit 38 to embase status (11243)

40 ((celiac* or coeliac*) adj2 (asymptomatic or atypical or adolesc* or adult* or cases or cohort* or child* or disease* or family or families or familial or genetic predispos* or genetic pre-dispos* or heredit* or infant* or men or patient* or people or population* or subjects or symptomatic or syndrom* or sprue or women)).ti,ab,kw. (26735)

41 limit 40 to (article-in-press status or in-process status) (372)

42 c?eliac.ti. (20558)

43 limit 38 to conference abstract status (4569)

44 42 and 43 (2794)

45 39 or 41 or 44 (14409)

46 ((celiac* or coeliac*) adj (angiograp* or arter* or axis or plexus or trunk)).ti,ab,kw,hw. (12186)

47 case report.ti. (283693)

48 ((case adj of*) and (patient or man or woman)).ti,ab. and case report.sh. (347041)

49 (editorial or letter).pt. (1747770)

50 (exp experimental organism/ or animal tissue/ or animal cell/ or exp animal disease/ or exp carnivore disease/ or exp bird/ or exp experimental animal welfare/ or exp animal husbandry/ or animal behavior/ or exp animal cell culture/ or exp marine species/ or nonhuman/ or animal.hw.) not human/ (6072159)

51 ((rat or rats or mouse or mice or rodent* or animal* or murine or porcine or feline or canine or dog or dogs or cat or cats or pig or pigs or monkey* or macaque*) not human*).ti. (1957387)

52 or/46-51 (8620748)

53 45 not 52 (12083)

54 case control study/ or hospital based case control study/ or population based case control study/ or exp longitudinal study/ or prospective study/ or retrospective study/ (1667885)

55 case finding/ or cohort analysis/ or control group/ or correlational study/ or cross-sectional study/ (1006525)

56 seroepidemiology/ (3934)

57 (cohort or case control* or case finding or cross-sectional or longitudinal).ti,ab,kw. (1661606)

58 study.ti. (1569542)

59 (study and (analys* or cases or clinical or control* or compar* or correlat* or associat* or epidemiolog* or evaluat* or examin* or investigat* or observ* or population based or prospectiv* or retrospectiv* or serolog*)).ti,ab,kw. (8983424)

60 (case? and control*).ab. (657067)

61 (controls or (control adj (group? or participants or patients))).ab. (1656326)

62 (groups or subgroup? or sub-group?).ab. (3028674)

63 (group? adj5 (analys* or compar* or control* or correlat* or associat*)).ab. (1489755)

64 exp regression analysis/ (434324)

65 statistical model/ (158724)

66 ((analys* or logistic*) adj1 (model* or regression*)).ab. (661228)

67 study.ab. /freq=2 (3165201)

68 or/54-67 (12212707)

69 53 and 68 (7566)

70 limit 69 to yr="1997 -Current" (7032)

****

Database: Ovid MEDLINE(R) and Epub Ahead of Print, In-Process & Other Non-Indexed Citations and Daily <1946 to February 05, 2020>

Search Strategy: ARC-Coeliac-MEDLINE-Final

----

1 Celiac Disease/ (19693)

2 c?eliac.ti,ab,kf. (27278)

3 or/1-2 (32722)

4 ((detect* or diagnos* or identif* or decision* or predict* or prognos* or risk or risks or stratif* or validat*) adj3 (model* or factor* or algorithm? or scor* or system or technique? or aid? or rule or rules or index or variable* or tool? or panel or criteri* or characteristic? or history or finding* or value* or assay or stratif* or biomarker?)).ti,ab,kf. (1978188)

5 ((detect* or diagnos* or identif* or decision* or predict* or prognos* or stratif* or validat*) adj3 (risk or risks or model* or factor* or algorithm? or scor* or system or technique? or aid? or rule or rules or index or variable* or tool? or panel or criteri* or characteristic? or history or finding* or value* or assay or stratif* or biomarker?)).ti,ab,kf. (1547617)

6 (clinic* adj3 (model* or algorithm? or scor* or risk or risks or rule or rules or predict* or index or tool? or panel or criteri* or decision* or stratif* or diagnos* or detect* or identif* or probabilit*)).ti,ab,kf. (420165)

7 ((multivaria* or multicomponent?) adj3 (model* or algorithm? or scor* or rule or rules or index or tool? or panel or criteri*)).ti,ab,kf. (69716)

8 (clinical decision making or (decision and logistic models)).sh. (7874)

9 or/4-8 (2337727)

10 3 and 9 (3401)

11 Risk Factors/ or Disease Predisposition/ or Genetic Predisposition to Disease/ (907433)

12 exp "bacterial infections and mycoses"/ or exp virus diseases/ or exp parasitic diseases/ or exp neoplasms/ or exp musculoskeletal diseases/ or exp stomatognathic diseases/ or exp respiratory tract diseases/ or exp otorhinolaryngologic diseases/ or exp nervous system diseases/ or exp eye diseases/ or exp male urogenital diseases/ or exp "female urogenital diseases and pregnancy complications"/ or exp cardiovascular diseases/ or exp "hemic and lymphatic diseases"/ or exp "congenital, hereditary, and neonatal diseases and abnormalities"/ or exp "skin and connective tissue diseases"/ or exp endocrine system diseases/ or exp immune system diseases/ or exp "disorders of environmental origin"/ or disease/ or digestive system diseases/ or exp biliary tract diseases/ or exp digestive system abnormalities/ or exp digestive system fistula/ or exp digestive system neoplasms/ or exp liver diseases/ or exp pancreatic diseases/ or exp peritoneal diseases/ or gastrointestinal diseases/ or exp esophageal diseases/ or exp gastroenteritis/ or exp gastrointestinal hemorrhage/ or exp gastrointestinal neoplasms/ or intestinal diseases/ or exp cecal diseases/ or exp colonic diseases/ or exp duodenal diseases/ or exp dysentery/ or exp enteritis/ or exp enterocolitis/ or exp hiv enteropathy/ or exp ileal diseases/ or exp inflammatory bowel diseases/ or exp intestinal atresia/ or exp intestinal diseases, parasitic/ or exp intestinal fistula/ or exp intestinal neoplasms/ or exp intestinal obstruction/ or exp intestinal perforation/ or exp intestinal polyposis/ or exp jejunal diseases/ or malabsorption syndromes/ or blind loop syndrome/ or collagenous sprue/ or lactose intolerance/ or short bowel syndrome/ or sprue, tropical/ or steatorrhea/ or whipple disease/ or mesenteric ischemia/ or mesenteric vascular occlusion/ or pneumatosis cystoides intestinalis/ or protein-losing enteropathies/ or exp rectal diseases/ or zollinger-ellison syndrome/ or exp stomach diseases/ or exp tuberculosis, gastrointestinal/ or exp visceral prolapse/ or exp liver diseases/ or exp pancreatic diseases/ or exp peritoneal diseases/ or "nutritional and metabolic diseases"/ or metabolic diseases/ or exp acid-base imbalance/ or exp bone diseases, metabolic/ or exp brain diseases, metabolic/ or exp calcium metabolism disorders/ or exp dna repair-deficiency disorders/ or exp glucose metabolism disorders/ or exp hyperlactatemia/ or exp iron metabolism disorders/ or exp lipid metabolism disorders/ or malabsorption syndromes/ or exp blind loop syndrome/ or exp collagenous sprue/ or exp hyperhomocysteinemia/ or exp lactose intolerance/ or exp sprue, tropical/ or exp steatorrhea/ or exp whipple disease/ or exp metabolic syndrome/ or exp metabolism, inborn errors/ or exp mitochondrial diseases/ or exp phosphorus metabolism disorders/ or exp porphyrias/ or exp proteostasis deficiencies/ or exp skin diseases, metabolic/ or exp wasting syndrome/ or exp water-electrolyte imbalance/ or exp nutrition disorders/ or "SIGNS and SYMPTOMS"/ or exp aging, premature/ or exp asthenia/ or exp body temperature changes/ or exp body weight/ or exp cardiac output, high/ or exp cardiac output, low/ or exp chills/ or exp cyanosis/ or exp edema/ or exp eye manifestations/ or exp failure to thrive/ or exp fatigue/ or exp feminization/ or exp fetal distress/ or exp heart murmurs/ or exp hot flashes/ or exp

hypergammaglobulinemia/ or exp hyperlactatemia/ or exp hypertriglyceridemic waist/ or exp intermittent claudication/ or exp medically unexplained symptoms/ or exp mobility limitation/ or exp motion sickness/ or exp myocardial stunning/ or exp neurologic manifestations/ or exp oral manifestations/ or exp polydipsia/ or exp prodromal symptoms/ or exp pseudophakia/ or exp renal colic/ or exp reticulocytosis/ or exp "signs and symptoms, digestive"/ or exp "signs and symptoms, respiratory"/ or exp skin manifestations/ or exp sleepiness/ or exp travel-related illness/ or exp urological manifestations/ or exp virilism/ (13521118)

13 exp "anatomy (non mesh)"/ab, pa [abnormalities, pathology] (1686610)

14 ((addison* adj3 (disease* or disorder* or syndrom*)) or alopecia or baldness or amenorrhea* or amenorrhoea* or oligomenorrhea* or oligomenorrhoea* or anxiety disorder* or anorexia or adrenal insufficienc* or adrenocortical insufficienc* or allerg* or anemi* or anaemi* or angina or aneurysm or ankylosing spondylitis or arthropath* or arthriti* or arthrosis or arthroses or asthma* or asthenia or ataxia or atrial fibrillation or atrophy or autoimmune disorder* or (autoimmune adj (disease* or disorder* or syndrom*)) or ataxia or avitaminosis or bonnevie ullrich or back pain or (biliary adj3 (disease* or disorder* or syndrom*)) or (bipolar adj3 disorder*) or blindness or blind loop syndrom* or (blood adj3 poisoning) or brain atroph* or ((bone or bones) adj3 (broken or disease* or disorder* or syndrom*)) or (bone* adj3 (mineral* or densit* or soft* or decay*)) or (brain adj3 (disease* or disorder* or syndrom*)) or (bronchi* adj3 (disease* or disorder* or syndrom*)) or (bowel* adj3 (disease* or disorder* or syndrom*)) or calcinosis or cancer* or carcinoma* or adenocarcinoma* or neoplasm* or neoplastic or metasta* or malignan* or tumour or tumours or tumor or tumors or (cardiac adj3 (arrest or arrhythmia* or disease* or disorder* or surg* or syndrom*)) or cardiomyopath* or ((cardiovascular or coronary) adj3 (disease* or disorder* or event* or syndrom*)) or cachexia or ((cecal or colon* or duoden*) adj3 (disease* or disorder* or syndrom*)) or cerebral palsy or (cerebro* adj3 (degenerat* or disease* or disorder* or event* or syndrom*)) or chronic obstructive disease* or cirrhosis or claudication or colic or copd or ((coordination or co-ordination) adj3 (impair* or lack)) or congenital abnormalit* or (congential adj3 (disease* or disorder* or syndrom*)) or ((connective or collagen* or skin) adj3 (disease* or disorder* or syndrom*)) or collagenous sprue or colitis or colotides or coxarthrosis or crohn* or cushing* or cyanosis or cystic fibrosis or cystitis or deaf* or deformit* or delayed puberty or dental or depressive disorder* or dysphoria or dysthymia or disabled or (physical adj3 (deform* or disab* or impair*)) or dermatitis or dermato* or dorsopath* or diabet* or down* syndrom* or duhring* or duehring* or duhrig* or duehrig* or dyscoordination or dys-coordination or dys-coordination or dys-co-ordination or dysentery or dyssynergia or dys-synergia or emaciat* or enteritis or enterocolitis* or enteropathy or dystonia or eczema or edema or oedema or elfin face sydrom* or encephalopath* or (endocrine adj3 (disease* or disorder* or syndrom*)) or enuresis or enteropath* or epilep* or (eye adj3 (disease* or disorder* or manifestation* or syndrom*)) or fatigue syndrome or chronic fatigue or failure to thrive or fibromyalgia or fibrosis or food hypersensitivity or fractures or gammaglobulinemia or gammaglobulinaemia or (gardner* adj3 (syndrom* or disease* or disorder*)) or gastritis or gastroenteritis or gout or (glomerul* adj3 (disease* or disorder* or syndrom*)) or (gonodal adj3 dysgen*) or (growth adj3 (disease* or disorder* or syndrom*)) or headache* or ((hemic or haemic or lymph*) adj3 (disease* or disorder* or syndrom*)) or hematuria or haematuria or hemophili* or haemophili* or ((hearing or visual or vision or sight) adj3 (aid* or impair* or loss)) or (heart adj3 (disease* or disorder* or syndrom* or manifestation* or myopath*)) or hemiplegi* or hepatitis or hemodialysis or haemodialysis or (heart adj3 (disease* or disorder* or failure or syndrom*)) or hiv or human immunodeficiency virus or acquired immunodeficieny syndrom* or heerfordt waldenstrom* or hidroa or hydroa or hippel lindau* or hypertensi* or hypotensi* or hyperthyroidism or hypothyroidism or hypocortisolism or hypocorticism or hypoadrenalism or

hyperthyroidism or hypothyroidism or incoordination or inco-ordination or in-coordination or in-co-ordination or infertility or subfertility or sub-fertility or sterility or inflammatory disease* or inflammatory bowel disease* or incontinen* or intestinal atresi* or intussusception or irritable bowel or ischemi* or ischaemi* or fistula* or (joint adj3 (disease* or disorder* or syndrom*)) or (jejunal adj3 (disease* or disorder* or syndrom*)) or kyphosis or lav-htlv* or leukemia or leukaemia or ((liver or hepatic) adj3 (disease* or disorder* or failure or syndrom*)) or lordosis or (lung adj3 (disease* or disorder*)) or lupus or lymphoma or lymphogranuloma* or lymphadenopath* or lymphotrop* or machado joseph* or macular degeneration or malnutrition or (mental adj2 (disorder* or disease* or health or illness*)) or mania or manic or (menstr* adj3 (ceas* or disturb* or disease* or disorder* or stop* or syndrom*)) or migraine* or (mitochondrial adj3 (disease* or disorder* or syndrom*)) or movement disorder* or mucinosis or musculoskeletal or narp syndrom* or necrotizing or nephrotic* or neuromuscular or non-hodgkin* or nonhodgkin* or multiple sclerosis or myeloma or myocarditis or myocardiopath* or myopath* or (myocardi* adj3 (deteri* or disease* or disorder* or syndrom* or manifestation*)) or nephrotic syndrome* or ((neurodegenerat* or neuro-degenerat) adj3 (disease* or disorder* or syndrom*)) or ((nutritional or metabolic) adj3 (disease* or disorder* or syndrom*)) or ((organ* or kidney or stem cell) adj3 (transplant* or recipient*)) or (nervous system adj3 (disease* or disorder* or syndrom*)) or (neurological adj3 (condition* or disease* or disorder* or syndrom*)) or neuropath* or neurosarcoido* or occlusion* or obesity or obese or orthopedic* or orthopaedic* or ((esophageal or oesophageal) adj3 (disease* or disorder* or syndrom*)) or ((oral or mouth) adj3 (disease* or disorder* or syndrom* or manifestation*)) or osteo* or otitis media or otorhinolaryngolog* or otosclerosis or (pancrea* adj3 (disease* or disorder* or syndrom*)) or papulosquamous or paraplegi* or parkinson* or (peripheral adj3 (arterial or vascular or disease* or disorder* or syndrom*)) or (peritoneal adj3 (disease* or disorder* or syndrom*)) or photodermato* or pick disease* or pneumo* or polio* or polyp* or polydipsia or polyarthropath* or polyarteritis or polyarthrosis or polyneuropath* or porphyrias or (pregnancy adj3 complicat*) or premature aging or proteostasis or pseudophakia or psoriasis or parapsoriasis or prolapse or (pulmonary adj3 (disease* or disorder* or syndrom*)) or purpur* or (recurren* adj3 (abortion* or miscarr*)) or (rect* adj3 (disease* or disorder* or syndrom*)) or ((renal or kidney) adj3 (disease* or disorder* or failure or syndrom*)) or (respiratory adj3 (disease* or disorder* or syndrom*)) or reticulocytosis or retinopathy or rheumat* or ricket* or sarcoido* or sepsis or septic* or seizure* or sclerosis or scoliosis or sickle cell or ((sjogren* or sjoegren* or sicca*) adj3 (syndrom* or disease* or disorder*)) or ((skin or connective tissue) adj3 (disease* or disorder* or syndrom*)) or sleep disorder* or sleep apnea or sleep apnoea or insomnia* or dyssomnia* or hypersomnia* or spina bifida or muscular atropy or short stature* or short bowel syndrom* or (spin* adj3 (degenerat* or disease* or disorder* or syndrom*)) or (spleen* adj3 (disease* or disorder* or syndrom*)) or spondylo* or stenosis* or stoma* or (stomach adj3 (disease* or disorder* or syndrom*)) or stroke or strokes or cerebral infarct* or tetraplegi* or thyroiditis* or (thyroid* adj3 (disease* or disorder* or dysfunction* or syndrom*)) or ((tooth or teeth or enamel) adj3 (decay* or discolo* or disease* or disorder* or dysfunction* or syndrom*)) or tropical sprue or tuberculosis or (systemic adj3 (disorder* or disease* or syndrom*)) or thrombocyto* or tremor or tremors or (turner* adj3 (syndrom* or disease* or disorder*)) or ulcer* or (urogenital adj3 (disease* or disorder* or syndrom*)) or vasculopath* or (vascular adj3 (disease* or disorder* or occlu* or syndrom*)) or vestibular or ((virus or viral or bacteri* or parasit*) adj3 (infection* or disease*)) or (wasting adj3 (disorder* or disease* or syndrom*)) or (whipple adj3 (disorder* or disease* or syndrom*)) or (william* adj3 (syndrom* or disease* or disorder*)) or zoster*).ti,ab,kf. (11316008)

15 ((digestive system* or duoden* or gastr* or intestin* or nutrition* or malabsor* or metabolic*) adj3 (disease* or disorder* or syndrom* or manifestation*)).ti,ab,kf. (180683)

16 (constipation or constipated or diarrhoea or diarrhea or (abdominal adj3 (disten* or pain or bloating or cramp or cramps)) or flatulence or meteorism or steatorrhoea or steatorrhea or ((acid base or calcium or cyanocobalamin* or electrolyte* or folate or folic acid or glucose or IgA or immunoglobulin A or iron or lactose or lipid* or phosph* or protein* or triglyceride* or vitamin* or mineral*) adj3 (disorder* or deficien* or imbalance* or insufficiency or intoleran*)) or fever* or febrile or malaise or fatigue or letharg* or exhaustion or vomiting or nausea or emesis or sickness or weight loss or wait gain or hot flashes or hot flushes or medically unexplained or unexplained symptoms or unexplained medical or (signs adj2 symptoms) or sleepiness or ((calciferol or cholecalciferol or colecalciferol or egocalciferol) adj3 (disorder* or deficien* or imbalance* or insufficiency or intoleran*)) or conditions linked or linked conditions).ti,ab,kf. (805001)

17 (medical* morbid* or (medical* adj3 comorbid*) or (medical* adj3 co-morbid*) or multimorbid* or multi* morbid* or multi* comorbid* or multi* co-morbid* or multi* physical*).ti,ab,kf. (18919)

18 (first-degree relative or family or family relation or family history or mother or father or parent or daughter or son).sh. or (brother or sister or sibling).hw. (83159)

19 (first adj5 (relative* or relation?)).ti,ab,kf. (21485)

20 (children adj of*).ti,ab,kf. (32315)

21 or/11-20 (16944516)

22 (((case? adj2 find*) or detect* or diagnos* or predict* or incidence or prevalence or seroprevalence* or risk or risk factor? or risk indicator? or screen* or test*) adj3 c?eliac*).ti,ab,kf. (4839)

23 (c?eliac and ((detect* or diagnos* or predict* or incidence or prevalence or seroprevalence* or risk or risk factor? or screen* or test*) adj2 CD)).ab. (1782)

24 (((association? or associated or indicator? or indicated or predict*) adj3 (risk? or symptom* or disease? or disorder? or histor*)) and c?eliac).ti,ab,kf. (2819)

25 ((association between or relationship between) and c?eliac?).ti,ab,kf. (1328)

26 celiac disease/di [Diagnosis] (5652)

27 celiac disease/ and (diagnosis/ or diagnosis, differential/ or seroprevalence/ or seroepidemiologic studies/ or screening/ or prediction/ or prevalence/ or incidence/) (2995)

28 or/22-27 (11534)

29 3 and 21 and 28 (9724)

30 Celiac Disease/di and (atypical or extraintestinal or extra-intestinal or nonspecific or non-specific).mp. (490)

31 (((double* or dual*) adj5 diagnos* adj5 c?eliac?) or (comorbid* adj5 c?eliac)).ti,ab,kf. (42)

32 (high risk adj5 c?eliac?).ti,ab,kf. (67)

33 (famil* histor* adj2 c?eliac).ti,ab,kf. (33)

34 (children adj of* adj5 c?eliac).ti,ab,kw. (19)

35 or/30-34 (642)

36 10 or 29 or 35 (11362)

37 ((celiac* or coeliac*) adj2 (asymptomatic or atypical or adolesc* or adult* or cases or cohort* or child* or disease* or family or families or familial or genetic predispos* or genetic pre-dispos* or heredit* or infant* or men or patient* or people or population* or subjects or symptomatic or syndrom* or sprue or women)).ti,ab,kf. (18800)

38 limit 37 to ("in data review" or in process or publisher) (605)

39 36 or 38 (11704)

40 ((celiac* or coeliac*) adj (angiograp* or arter* or axis or plexus or trunk)).ti,ab,kf,hw. (8461)

41 case report.ti. (230986)

42 ((case adj of*) and (patient or man or woman)).ti,ab. and case reports.pt. (252275)

43 (comment or editorial or letter or newspaper article).pt. (1825467)

44 (exp Animals/ or exp Animal Experimentation/ or exp Models, Animal/ or exp Animals, Laboratory/ or exp rodentia/) not Humans/ (4673001)

45 ((rat or rats or mouse or mice or rodent* or animal* or murine or porcine or feline or canine or dog or dogs or cat or cats or pig or pigs or monkey* or macaque*) not human*).ti. (1856683)

46 or/40-45 (7137365)

47 39 not 46 (9645)

48 exp epidemiologic studies/ (2435409)

49 (cohort or case control* or case finding or cross-sectional or longitudinal).ti,ab,kf. (1114977)

50 study.ti. (1330964)

51 (study and (analys* or cases or clinical or control* or compar* or correlat* or associat* or epidemiolog* or evaluat* or examin* or investigat* or observ* or population based or prospectiv* or retrospectiv* or serolog*)).ti,ab,kf. (6665955)

52 (case? and control*).ab. (448997)

53 (controls or (control adj (group? or participants or patients))).ab. (1189049)

54 (groups or subgroup? or sub-group?).ab. (2170205)

55 (group? adj5 (analys* or compar* or control* or correlat* or associat*)).ab. (1004351)

56 exp Regression Analysis/ (416341)

57 statistical model/ (89771)

58 ((analys* or logistic*) adj1 (model* or regression*)).ab. (456097)

59 study.ab. /freq=2 (2157950)

60 or/48-59 (9618005)

61 47 and 60 (5419)

62 limit 61 to yr="1997 -Current" (4786)

****

Cochrane Library, Issue 2 of 12, 2020

#1 MeSH descriptor: [Celiac Disease] this term only [63]

#2 ("celiac disease" or "coeliac disease"): kw [338]

#3 ((celiac* or coeliac*) near/3 (asymptomatic or atypical or adolesc* or adult* or cases or cohort* or child* or disease* or family or families or familial or genetic predispos* or genetic pre-dispos* or heredit* or infant* or men or patient* or people or population* or subjects or symptomatic or syndrom* or sprue or women)):ti,ab [782]

#4 (#1 or #2 or #3) [886]

#5 ((celiac* or coeliac*) next (angiograp* or arter* or axis or plexus or trunk)):ti,ab,kw [276]

#6 #4 not #5 (n=870) [870]

****

Web of Science: Science Citation Index Expanded (SCI-EXPANDED), SCI-EXPANDED, Conference Proceedings Citation Index- Science SSCI

[Limit to celiac or coeliac in title/abstract, n=6118]

#28 (#26 not #27) [7,430]

#27 (TI=(“case report” or “a case of” or “a rare case of”)) or (TS=((“a case of” or “a rare case of”) near (patient or man or woman or child or adolescent or “an adult”))) [202,526]

#26 (#25 AND #24) [7493]
Indexes=SCI-EXPANDED, CPCI-S Timespan=1997-2020

#25 ((TS=(seroepidemiolog* or cohort or “case control*” or “case find*” or cross-sectional or longitudinal or “population based” or prospective* or retrospective*) or TI=(study) or TS=(study and (analys* or cases or clinical or control* or compar* or correlat* or associat* or epidemiolog* or evaluat* or examin* or investigat* or observ* or population based or prospectiv* or retrospectiv* or serolog*)) or TS=((case or cases) and control*) or TS=(controls or “control* group*” or “control* participant*” or “control* patient*” or groups or subgroup* or sub-group*) or TS=(group* near (analys* or compar* or control* or correlat* or associat*)) or TS=(“regression analysis” or “regression model*” or “statistical model*” or “logistic* model*”)) NOT (TI=(rat or rats or mouse or mice or rodent* or animal* or murine or porcine or feline or canine or dog or dogs or cat or cats or pig or pigs or monkey* or macaque* or nonhuman or “non human”))) [19,956,721
Indexes=SCI-EXPANDED, SSCI, A&HCI, CPCI-S, CPCI-SSH, ESCI Timespan=1900-2020

#24 (#23 OR #22 OR #21 OR #8) [13,272]

#23 (TS=((“high risk” or “famil* histor*” or “child* of”) near (celiac or coeliac)) or TS=(”celiac disease test kit”)) [233]
Indexes=SCI-EXPANDED, SSCI, A&HCI, CPCI-S, CPCI-SSH, ESCI Timespan=1900-2020

#22 (TS=((double* or dual*) near diagnos* near (celiac or coeliac))) or (TS=(comorbid* near (celiac or coeliac))) [111]
Indexes=SCI-EXPANDED, SSCI, A&HCI, CPCI-S, CPCI-SSH, ESCI Timespan=1900-2020

#21 (#20 AND #15 AND #1) [12,140]

#20 (#19 OR #18 OR #17 OR #16) [18,586]

#19 (TS=((“association* between” or “relationship between”) and (celiac or coeliac))) [1,458]
Indexes=SCI-EXPANDED, SSCI, A&HCI, CPCI-S, CPCI-SSH, ESCI Timespan=1900-2020

#18 ((TS=((association* or associated or indicator* or indicated or predict*) near (risk* or symptom* or disease* or disorder* or histor*))) and (TS=(celiac or coeliac))) [5,833]
Indexes=SCI-EXPANDED, SSCI, A&HCI, CPCI-S, CPCI-SSH, ESCI Timespan=1900-2020

#17 (TS=((celiac or coeliac) and ("detect* CD" or “CD detect*” or "detect* of CD" or "detect* with CD" or “diagnos* CD” or "CD diagnos*" or “diagnos* of CD” or "diagnos* with CD" or “predict* CD” or "CD predict*" or “predict* of CD” or "predict* with CD" or “inciden* CD” or "CD inciden*" or “inciden* of CD” or "inciden* with CD" or “prevalen* CD” or "CD prevalen*" or “prevalen* of CD” or " prevalen* with CD" or “seroprevalen* CD” or "CD seroprevalen*" or “seroprevalen* of CD” or "seroprevalen* with CD" or "CD risk*" or “risk* of CD” or “risk* for CD” or “risk* factor* of CD” or “risk factor* for CD” or “CD screen*” or "screen* for CD" or “CD test*” or "test* for CD"))) [1,489]
Indexes=SCI-EXPANDED, SSCI, A&HCI, CPCI-S, CPCI-SSH, ESCI Timespan=1900-2020

#16 (TS=(("case finding" or detect* or diagnos* or predict* or incidence or prevalence or seroprevalence* or risk or "risk factor*" or "risk indicator*" or screen* or test*) and (celiac* or coeliac*))) [17,056]
Indexes=SCI-EXPANDED, SSCI, A&HCI, CPCI-S, CPCI-SSH, ESCI Timespan=1900-2020

#15 (#14 OR #13 OR #12 OR #11 OR #10 OR #9) [14,174,134]

#14 (TI=(manifestation*)) [51,353]
Indexes=SCI-EXPANDED, SSCI, A&HCI, CPCI-S, CPCI-SSH, ESCI Timespan=1900-2020

#13 (TS=("first degree" near (related or relative* or relation*))) [11,114]
Indexes=SCI-EXPANDED, SSCI, A&HCI, CPCI-S, CPCI-SSH, ESCI Timespan=1900-2020

#12 (TS=("medical* morbid*" or (medical* near (comorbid* or co-morbid*)) or multimorbid* or "multi* morbid*" or "multi* comorbid*" or "multi* co-morbid*" or "multi* physical*")) [25,013]
Indexes=SCI-EXPANDED, SSCI, A&HCI, CPCI-S, CPCI-SSH, ESCI Timespan=1900-2020

#11 ((TS=(constipation or constipated or diarrhoea or diarrhea or (abdominal near (disten* or pain or bloating or cramp or cramps)) or flatulence or meteorism or steatorrhoea or steatorrhea)) or (TS=((“acid base” or calcium or cyanocobalamin* or electrolyte* or folate or "folic acid"or glucose or IgA or "immunoglobulin A" or iron or lactose or lipid* or phosph* or protein* or triglyceride* or vitamin* or mineral*) near (disorder* or deficien* or imbalance* or insufficiency or intoleran*))) or (TS=(fever* or febrile or malaise or fatigue or letharg* or exhaustion or vomiting or nausea or emesis or sickness or “weight loss” or “wait gain” or “hot flashes” or “hot flushes” or “medically unexplained” or “unexplained medical” or “unexplained symptom*” or “signs and symptoms” or

“condition* linked” or “linked condition*” or “condition* associated with” or sleepiness)) or (TS=((calciferol or cholecalciferol or colecalciferol or egocalciferol) near (disorder* or deficien* or imbalance* or insufficiency or intoleran*)))) or (TS=((anxiety or depression or mood) near (celiac or coeliac))) [1,055,350]
Indexes=SCI-EXPANDED, SSCI, A&HCI, CPCI-S, CPCI-SSH, ESCI Timespan=1900-2020

#10 (TS=("digestive system* disease*" or "digestive system* disorder*" or "digestive system* syndrom*" or "digestive system manifestation*" or "duoden* disease*" or "duoden* disorder*" or "duoden* syndrom*" or "duoden* manifestation*" or "gastr* disease*" or "gastr* disorder*" or "gastr* syndrom*" or "gastr* manifestation*" or "intestin* disease*" or "intestin* disorder*" or "intestin* syndrom*" or "intestin* manifestation*" or "malabsor* disease*" or "malabsor* disorder*" or "malabsor* syndrom*" or "malabsor* manifestation*")) [29,189]
Indexes=SCI-EXPANDED, SSCI, A&HCI, CPCI-S, CPCI-SSH, ESCI Timespan=1900-2020

#9 (TS=((addison* near (disease* or disorder* or syndrom*)) or alopecia or baldness or amenorrhea* or amenorrhoea* or oligomenorrhea* or oligomenorrhoea* or "anxiety disorder*" or anorexia or "adrenal insufficienc*" or "adrenocortical insufficienc*" or allerg* or anemi* or anaemi* or angina or aneurysm or "ankylosing spondylitis" or arthropath* or arthriti* or arthrosis or arthroses or asthma* or asthenia or ataxia or "atrial fibrillation" or atrophy or "autoimmune disorders" or "autoimmune diseases" or "autoimmune syndrom*" or ataxia or avitaminosis or "bonnevie ullrich" or "back pain" or (biliary near (disease* or disorder* or syndrom*)) or (bipolar near disorder*) or blindness or "blind loop syndrom*" or (blood near poisoning) or "brain atroph*" or ((bone or bones) near (broken or disease* or disorder* or syndrom*)) or (bone* near (mineral* or densit* or soft* or decay*)) or (brain near (disease* or disorder* or syndrom*)) or (bronchi* near (disease* or disorder* or syndrom*)) or (bowel* near (disease* or disorder* or syndrom*)) or calcinosis or cancer* or carcinoma* or adenocarcinoma* or neoplasm* or neoplastic or metasta* or malignan* or tumour or tumours or tumor or tumors or (cardiac near (arrest or arrhythmia* or disease* or disorder* or surg* or syndrom*)) or cardiomyopath* or ((cardiovascular or coronary) near (disease* or disorder* or event* or syndrom*)) or cachexia or ((cecal or colon* or duoden*) near (disease* or disorder* or syndrom*)) or "cerebral palsy" or (cerebro* near (degenerat* or disease* or disorder* or event* or syndrom*)) or "chronic obstructive disease" or cirrhosis or claudication or colic or COPD or ((coordination or co-ordination) near (impair* or lack)) or "congenital abnormalit*" or (congential near (disease or disorder* or syndrom*)) or ((connective or collagen* or skin) near (disease or disorder* or syndrom*)) or "collagenous sprue" or colitis or colotides or coxarthrosis or crohn* or cushing* or cyanosis or "cystic fibrosis" or cystitis or deaf* or deformit* or "delayed puberty" or dental or "depressive disorder*" or dysphoria or dysthymia or disabled or (physical near (deform* or disab* or impair*)) or dermatitis or dermato* or dorsopath* or diabet* or "down* syndrom*" or duhring* or duehring* or duhrig* or duehrig* or dyscoordination or dys-coordination or dys-coordination or dys-co-ordination or dysentery or dyssynergia or dys-synergia or emaciat* or enteritis or enterocolitis* or dystonia or eczema or edema or oedema or "elfin face sydrom*" or encephalopath* or (endocrine near (disease* or disorder* or syndrom*)) or enuresis or enteropath* or epilep* or (eye near (disease* or disorder* or manifestation* or syndrome*)) or "fatigue syndrome" or "chronic fatigue" or "failure to thrive" or fibromyalgia or fibrosis or "food hypersensitivity" or fractures or gammaglobulinemia or gammaglobulinaemia or (gardner* near (syndrom* or disease* or disorder*)) or gastritis or gastroenteritis or gout or (glomerul* near (disease* or disorder* or syndrom*)) or "gonodal near dysgen*" or (growth near (disease* or disorder* or syndrom*)) or headache* or ((hemic or haemic or lymph*) near (disease* or disorder* or syndrom*)) or hematuria or haematuria or hemophili* or haemophili* or ((hearing or visual or vision or sight) near (aid* or impair* or loss)) or (heart near

(disease* or disorder* or failure or syndrom* or manifestation* or myopathy*)) or hemiplegi* or hepatitis or hemodialysis or haemodialysis hiv or "human immunodeficiency virus" or "acquired immunodeficieny syndrom*" or "heerfordt waldenstrom*" or hidroa or hydroa or "hippel lindau*" or hypertensi* or hypotensi* or hyperthyroidism or hypothyroidism or hypocortisolism or hypocorticism or hypoadrenalism or incoordination or inco-ordination or in-coordination or in-co-ordination or infertility or subfertility or sub-fertil* or sterility or "inflammatory disease*" or "inflammatory bowel disease*" or incontinen* or "intestinal atresi*" or intussusception or "irritable bowel" or ischemi* or ischaemi* or fistula* or (joint near (disease* or disorder* or syndrom*)) or (jejunal near (disease* or disorder* or syndrom*)) or kyphosis or lav-htlv* or leukemia or leukaemia or ((liver or hepatic) near (disease* or disorder* or failure or syndrom*)) or lordosis or (lung near (disease* or disorder*)) or lupus or SLE or lymphoma or lymphogranuloma* or lymphadenopath* or lymphotrop* or "machado joseph*" or "macular degeneration" or malnutrition or (mental near (disorder* or disease* or health or illness*)) or mania or manic or (menstr* near (ceas* or disturb* or disease* or disorder* or stop* or syndrom*)) or migraine* or (mitochondrial near (disease* or disorder* or syndrom*)) or "movement disorder*" or mucinosis or musculoskeletal or "narp syndrom*" or necrotizing or nephrotic syndrom* or neuromuscular or non-hodgkin* or nonhodgkin* or "multiple sclerosis" or myeloma or myocarditis or myocardiopath* or myopath* or (myocardi* near (deteri* or disease* or disorder* or syndrom* or manifestation*)) or "nephrotic syndrome" or ((neurodegenerat* or neuro-degenerat) near (disease* or disorder* or syndrom)) or ((nutritional or metabolic) near (disease* or disorder* or syndrom*)) or ((organ* or kidney or "stem cell") near (transplant* or recipient*)) or ("nervous system" near (disease* or disorder* or syndrom*)) or (neurological near (condition* or disease* or disorder* or syndrom*)) or neuropath* or neurosarcoido* or occlusion* or obesity or obese or orthopedic* or orthopaedic* or ((esophageal or oesophageal) near (disease* or disorder* or syndrom*)) or ((oral or mouth) near (disease* or disorder* or syndrom* or manifestation*)) or osteo* or "otitis media" or otorhinolaryngolog* or otosclerosis or (pancrea* near (disease* or disorder* or syndrom*)) or papulosquamous or paraplegi* or parkinson* or (peripheral near (arterial or vascular or disease* or disorder* or syndrom*)) or (peritoneal near (disease* or disorder* or syndrom*)) or photodermato* or "pick disease*" or pneumo* or polio* or polyp* or polydipsia or polyarthropath* or polyarteritis or polyarthrosis or polyneuropath* or porphyrias or (pregnancy near complicat*) or "premature aging" or proteostasis or pseudophakia or psoriasis or parapsoriasis or prolapse or (pulmonary near (disease* or disorder* or syndrom*)) or purpur* or (recurren* near (abortion* or miscarr*)) or ((rectum or rectal) near (disease* or disorder* or syndrom*)) or ((renal or kidney) near (disease* or disorder* or failure or syndrom*)) or (respiratory near (disease* or disorder* or syndrom*)) or reticulocytosis or retinopathy or rheumat* or ricket* or sarcoido* or sepsis or septic* or seizure* or sclerosis or scoliosis or "sickle cell" or ((sjogren* or sjoegren* or sicca*) near (syndrom* or disease* or disorder*)) or ((skin or "connective tissue") near (disease* or disorder* or syndrom*)) or "sleep disorder*" or "sleep apnea" or "sleep apnoea" or insomnia* or dyssomnia* or hypersomnia* or "spina bifida" or "muscular atropy" or "short stature*" or "short bowel syndrom*" or (spin* near (degenerat* or disease* or disorder* or syndrom*)) or (spleen* near (disease* or disorder* or syndrom*)) or spondylo* or stenosis* or stoma* or (stomach near (disease* or disorder* or syndrom*)) or stroke or strokes or "cerebral infarct*" or tetraplegi* or thyroiditis* or (thyroid* near (disease* or disorder* or dysfunction* or syndrom*)) or ((tooth or teeth or enamel) near (decay* or discolo* or disease* or disorder* or dysfunction* or syndrom*)) or "tropical sprue" or tuberculosis or (systemic near (disorder* or disease* or syndrom*)) or thrombocyto* or tremor or tremors or (turner* near (syndrome* or disease* or disorder*)) or ulcer* or (urogenital near (disease* or disorder* or syndrom*)) or vasculopath* or (vascular near (disease* or disorder* or occlu* or syndrom*)) or vestibular or ((virus or viral or bacteri* or parasit*) near (infection* or disease*)) or

(wasting near (disorder* or disease* or syndrom*)) or (whipple near (disorder* or disease* or syndrom*)) or (william* near (syndrom* or disease* or disorder*)) or zoster*)) [13,693,359]
Indexes=SCI-EXPANDED, SSCI, A&HCI, CPCI-S, CPCI-SSH, ESCI Timespan=1900-2020

#8 (#7 and #1) [5,889]

#7 (#6 OR #5 OR #4 OR #3 OR #2) [6,827,636]

#6 (TS=("clinical decision making" or "logistic model*" or "statistical model*")) [81,189]
Indexes=SCI-EXPANDED, SSCI, A&HCI, CPCI-S, CPCI-SSH, ESCI Timespan=1900-2020

#5 (TS=((multivaria* or multicomponent*) NEAR (model* or algorithm* or score or scores or scoring or rule or rules or index or tool or tools or panel or criteri*))) [156,496]
Indexes=SCI-EXPANDED, SSCI, A&HCI, CPCI-S, CPCI-SSH, ESCI Timespan=1900-2020

#4 (TS=(clinic* NEAR (model* or algorithm* or score or scores or scoring or risk or risks or rule or rules or predict* or index or tool or tools or panel or criteri* or decision* or stratif* or diagnos* or detect* or identif* or probabilit*))) [1,061,406]
Indexes=SCI-EXPANDED, SSCI, A&HCI, CPCI-S, CPCI-SSH, ESCI Timespan=1900-2020

#3 (TS=((detect* or diagnos* or identif* or decision* or predict* or prognos* or stratif* or validat*) NEAR (risk or risks or model* or factor* or algorithm* or score or scores or scoring or system or technique* or aid or aids or rule or rules or index or variable* or tool or tools or panel or criteri* or characteristic* or history or finding* or value* or assay or stratif* or biomarker*))) [5,481,423]
Indexes=SCI-EXPANDED, SSCI, A&HCI, CPCI-S, CPCI-SSH, ESCI Timespan=1900-2020

#2 (TS=((detect* or diagnos* or identif* or decision* or predict* or prognos* or risk or risks or stratif* or validat*) NEAR (model* or factor* or algorithm* or score or scores or scoring or system or technique* or aid or aids or rule or rules or index or variable* or tool or tools or panel or criteri* or characteristic* or history or finding* or value* or assay or stratif* or biomarker*))) [6/133,858]
Indexes=SCI-EXPANDED, SSCI, A&HCI, CPCI-S, CPCI-SSH, ESCI Timespan=1900-2020

#1 TS=((celiac* OR coeliac*) NEAR (asymptomatic OR atypical OR adolesc* OR adult* OR cases OR cohort* OR child* OR disease* OR disorder* OR family OR families OR familial OR "genetic predispos*" OR "genetic pre-dispos*" OR heredit* OR infant* OR men OR patient* OR people OR population* OR subjects OR symptomatic OR syndrom* OR sprue OR women))) NOT (TS=((celiac* OR coeliac*) NEAR (angiography OR artery OR arteries OR axis OR plexus OR trunk))) [25,557]
Indexes=SCI-EXPANDED, SSCI, A&HCI, CPCI-S, CPCI-SSH, ESCI Timespan=1900-2020
