# Supplementary material for: Accuracy of potential diagnostic indicators for coeliac disease: a systematic review protocol
Source: BMJ Open. 2020 Oct 5;10(10):e038994. doi: 10.1136/bmjopen-2020-038994 (PMC7537462; doi:10.1136/bmjopen-2020-038994)
Supplement: Author's manuscript [file bmjopen-2020-038994.draft_revisions.pdf]

BMJ Open is committed to open peer review. As part of this commitment we make the peer review history of every article we publish publicly available.

When an article is published we post the peer reviewers' comments and the authors' responses online. We also post the versions of the paper that were used during peer review. These are the versions that the peer review comments apply to.

The versions of the paper that follow are the versions that were submitted during the peer review process. They are not the versions of record or the final published versions. They should not be cited or distributed as the published version of this manuscript.

BMJ Open is an open access journal and the full, final, typeset and author-corrected version of record of the manuscript is available on our site with no access controls, subscription charges or pay-per-view fees (http://bmjopen.bmj.com).

If you have any questions on BMJ Open's open peer review process please email info.bmjopen@bmj.com

Accuracy of potential diagnostic indicators for coeliac disease: a systematic review protocol

Journal:	*BMJ Open*
Manuscript ID	bmjopen-2020-038994
Article Type:	Protocol
Date Submitted by the Author:	31-Mar-2020

Complete List of Authors:	Elwenspoek, Martha; NIHR ARC West, ARC West; University of Bristol Medical School, Populations Health Sciences<br>Jackson, Joni; NIHR ARC West, ARC West; University of Bristol Medical School, Populations Health Sciences<br>Dawson, Sarah; NIHR ARC West, ARC West; University of Bristol Medical School, Populations Health Sciences<br>Everitt, Hazel; University of Southampton, Primary Care and Population Sciences, Faculty of Medicine<br>Gillett, Peter; Royal Hospital for Sick Children, Hepatology and Nutrition Department<br>Hay, Alastair; University of Bristol, Centre for Academic Primary Care<br>Jones, Hayley E.; Univ Bristol, ; University of Bristol<br>Lane, Deborah<br>Mallett, Susan; University of Birmingham, School of Health & Population Sciences<br>Robins, Gerry; York Teaching Hospital NHS Foundation Trust, Department of Gastroenterology<br>Sheppard, Athena; University of Leicester, Department of Health Sciences<br>Stubbs, Jo<br>Thom, H; University of Bristol Medical School, Populations Health Sciences<br>Watson, Jessica; University of Bristol, School of Social and Community Medicine<br>Whiting, Penny; University of Bristol, School of Social and Community Medicine

Keywords:	Coeliac disease < GASTROENTEROLOGY, Paediatric gastroenterology < PAEDIATRICS, Adult gastroenterology < GASTROENTEROLOGY

SCHOLARONE™
Manuscripts

I, the Submitting Author has the right to grant and does grant on behalf of all authors of the Work (as defined in the below author licence), an exclusive licence and/or a non-exclusive licence for contributions from authors who are: i) UK Crown employees; ii) where BMJ has agreed a CC-BY licence shall apply, and/or iii) in accordance with the terms applicable for US Federal Government officers or employees acting as part of their official duties; on a worldwide, perpetual, irrevocable, royalty-free basis to BMJ Publishing Group Ltd ("BMJ") its licensees and where the relevant Journal is co-owned by BMJ to the co-owners of the Journal, to publish the Work in this journal and any other BMJ products and to exploit all rights, as set out in our licence.

The Submitting Author accepts and understands that any supply made under these terms is made by BMJ to the Submitting Author unless you are acting as an employee on behalf of your employer or a postgraduate student of an affiliated institution which is paying any applicable article publishing charge ("APC") for Open Access articles. Where the Submitting Author wishes to make the Work available on an Open Access basis (and intends to pay the relevant APC), the terms of reuse of such Open Access shall be governed by a Creative Commons licence – details of these licences and which Creative Commons licence will apply to this Work are set out in our licence referred to above.

Other than as permitted in any relevant BMJ Author's Self Archiving Policies, I confirm this Work has not been accepted for publication elsewhere, is not being considered for publication elsewhere and does not duplicate material already published. I confirm all authors consent to publication of this Work and authorise the granting of this licence.

For peer review only

Accuracy of potential diagnostic indicators for coeliac disease: a systematic review protocol

PROSPERO registration number: CRD42020170766

Authors

Martha M C Elwenspoek,[1,2] Joni Jackson,[1,2] Sarah Dawson,[2] Hazel Everitt,[3] Peter Gillett,[4] Alastair D Hay,[2] Hayley E Jones,[2] Deborah Lane,[5] Susan Mallett,[6] Gerry Robins,[7] Athena Sheppard,[8] Jo Stubbs,[5] Howard Thom,[2] Jessica Watson,[2] Penny Whiting.[2]

Affiliations

1. The National Institute for Health Research Applied Research Collaboration West (NIHR ARC West), University Hospitals Bristol NHS Foundation Trust, BS1 2NT Bristol, UK
2. Population Health Sciences, Bristol Medical School, University of Bristol, BS8 2PS Bristol, UK
3. Primary Care, Population Sciences and Medical Education, University of Southampton, Southampton SO16 5ST, UK
4. Paediatric Gastroenterology, Hepatology and Nutrition Department, Royal Hospital for Sick Children, Edinburgh EH9 1LF Scotland, UK
5. Patient representative
6. The Institute of Applied Health Research, College of Medical and Dental Sciences, University of Birmingham, Edgbaston, Birmingham, B15 2TT, UK
7. Department of Gastroenterology, York Teaching Hospital NHS Foundation Trust, York, UK
8. Department of Health Sciences, University of Leicester, Leicester, UK

Corresponding author

Martha M C Elwenspoek
9th Floor, Whitefriars, Lewins Mead,
Bristol, BS1 2NT
Email: Martha.Elwenspoek@bristol.ac.uk
Tel: +44/0 117 3427689

Email addresses co-authors

Joni.Jackson@bristol.ac.uk, Sarah.Dawson@bristol.ac.uk, H.A.Everitt@soton.ac.uk, Peter.Gillett65@gmail.com, Alastair.Hay@bristol.ac.uk, Hayley.Jones@bristol.ac.uk, Deborah.L.Lane@googlemail.com, S.Mallett@bham.ac.uk, Gerry.Robins@York.nhs.uk, als75@leicester.ac.uk, Jo.Stubbs@bristol.ac.uk, Howard.Thom@bristol.ac.uk, Jessica.Watson@bristol.ac.uk, Penny.Whiting@bristol.ac.uk

Word count: 2842

For peer review only

Abstract

Introduction

Coeliac disease (CD) is a systemic immune-mediated disorder triggered by gluten in genetically predisposed individuals. CD is diagnosed using a combination of serology tests and endoscopic biopsy of the small intestine. However, because of nonspecific symptoms and heterogeneous clinical presentation, diagnosing CD is challenging. Early detection of CD through improved case-finding strategies can improve the response to a gluten-free diet, patients' quality of life, and potentially reduce the risk of complications. However, there is a lack of consensus which groups may benefit from active case-finding.

Methods and analysis

We will perform a systematic review to determine the accuracy of diagnostic indicators (such as symptoms and risk factors) for CD in adults and children, and thus can help identify patients who should be offered CD testing. MEDLINE, Embase, Cochrane Library, and Web of Science will be searched from 1997 until 2020. Screening will be performed in duplicate. Data extraction will be performed by one and checked by a second reviewer. Disagreements will be resolved through discussion or referral to a third reviewer. We will produce a narrative summary of identified prediction models. Studies where 2x2 data can be extracted or reconstructed will be treated as diagnostic accuracy studies, i.e. the diagnostic indicators are the index tests and CD serology and/or biopsy is the reference standard. For each diagnostic indicator, we will perform a bivariate random effects meta-analysis of the sensitivity and specificity.

Ethics and dissemination

Results will be reported in peer-reviewed journals, academic and public presentations and social media. We will convene an implementation panel to advise on the optimum strategy for enhanced dissemination. We will discuss findings with Coeliac UK to help with dissemination to patients. Ethical approval is not applicable, as this is a systematic review and no research participants will be involved.

Registration number

CRD42020170766

Keywords

Coeliac disease, diagnosis, risk factors, symptoms, systematic review protocol.

For peer review only

Article summary

Strengths and limitations of this study

- This systematic review protocol follows the Preferred Reporting Items for Systematic Review and Meta-Analysis Protocols guidelines, which offers transparency and enhances reproducibility.
- This review will follow guidelines from the Centre for Reviews and Dissemination and the Cochrane Handbook for Systematic Reviews of Diagnostic Test Accuracy, ensuring the use of robust and well-established methodologies.
- The information identified in the review will feed into economic models to determine the cost-effectiveness of active case-finding strategies, which will help commissioners, clinicians, and patients make evidence-based decisions on whether active case-finding for coeliac disease should be undertaken in primary care.
- We will include studies that use serology tests and/or biopsy as reference standard to increase the inclusion rate, but we note that serology tests in particular can produce both false positive and false negative results (i.e. are not a 'gold standard'), which may lead to bias in the primary analysis.
- By balancing the sensitivity and specificity of our search strategy there is a small risk of missing relevant papers, although this will be mitigated by screening reference lists of relevant reviews.

For peer review only

Introduction

Coeliac disease (CD) is a systemic, immune-mediated disorder triggered by the ingestion of the protein gluten and related prolamins, found in wheat, rye and barley, in genetically predisposed individuals.[1 2] CD is characterised by small intestine damage, gluten-dependent clinical symptoms, and increased blood levels of CD-specific antibodies.[1] Patients typically present with gastrointestinal symptoms such as chronic diarrhoea, abdominal pain, or constipation, but can also present with non-specific symptoms such as tiredness, anaemia, or impaired growth in children.[3] In the UK and other European countries, the lifetime prevalence is estimated at around 1%.[4-7] CD is more frequently diagnosed in 'at-risk' individuals with certain conditions, such as type 1 diabetes,[8] Down's syndrome,[9] or iron deficiency anaemia,[10] and is 1.5 to 2 times more common in women than in men.[11]

In first-degree relatives CD prevalence is as high as 10%[12] and concordance in identical twins is 75% higher compared to nonidentical twins,[13] suggesting a strong genetic component. Certain human leukocyte antigen (HLA) variants are strongly associated with CD and almost all CD patients carry the HLA-DQ2/8 haplotype. However, this risk haplotype is common; up to 40% of the general population carry the HLA-DQ2/8 haplotype – most of whom will never develop CD.[14] Although environmental factors such as gluten exposure, breast feeding, and gastrointestinal infections are reported to play a role in the aetiology of CD,[15] the exact mechanism and pathophysiology of CD remain unclear.

CD is diagnosed using a combination of serology tests and endoscopic biopsy of the small intestine. These tests are only reliable when the patient is on a gluten containing diet. Current guidelines by the National Institute for Health and Care Excellence (NICE) recommend testing for total immunoglobulin A (IgA) and IgA tissue transglutaminase (tTG) as a first choice in both adults and children.[16] In case of IgA deficiency, immunoglobulin G (IgG) tTG, IgG endomysial antibody (EMA), or IgG deamidated gliadin peptide (DGP) can be measured. Seropositive adults should be referred for intestinal biopsy. Seropositive children should be referred for further investigation, which may include IgA EMA, intestinal biopsy, HLA genetic testing, or a combination thereof.[16]

The European Society for Paediatric Gastroenterology Hepatology and Nutrition (EPSGHAN) launched new guidelines in 2012[17] to avoid biopsies in children with high IgA tTG titres (>10 times upper limit of normal) and positive HLA-DQ2/8 and EMA results. Since then, new findings have supported this approach.[18] The updated EPSGHAN guidelines (2019) suggest that HLA-DQ2/8 testing can be omitted and the biopsy avoidance strategy can also be used in asymptomatic patients.[18] The biopsy avoidance strategy has been endorsed by the British Society of Paediatric Gastroenterology Hepatology and Nutrition (BSPGHAN) and Coeliac UK (2013) joint Guidelines for the diagnosis and management of coeliac disease in children,[4] but not yet by the NICE guidelines.[3] For adults, the biopsy avoidance strategy is currently not recommended.[3 19] However, a recent study suggests that adult CD can also be reliably diagnosed without biopsy in patients with high IgA tTG levels (>10× the upper limit of normal), positive EMA and HLA-DQ2/8 test results without requirement of symptoms.[20]

Patients diagnosed with CD are advised to follow a strict gluten-free diet, which is expensive and challenging to adhere to for patients, but it is the only available treatment. In most symptomatic patients a gluten-free diet reduces symptoms, increases quality of life, and seems to lower the risk of complications.[21-23] However, because of the nonspecific symptoms and heterogeneous clinical presentation, diagnosing CD is challenging. As a consequence, mean duration of symptoms before diagnosis is as long as 13 years in the UK[24] and 11 years in the US.[25] A delayed diagnosis can increase

For peer review only

the risk of more severe or non-responsive CD,[26] as well as the risk of severe complications such as osteoporosis,[27] infertility,[28] and lymphoma.[29]

Active case-finding strategies, i.e. offering tests to individuals with certain symptoms or conditions associated with CD, aim to improve timeliness of CD diagnosis. Early detection of CD through improved case-finding strategies can improve the response to a gluten-free diet, patients' quality of life, and potentially reduce the risk of complications. However, there is a lack of consensus which groups may benefit from active case-finding. Therefore, we will perform a systematic review of the literature to investigate which diagnostic indicators (such as symptoms and risk factors) are most sensitive and specific for CD, and thus can help identify patients that should be offered CD testing.

Aim

The overall aim of this review is to determine the accuracy of diagnostic indicators for CD in adults and children.

Objectives

1. Determine the accuracy of diagnostic indicators to diagnose CD in children (≤ 16 years old).
2. Determine the accuracy of diagnostic indicators in adults (> 16 years old).
3. Identify combinations of diagnostic indicators that lead to an increased probability of coeliac disease.

For peer review only

Methods

The review will follow Centre for Reviews and Dissemination (CRD) guidelines for undertaking systematic reviews[30] and methods described in the Cochrane Handbook for Systematic Reviews of Diagnostic Test Accuracy[31] and has been registered on the PROSPERO database (CRD42020170766). This protocol is reported according to the PRISMA guidance for systematic review protocols and the PRISMA statement for diagnostic test accuracy studies.[32 33] Protocol amendments will be documented and published together with the results of the systematic review.

Eligibility criteria

Studies that fulfil the following criteria will be eligible for inclusion:

- **Population**: Adults and/or children (≤ 16 years old) tested for CD.
- **Diagnostic indicators (treated as 'index tests')**: Any diagnostic indicator will be eligible for inclusion. We consider diagnostic indicators to be those that may assist a clinician in making a diagnosis during an initial consultation, for example: family history or recent test results (e.g. full blood count to test for anaemia), current symptoms, or a risk HLA-DQ genotype. Factors that are more general risk factors rather than potential diagnostic indicators such as perinatal factors, 'susceptibility genes' (other than HLA-DQ status), or age at gluten introduction will be excluded. Tests for susceptibility genes are currently not widely available to clinicians and therefore not (yet) useful in aiding diagnosis.
- **Reference standard:** Serological tests and/or duodenal biopsy. Studies where not all patients have received the reference standard will be excluded.
- **Study design**: Studies using a "single-gate" or "multi-gate" design and prediction modelling studies are eligible for inclusion. In a single-gate design, a single set of inclusion criteria (gate) is used for all participants, whereas in a multi-gate design, two or more sets of inclusion criteria (gates) are used (e.g. one for a risk factor group and another for healthy controls).[34] Study participants without CD (e.g. a control group) are required to be representative of the general population. Diagnostic indicator studies will be treated as diagnostic test accuracy studies and we will refer to these studies as such throughout this protocol. If sufficient single-gate studies are identified, then multi-gate studies will be excluded, as these have been shown to overestimate test accuracy.[35] Diagnostic accuracy studies will be required to report sufficient data to construct 2x2 tables showing the number of participants with and without CD (according to the reference standard) cross-classified against the number with and without the diagnostic indicator. There will be no specific data requirements for prediction modelling studies.
- **Additional restrictions**: We will exclude studies published before 1997 (the year in which the most important serological test, tTG, was developed), to reduce the variation in the methods used for diagnosing coeliac disease (reference standard). We will not apply any restrictions based on patient age, language, or study size.

Information sources

MEDLINE, Embase, Cochrane Library, and Web of Science will be searched. Ongoing and completed studies will be identified using the WHO International Clinical Trials Registry and the NIH Clinical Trials database, and internet searches will be undertaken. Reference lists of the latest guidelines from NICE and the British Society of Gastroenterology on CD and systematic reviews published after the latest NICE guidelines will be screened.

For peer review only

Search strategy

The search strategy incorporates three main elements: (1) conditions (CD) + prognostic/predictive research filter (based on Geersing[36] and Ingui's[37] work), (2) conditions (CD) + all physical diseases/ signs/symptoms (based on MeSH, EMTREE) + 'CD' diagnosis, (3) terms for high risk populations (see Supplementary material for a detailed search strategy). We will exclude animal studies, case reports, letters, editorials, and coeliac artery/trunk research and will apply a sensitive study design filter. No language or publication restrictions will be applied.

Study records

Data management

Identified references will be downloaded into Endnote X9 software for further assessment and handling. Rigorous records are maintained as part of the searching process.

Selection process

Study selection will be conducted in two stages using forms developed in Microsoft Access: (1) an initial screening of titles and abstracts against the inclusion criteria to identify potentially relevant papers, (2) screening of the full papers identified as possibly relevant in the initial screening. All papers excluded at the second stage will be documented along with the reasons for exclusion. Abstracts and full texts will be screened independently by at least two researchers. Disagreements about study eligibility will be resolved through discussion or by consulting a third reviewer.

Data collection process (data extraction)

Data will be extracted using standardised data extraction forms developed in Microsoft Access. Data extraction forms will be piloted on a small sample of papers and adapted as necessary. In order to minimise bias and errors, data extraction will be performed by one reviewer and checked by a second. Disagreements will be resolved through discussion or referral to a third reviewer.

To exclude diagnostic indicators with insufficient evidence, we will only extract data on diagnostic indicators that are reported in five or more studies, with the exception of "new but exceptionally promising" diagnostic indicators according to our expert panel (i.e. widely used diagnostic indicators that are deemed too important for omission). We will extract the following data from the remaining papers, where reported: study characteristics, patient characteristics, details on the diagnostic indicator, and details on how CD was diagnosed (i.e. the reference standard).

Diagnostic accuracy studies: Numerical results will be extracted as 2x2 contingency tables (i.e. number of individuals with versus without the diagnostic indicator, tabulated against CD status). Where possible, data will be extracted separately for different age groups (children and adults), sex (male and female), and symptomatic groups ("at risk" asymptomatic, symptomatic primary care or symptomatic secondary care). If 2x2 data is not reported, we will reconstruct this from estimates of sensitivity, specificity, positive and negative predictive values, or unadjusted diagnostic odds ratios, intercept, and prevalence data where possible. If this is not possible the study will be excluded. We will contact authors if there are inconsistencies in the data.

Prediction modelling studies: We will extract details from the final model, including which variables were used, whether the model was validated, and measures of model performance. If 2x2 data (or sufficient data from which to recover this, such as unadjusted ORs, intercepts, prevalence) are reported for individual diagnostic indicators, these will also be extracted.

For peer review only

Risk of bias in individual studies

As we are treating diagnostic indicator studies as diagnostic test accuracy studies, risk of bias will be assessed using the QUADAS-2 tool[38] or QUADAS-3 tool if available (currently under development), which includes domains covering participants, index test, reference standard and flow and timing. Prediction modelling studies will be assessed using the PROBAST tool,[39] which includes domains covering participant selection, outcome, predictors, sample size and flow, and analysis. The content of the tools will be tailored to the review by adding guidance for interpreting signalling questions as appropriate. The tailored tools will be combined and piloted on a small number of studies by two reviewers and agreement assessed. If agreement is low, the tool and/or guidelines will be further refined until satisfactory agreement is reached and the tool can be applied to all included studies. If at least one of the domains is rated as "high risk" the study will be considered at high risk of bias, if all domains are judged as "low risk" the trial will be considered at low risk of bias, otherwise the trial will be considered at "unclear" risk of bias. The risk of bias assessment will be conducted as part of the data extraction process.

Data synthesis

A narrative summary of the included studies will be presented. This will include a summary of the characteristics (e.g. study design, population size, geographical location, year, baseline population characteristics, diagnostic indicators evaluated, and reference standard). A detailed commentary on the major methodological problems or biases that affected the studies will also be included.

The analysis will be stratified by age group (children ≤ 16 years old, adults, mixed) and we will group study results according to type of diagnostic indicator (symptoms, risk conditions, genetic risk factors). For each group, we will plot study-specific estimates of sensitivity and specificity in receiver operating characteristic (ROC) space and/or produce coupled forest plots.

To exclude diagnostic indicators with insufficient evidence, we will only perform meta-analyses on diagnostic indicators where 2x2 data is available from five studies or more. We will perform a bivariate random effects meta-analysis of the sensitivity and specificity on each remaining diagnostic indicator.[40 41] This will produce summary estimates of the sensitivity and specificity of each diagnostic indicator, with 95% confidence or credible ellipses, and 95% prediction ellipses. Prediction ellipses are visual representations of the amount of between-study heterogeneity in both dimensions of a diagnostic test meta-analysis. We will also report estimates of the between-study standard deviation in sensitivity and specificity on the logit scale.

Summary results from each meta-analysis will also be used to estimate positive and negative predictive values (PPVs and NPVs), i.e. the probability of CD given that the individual has (or does not have) each diagnostic indicator. Calculation of these values will require an assumed value for the prevalence of CD in the general population. This could be based on studies in the meta-analysis, or on other evidence. If estimates of prevalence are heterogeneous, we will present data or plot predictive values across a range of plausible prevalences.

Exploration of heterogeneity: We expect heterogeneity in study estimates of sensitivity and specificity due to variability in patient populations, reference standard definitions or methods, study design, and other study characteristics.

If sufficient data are available, we will use subgroup analysis and/or meta-regression to investigate whether the identified diagnostic indicators differ in accuracy between: (i) setting (asymptomatic "at risk", primary care symptomatic, and secondary care symptomatic patients), (ii) children vs adults

For peer review only

(iii) CD diagnosis (biopsy and serology versus serology only), (iv) study design (single-gate versus multi-gate), (v) men versus women, (vi) low versus high risk of bias. We will also explore the possibility of adjusting for the imperfect accuracy of the serological tests in a Bayesian statistical framework, using informative prior distributions for the sensitivity and specificity of these tests based on an ongoing systematic review on the accuracy of serology tests for CD.[42]

All statistical analyses for will be performed in R and WinBUGS (version 14).

Prediction modelling studies: We will also produce a narrative summary of prediction models, describing which variables were included in the final model, measures of model performance, and whether the model was validated.

Patient and public involvement

Our team includes two patient representatives who were involved in development of the research project, of which this review is a part. The patient representatives will be invited to join regular team meetings to provide patient perspectives on the research to ensure that it addresses patient needs. They will also be involved in the interpretation and dissemination of the results from this review.

Dissemination of findings

Results will be reported in peer-reviewed journals, academic, and public presentations and social media. Results will also be expressed in terms of hypothetical populations of 1000 patients for a plain language summary of analysis results. We will convene an implementation panel to advise on the optimum strategy for enhanced dissemination. We will discuss findings with the charity Coeliac UK to help with dissemination to patients.

Timeline

**Task**	**Deadline**
Protocol development	February 2020
Searches	February 2020
Abstract and full text screening	April 2020
Data extraction	September 2020
Analysis	January 2021
Manuscript submission	April 2021

For peer review only

Discussion

This systematic review is part of a larger project that aims to identify cost-effective strategies for active case-finding of CD in different at-risk populations. The information identified in the review will feed into economic models to determine the cost-effectiveness of active case-finding strategies, which will help commissioners, clinicians, and patients make evidence-based decisions on whether active case-finding for CD should be undertaken in primary care. There is evidence that CD is under-diagnosed in both children and adults.[6 43] Appropriate identification and treatment of CD can have significant benefits for patients in terms of symptoms, quality of life, and long-term health outcomes, as well as reducing healthcare and societal economic costs.[24 44] Different guidelines recommend different diagnostic pathways showing a lack of evidence and consensus on which groups may benefit from active case-finding, the best method of doing this, and the costs, benefits and potential harms. A rigorously undertaken high-quality evidence synthesis including health economic modelling will provide a robust summary of the current evidence base and highlight whether there is sufficient evidence to suggest an optimum strategy or whether further research is needed.

For peer review only

Funding statement

This work was supported by a National Institute for Health Research (NIHR) Health Technology Assessment Programme grant number NIHR129020. This publication presents independent research funded by the NIHR. This research was also supported by the National Institute for Health Research (NIHR) Applied Research Collaboration West (NIHR ARC West). The views expressed in this article are those of the author(s) and not necessarily those of the NIHR or the Department of Health and Social Care.

Competing interest statement

No competing interests.

Author contributions

PW conceptualised and designed the protocol. ME drafted the initial manuscript. SD, ME, and PW defined the concepts and search items. PW, ME, HJ, and SM planned the data extraction and statistical analysis. HE, PG, AH, GR, and JW provided clinical insights and DL and JS provided patient perspectives. All authors reviewed the manuscript and have approved and contributed to the final written manuscript.

For peer review only

References

1. Lindfors K, Ciacci C, Kurppa K, et al. Coeliac disease. *Nature Reviews Disease Primers* 2019;5(1):3. doi: 10.1038/s41572-018-0054-z
2. Ludvigsson JF, Leffler DA, Bai JC, et al. The Oslo definitions for coeliac disease and related terms. *Gut* 2013;62(1):43-52. doi: 10.1136/gutjnl-2011-301346 [published Online First: 2012/02/22]
3. Internal Clinical Guidelines Team 2015. Clinical Guideline NG20 Coeliac Disease: Recognition, assessment and management. *National Institute for Health and Care Excellence (NICE),* 2015
4. Murch S, Jenkins H, Auth M, et al. Joint BSPGHAN and Coeliac UK guidelines for the diagnosis and management of coeliac disease in children. *Arch Dis Child* 2013;98(10):806-11. doi: 10.1136/archdischild-2013-303996 [published Online First: 2013/08/30]
5. Mustalahti K, Catassi C, Reunanen A, et al. The prevalence of celiac disease in Europe: results of a centralized, international mass screening project. *Ann Med* 2010;42(8):587-95. doi: 10.3109/07853890.2010.505931 [published Online First: 2010/11/13]
6. Bingley PJ, Williams AJ, Norcross AJ, et al. Undiagnosed coeliac disease at age seven: population based prospective birth cohort study. *BMJ* 2004;328(7435):322-3. doi: 10.1136/bmj.328.7435.322 [published Online First: 2004/02/07]
7. West J, Logan RF, Hill PG, et al. Seroprevalence, correlates, and characteristics of undetected coeliac disease in England. *Gut* 2003;52(7):960-5. doi: 10.1136/gut.52.7.960 [published Online First: 2003/06/13]
8. Ludvigsson JF, Ludvigsson J, Ekbom A, et al. Celiac disease and risk of subsequent type 1 diabetes: a general population cohort study of children and adolescents. *Diabetes Care* 2006;29(11):2483-8. doi: 10.2337/dc06-0794 [published Online First: 2006/10/27]
9. Marild K, Stephansson O, Grahnquist L, et al. Down syndrome is associated with elevated risk of celiac disease: a nationwide case-control study. *J Pediatr* 2013;163(1):237-42. doi: 10.1016/j.jpeds.2012.12.087 [published Online First: 2013/02/13]
10. Hin H, Bird G, Fisher P, et al. Coeliac disease in primary care: case finding study. *BMJ* 1999;318(7177):164-7. doi: 10.1136/bmj.318.7177.164 [published Online First: 1999/01/15]
11. Murray JA, Van Dyke C, Plevak MF, et al. Trends in the identification and clinical features of celiac disease in a North American community, 1950-2001. *Clin Gastroenterol Hepatol* 2003;1(1):19-27. doi: 10.1053/jcgh.2003.50004 [published Online First: 2004/03/16]
12. Rubio-Tapia A, Van Dyke CT, Lahr BD, et al. Predictors of family risk for celiac disease: a population-based study. *Clin Gastroenterol Hepatol* 2008;6(9):983-7. doi: 10.1016/j.cgh.2008.04.008 [published Online First: 2008/07/01]
13. Greco L, Romino R, Coto I, et al. The first large population based twin study of coeliac disease. *Gut* 2002;50(5):624-8. doi: 10.1136/gut.50.5.624 [published Online First: 2002/04/16]
14. Jamnik J, Villa CR, Dhir SB, et al. Prevalence of positive coeliac disease serology and HLA risk genotypes in a multiethnic population of adults in Canada: a cross-sectional study. 2017;7(10):bmjopen-2017-017678. doi: 10.1136/bmjopen-2017-017678 %J BMJ Open
15. Meijer C, Shamir R, Szajewska H, et al. Celiac Disease Prevention. *Front Pediatr* 2018;6:368. doi: 10.3389/fped.2018.00368 [published Online First: 2018/12/18]
16. Downey L, Houten R, Murch S, et al. Recognition, assessment, and management of coeliac disease: summary of updated NICE guidance. *BMJ* 2015;351:h4513. doi: 10.1136/bmj.h4513 [published Online First: 2015/09/04]
17. Husby S, Koletzko S, Korponay-Szabo IR, et al. European Society for Pediatric Gastroenterology, Hepatology, and Nutrition guidelines for the diagnosis of coeliac disease. *J Pediatr Gastroenterol Nutr* 2012;54(1):136-60. doi: 10.1097/MPG.0b013e31821a23d0 [published Online First: 2011/12/27]
18. Husby S, Koletzko S, Korponay-Szabo I, et al. European Society Paediatric Gastroenterology, Hepatology and Nutrition Guidelines for Diagnosing Coeliac Disease 2020. *J Pediatr*

For peer review only

Gastroenterol Nutr 2020;70(1):141-56. doi: 10.1097/MPG.0000000000002497 [published Online First: 2019/10/01]

19. Ludvigsson JF, Bai JC, Biagi F, et al. Diagnosis and management of adult coeliac disease: guidelines from the British Society of Gastroenterology. *Gut* 2014;63(8):1210-28. doi: 10.1136/gutjnl-2013-306578 [published Online First: 2014/06/12]
20. Fuchs V, Kurppa K, Huhtala H, et al. Serology-based criteria for adult coeliac disease have excellent accuracy across the range of pre-test probabilities. *Aliment Pharmacol Ther* 2019;49(3):277-84. doi: 10.1111/apt.15109 [published Online First: 2018/12/29]
21. Murray JA, Watson T, Clearman B, et al. Effect of a gluten-free diet on gastrointestinal symptoms in celiac disease. *Am J Clin Nutr* 2004;79(4):669-73. doi: 10.1093/ajcn/79.4.669 [published Online First: 2004/03/31]
22. Sanchez MI, Mohaidle A, Baistrocchi A, et al. Risk of fracture in celiac disease: gender, dietary compliance, or both? *World J Gastroenterol* 2011;17(25):3035-42. doi: 10.3748/wjg.v17.i25.3035 [published Online First: 2011/07/30]
23. Holmes GK, Prior P, Lane MR, et al. Malignancy in coeliac disease--effect of a gluten free diet. *Gut* 1989;30(3):333-8. doi: 10.1136/gut.30.3.333 [published Online First: 1989/03/01]
24. Gray AM, Papanicolas IN. Impact of symptoms on quality of life before and after diagnosis of coeliac disease: results from a UK population survey. *BMC Health Serv Res* 2010;10:105. doi: 10.1186/1472-6963-10-105 [published Online First: 2010/04/29]
25. Green PHR, Stavropoulos SN, Panagi SG, et al. Characteristics of adult celiac disease in the USA: results of a national survey. *Am J Gastroenterol* 2001;96(1):126-31. doi: 10.1111/j.1572-0241.2001.03462.x [published Online First: 2001/02/24]
26. Paarlahti P, Kurppa K, Ukkola A, et al. Predictors of persistent symptoms and reduced quality of life in treated coeliac disease patients: a large cross-sectional study. *BMC Gastroenterol* 2013;13:75. doi: 10.1186/1471-230X-13-75 [published Online First: 2013/05/02]
27. Capriles VD, Martini LA, Areas JA. Metabolic osteopathy in celiac disease: importance of a gluten-free diet. *Nutr Rev* 2009;67(10):599-606. doi: 10.1111/j.1753-4887.2009.00232.x [published Online First: 2009/09/30]
28. Gasbarrini A, Torre ES, Trivellini C, et al. Recurrent spontaneous abortion and intrauterine fetal growth retardation as symptoms of coeliac disease. *Lancet* 2000;356(9227):399-400. doi: 10.1016/S0140-6736(00)02535-6 [published Online First: 2000/09/06]
29. Elfstrom P, Granath F, Ekstrom Smedby K, et al. Risk of lymphoproliferative malignancy in relation to small intestinal histopathology among patients with celiac disease. *J Natl Cancer Inst* 2011;103(5):436-44. doi: 10.1093/jnci/djq564 [published Online First: 2011/02/04]
30. Centre for Reviews and Dissemination (CRD). Systematic Reviews. CRD's guidance for undertaking reviews in health care. *University of York* 2009
31. Cochrane Diagnostic Test Accuracy Working Group. Cochrane Handbook for Systematic Reviews of Diagnostic Test Accuracy. *The Cochrane Collaboration* 2010
32. Shamseer L, Moher D, Clarke M, et al. Preferred reporting items for systematic review and meta-analysis protocols (PRISMA-P) 2015: elaboration and explanation. *BMJ* 2015;350:g7647. doi: 10.1136/bmj.g7647 [published Online First: 2015/01/04]
33. McInnes MDF, Moher D, Thombs BD, et al. Preferred Reporting Items for a Systematic Review and Meta-analysis of Diagnostic Test Accuracy Studies: The PRISMA-DTA Statement. *JAMA* 2018;319(4):388-96. doi: 10.1001/jama.2017.19163 [published Online First: 2018/01/25]
34. Rutjes AW, Reitsma JB, Vandenbroucke JP, et al. Case-control and two-gate designs in diagnostic accuracy studies. *Clin Chem* 2005;51(8):1335-41. doi: 10.1373/clinchem.2005.048595 [published Online First: 2005/06/18]
35. Whiting PF, Rutjes AW, Westwood ME, et al. A systematic review classifies sources of bias and variation in diagnostic test accuracy studies. *J Clin Epidemiol* 2013;66(10):1093-104. doi: 10.1016/j.jclinepi.2013.05.014 [published Online First: 2013/08/21]

For peer review only

36. Geersing GJ, Bouwmeester W, Zuithoff P, et al. Search filters for finding prognostic and diagnostic prediction studies in Medline to enhance systematic reviews. *PLoS One* 2012;7(2):e32844. doi: 10.1371/journal.pone.0032844 [published Online First: 2012/03/07]
37. Ingui BJ, Rogers MA. Searching for clinical prediction rules in MEDLINE. *J Am Med Inform Assoc* 2001;8(4):391-7. doi: 10.1136/jamia.2001.0080391 [published Online First: 2001/06/22]
38. Whiting PF, Rutjes AW, Westwood ME, et al. QUADAS-2: a revised tool for the quality assessment of diagnostic accuracy studies. *Ann Intern Med* 2011;155(8):529-36. doi: 10.7326/0003-4819-155-8-201110180-00009 [published Online First: 2011/10/19]
39. Wolff RF, Moons KGM, Riley RD, et al. PROBAST: A Tool to Assess the Risk of Bias and Applicability of Prediction Model Studies. *Ann Intern Med* 2019;170(1):51-58. doi: 10.7326/M18-1376 [published Online First: 2019/01/01]
40. Reitsma JB, Glas AS, Rutjes AW, et al. Bivariate analysis of sensitivity and specificity produces informative summary measures in diagnostic reviews. *J Clin Epidemiol* 2005;58(10):982-90. doi: 10.1016/j.jclinepi.2005.02.022 [published Online First: 2005/09/20]
41. Chu H, Cole SR. Bivariate meta-analysis of sensitivity and specificity with sparse data: a generalized linear mixed model approach. *J Clin Epidemiol* 2006;59(12):1331-2; author reply 32-3. doi: 10.1016/j.jclinepi.2006.06.011 [published Online First: 2006/11/14]
42. Sheppard AL, Whiting P, Corfield V, et al. What is the accuracy of serological testing for diagnosing coeliac disease in adults and children? A systematic review: PROSPERO, 2019.
43. Ravikumara M, Nootigattu VK, Sandhu BK. Ninety percent of celiac disease is being missed. *J Pediatr Gastroenterol Nutr* 2007;45(4):497-9. doi: 10.1097/MPG.0b013e31812e5710 [published Online First: 2007/11/22]
44. Mearns ES, Taylor A, Boulanger T, et al. Systematic Literature Review of the Economic Burden of Celiac Disease. *Pharmacoeconomics* 2019;37(1):45-61. doi: 10.1007/s40273-018-0707-5 [published Online First: 2018/09/18]

For peer review only

Supplementary materials

Search strategy

Database: Embase <1974 to 2020 Week 05>

Search Strategy: *ARC-Coeliac-Embase-Final*

----

1 Celiac Disease/ (29729)

2 c?eliac.ti,kw. (22813)

3 c?eliac.af. and gastroenterology.ec. (22352)

4 (celiac or coeliac).ab. /freq=3 (9328)

5 or/1-4 (39462)

6 ((detect* or diagnos* or identif* or decision* or predict* or prognos* or risk or risks or stratif* or validat*) adj3 (model* or factor* or algorithm? or scor* or system or technique? or aid? or rule or rules or index or variable* or tool? or panel or criteri* or characteristic? or history or finding* or value* or assay or stratif* or biomarker?)).ti,ab,kw. (2855495)

7 ((detect* or diagnos* or identif* or decision* or predict* or prognos* or stratif* or validat*) adj3 (risk or risks or model* or factor* or algorithm? or scor* or system or technique? or aid? or rule or rules or index or variable* or tool? or panel or criteri* or characteristic? or history or finding* or value* or assay or stratif* or biomarker?)).ti,ab,kw. (2225766)

8 (clinic* adj3 (model* or algorithm? or scor* or risk or risks or rule or rules or predict* or index or tool? or panel or criteri* or decision* or stratif* or diagnos* or detect* or identif* or probabilit*)).ti,ab,kw. (632961)

9 ((multivaria* or multicomponent?) adj3 (model* or algorithm? or scor* or rule or rules or index or tool? or panel or criteri*)).ti,ab,kw. (110662)

10 clinical decision making/ or statistical model/ or algorithm/ (444594)

11 or/6-10 (3678637)

12 5 and 11 (6036)

13 *physical disease by body function/ or exp *abnormal blood pressure/ or exp *abnormal posture/ or *aerophagia/ or exp *appetite disorder/ or *asthenia/ or *asymptomatic disease/ or exp *autonomic dysfunction/ or exp *balance disorder/ or exp *blood clotting disorder/ or exp *body temperature disorder/ or exp *body weight disorder/ or exp *common cold symptom/ or exp *consciousness disorder/ or exp *constipation/ or exp *coughing/ or exp *cyanosis/ or exp *diarrhea/ or exp *disability/ or exp *"disorders of higher cerebral function"/ or exp *dysphagia/ or *eructation/ or exp *faintness/ or exp *fatigue/ or *functional disease/ or exp *growth disorder/ or exp *immunopathology/ or exp *incontinence/ or exp *infertility/ or *listlessness/ or *malaise/ or *meningism/ or exp *metabolic disorder/ or exp *micturition disorder/ or exp *motor dysfunction/ or exp *multiple organ failure/ or exp *"nausea and vomiting"/ or exp *nutritional disorder/ or exp

For peer review only

*pain/ or *pallor/ or exp *pregnancy disorder/ or *qi deficiency/ or *qi stagnation/ or exp *reflex disorder/ or exp *respiratory function disorder/ or exp *salivation disorder/ or exp *sensory dysfunction/ or exp *sexual dysfunction/ or exp *shock/ or exp *sleep disorder/ or exp *speech disorder/ or *weakness/ or *yang deficiency/ or *yin deficiency/ (4991033)

14 *physical disease by anatomical structure/ or exp *abdominal disease/ or exp *abnormal body build/ or exp *breast disease/ or exp *cardiovascular disease/ or exp *connective tissue disease/ or exp *digestive system disease/ or exp *ear nose throat disease/ or exp *endocrine disease/ or exp *eye disease/ or exp *"head and neck disease"/ or exp *hematologic disease/ or exp *mouth disease/ or exp *mucosal disease/ or exp *musculoskeletal disease/ or exp *neurologic disease/ or exp *pelvic disease/ or exp *respiratory tract disease/ or exp *skin disease/ or exp *soft tissue disease/ or exp *thorax disease/ or exp *urogenital tract disease/ (11959905)

15 *"physical disease by composition of body fluids, excreta and secretions"/ or exp *abnormal feces composition/ or exp *abnormal substrate concentration in blood/ or exp *abnormal urine composition/ or *dehydration/ or *hypervolemia/ (344197)

16 ((addison* adj3 (disease* or disorder* or syndrom*)) or alopecia or baldness or amenorrhea* or amenorrhoea* or oligomenorrhea* or oligomenorrhoea* or anxiety disorder* or anorexia or adrenal insufficienc* or adrenocortical insufficienc* or allerg* or anemi* or anaemi* or angina or aneurysm or ankylosing spondylitis or arthropath* or arthriti* or arthrosis or arthroses or asthma* or asthenia or ataxia or atrial fibrillation or atrophy or autoimmune disorder* or (autoimmune adj (disease* or disorder* or syndrom*)) or ataxia or avitaminosis or bonnevie ullrich or back pain or (biliary adj3 (disease* or disorder* or syndrom*)) or (bipolar adj3 disorder*) or blindness or blind loop syndrom* or (blood adj3 poisoning) or brain atroph* or ((bone or bones) adj3 (broken or disease* or disorder* or syndrom*)) or (bone* adj3 (mineral* or densit* or soft* or decay*)) or (brain adj3 (disease* or disorder* or syndrom*)) or (bronchi* adj3 (disease* or disorder* or syndrom*)) or (bowel* adj3 (disease* or disorder* or syndrom*)) or calcinosis or cancer* or carcinoma* or adenocarcinoma* or neoplasm* or neoplastic or metasta* or malignan* or tumour or tumours or tumor or tumors or (cardiac adj3 (arrest or arrhythmia* or disease* or disorder* or surg* or syndrom*)) or cardiomyopath* or ((cardiovascular or coronary) adj3 (disease* or disorder* or event* or syndrom*)) or cachexia or ((cecal or colon* or duoden*) adj3 (disease* or disorder* or syndrom*)) or cerebral palsy or (cerebro* adj3 (degenerat* or disease* or disorder* or event* or syndrom*)) or chronic obstructive disease* or cirrhosis or claudication or colic or copd or ((coordination or co-ordination) adj3 (impair* or lack)) or congenital abnormalit* or (congential adj3 (disease* or disorder* or syndrom*)) or ((connective or collagen* or skin) adj3 (disease* or disorder* or syndrom*)) or collagenous sprue or colitis or colotides or coxarthrosis or crohn* or cushing* or cyanosis or cystic fibrosis or cystitis or deaf* or deformit* or delayed puberty or dental or depressive disorder* or dysphoria or dysthymia or disabled or (physical adj3 (deform* or disab* or impair*)) or dermatitis or dermato* or dorsopath* or diabet* or down* syndrom* or duhring* or duehring* or duhrig* or duehrig* or dyscoordination or dys-coordination or dys-coordination or dys-co-ordination or dysentery or dyssynergia or dys-synergia or emaciat* or enteritis or enterocolitis* or enteropathy or dystonia or eczema or edema or oedema or elfin face sydrom* or encephalopath* or (endocrine adj3 (disease* or disorder* or syndrom*)) or enuresis or enteropath* or epilep* or (eye adj3 (disease* or disorder* or manifestation* or syndrom*)) or fatigue syndrome or chronic fatigue or failure to thrive or fibromyalgia or fibrosis or food hypersensitivity or fractures or gammaglobulinemia or gammaglobulinaemia or (gardner* adj3 (syndrom* or disease* or disorder*)) or gastritis or gastroenteritis or gout or (glomerul* adj3 (disease* or disorder* or syndrom*)) or (gonodal adj3 dysgen*) or (growth adj3 (disease* or disorder* or syndrom*)) or headache* or

For peer review only

((hemic or haemic or lymph*) adj3 (disease* or disorder* or syndrom*)) or hematuria or haematuria or hemophili* or haemophili* or ((hearing or visual or vision or sight) adj3 (aid* or impair* or loss)) or (heart adj3 (disease* or disorder* or syndrom* or manifestation* or myopath*)) or hemiplegi* or hepatitis or hemodialysis or haemodialysis or (heart adj3 (disease* or disorder* or failure or syndrom*)) or hiv or human immunodeficiency virus or acquired immunodeficieny syndrom* or heerfordt waldenstrom* or hidroa or hydroa or hippel lindau* or hypertensi* or hypotensi* or hyperthyroidism or hypothyroidism or hypocortisolism or hypocorticism or hypoadrenalism or hyperthyroidism or hypothyroidism or incoordination or inco-ordination or in-coordination or in-co-ordination or infertility or subfertility or sub-fertility or sterility or inflammatory disease* or inflammatory bowel disease* or incontinen* or intestinal atresi* or intussusception or irritable bowel or ischemi* or ischaemi* or fistula* or (joint adj3 (disease* or disorder* or syndrom*)) or (jejunal adj3 (disease* or disorder* or syndrom*)) or kyphosis or lav-htlv* or leukemia or leukaemia or ((liver or hepatic) adj3 (disease* or disorder* or failure or syndrom*)) or lordosis or (lung adj3 (disease* or disorder*)) or lupus or lymphoma or lymphogranuloma* or lymphadenopath* or lymphotrop* or machado joseph* or macular degeneration or malnutrition or (mental adj2 (disorder* or disease* or health or illness*)) or mania or manic or (menstr* adj3 (ceas* or disturb* or disease* or disorder* or stop* or syndrom*)) or migraine* or (mitochondrial adj3 (disease* or disorder* or syndrom*)) or movement disorder* or mucinosis or musculoskeletal or narp syndrom* or necrotizing or nephrotic* or neuromuscular or non-hodgkin* or nonhodgkin* or multiple sclerosis or myeloma or myocarditis or myocardiopath* or myopath* or (myocardi* adj3 (deteri* or disease* or disorder* or syndrom* or manifestation*)) or nephrotic syndrome* or ((neurodegenerat* or neuro-degenerat) adj3 (disease* or disorder* or syndrom*)) or ((nutritional or metabolic) adj3 (disease* or disorder* or syndrom*)) or ((organ* or kidney or stem cell) adj3 (transplant* or recipient*)) or (nervous system adj3 (disease* or disorder* or syndrom*)) or (neurological adj3 (condition* or disease* or disorder* or syndrom*)) or neuropath* or neurosarcoido* or occlusion* or obesity or obese or orthopedic* or orthopaedic* or ((esophageal or oesophageal) adj3 (disease* or disorder* or syndrom*)) or ((oral or mouth) adj3 (disease* or disorder* or syndrom* or manifestation*)) or osteo* or otitis media or otorhinolaryngolog* or otosclerosis or (pancrea* adj3 (disease* or disorder* or syndrom*)) or papulosquamous or paraplegi* or parkinson* or (peripheral adj3 (arterial or vascular or disease* or disorder* or syndrom*)) or (peritoneal adj3 (disease* or disorder* or syndrom*)) or photodermato* or pick disease* or pneumo* or polio* or polyp* or polydipsia or polyarthropath* or polyarteritis or polyarthrosis or polyneuropath* or porphyrias or (pregnancy adj3 complicat*) or premature aging or proteostasis or pseudophakia or psoriasis or parapsoriasis or prolapse or (pulmonary adj3 (disease* or disorder* or syndrom*)) or purpur* or (recurren* adj3 (abortion* or miscarr*)) or (rect* adj3 (disease* or disorder* or syndrom*)) or ((renal or kidney) adj3 (disease* or disorder* or failure or syndrom*)) or (respiratory adj3 (disease* or disorder* or syndrom*)) or reticulocytosis or retinopathy or rheumat* or ricket* or sarcoido* or sepsis or septic* or seizure* or sclerosis or scoliosis or sickle cell or ((sjogren* or sjoegren* or sicca*) adj3 (syndrom* or disease* or disorder*)) or ((skin or connective tissue) adj3 (disease* or disorder* or syndrom*)) or sleep disorder* or sleep apnea or sleep apnoea or insomnia* or dyssomnia* or hypersomnia* or spina bifida or muscular atropy or short stature* or short bowel syndrom* or (spin* adj3 (degenerat* or disease* or disorder* or syndrom*)) or (spleen* adj3 (disease* or disorder* or syndrom*)) or spondylo* or stenosis* or stoma* or (stomach adj3 (disease* or disorder* or syndrom*)) or stroke or strokes or cerebral infarct* or tetraplegi* or thyroiditis* or (thyroid* adj3 (disease* or disorder* or dysfunction* or syndrom*)) or ((tooth or teeth or enamel) adj3 (decay* or discolo* or disease* or disorder* or dysfunction* or syndrom*)) or tropical sprue or tuberculosis or (systemic adj3 (disorder* or disease* or syndrom*)) or thrombocyto* or tremor or tremors or (turner* adj3 (syndrom* or disease* or disorder*)) or ulcer* or (urogenital adj3 (disease*

For peer review only

or disorder* or syndrom*)) or vasculopath* or (vascular adj3 (disease* or disorder* or occlu* or syndrom*)) or vestibular or ((virus or viral or bacteri* or parasit*) adj3 (infection* or disease*)) or (wasting adj3 (disorder* or disease* or syndrom*)) or (whipple adj3 (disorder* or disease* or syndrom*)) or (william* adj3 (syndrom* or disease* or disorder*)) or zoster*).ti,ab,kw. (14232139)

17 ((digestive system* or duoden* or gastr* or intestin* or nutrition* or malabsor* or metabolic*) adj3 (disease* or disorder* or syndrom* or manifestation*)).ti,ab,kw. (254303)

18 (constipation or constipated or diarrhoea or diarrhea or (abdominal adj3 (disten* or pain or bloating or cramp or cramps)) or flatulence or meteorism or steatorrhoea or steatorrhea or ((acid base or calcium or cyanocobalamin* or electrolyte* or folate or folic acid or glucose or IgA or immunoglobulin A or iron or lactose or lipid* or phosph* or protein* or triglyceride* or vitamin* or mineral*) adj3 (disorder* or deficien* or imbalance* or insufficiency or intoleran*)) or fever* or febrile or malaise or fatigue or letharg* or exhaustion or vomiting or nausea or emesis or sickness or weight loss or wait gain or hot flashes or hot flushes or medically unexplained or unexplained symptoms or unexplained medical or (signs adj2 symptoms) or sleepiness or ((calciferol or cholecalciferol or colecalciferol or egocalciferol) adj3 (disorder* or deficien* or imbalance* or insufficiency or intoleran*)) or conditions linked or linked conditions).ti,ab,kw. (1126673)

19 (medical* morbid* or (medical* adj3 comorbid*) or (medical* adj3 co-morbid*) or multimorbid* or multi* morbid* or multi* comorbid* or multi* co-morbid* or multi* physical*).ti,ab,kw. (30923)

20 first-degree relative/ (6249)

21 ((first adj5 (relative* or relation?)) and c?eliac).ti,ab,kw. (452)

22 or/13-21 (18446309)

23 (((case? adj2 find*) or detect* or diagnos* or predict* or incidence or prevalence or seroprevalence* or risk or risk factor? or risk indicator? or screen* or test*) adj3 c?eliac*).ti,ab,kw. (7675)

24 (c?eliac and ((detect* or diagnos* or predict* or incidence or prevalence or seroprevalence* or risk or risk factor? or screen* or test*) adj2 CD)).ab. (3440)

25 (((association? or associated or indicator? or indicated or predict*) adj3 (risk? or symptom* or disease? or disorder? or histor*)) and c?eliac).ti,ab,kw. (4503)

26 ((association between or relationship between) and c?eliac?).ti,ab,kw. (2279)

27 *celiac disease/di [Diagnosis] (5931)

28 celiac disease/ and (*diagnosis/ or *differential diagnosis/ or *seroprevalence/ or *screening/ or *screening test/ or *incidence/ or *prevalence/) (1064)

29 celiac disease/ and ("prediction and forecasting"/ or prediction/ or predictive validity/ or predictive value/) (755)

30 or/23-29 (16111)

31 5 and 22 and 30 (15090)

32 (((double* or dual*) adj5 diagnos* adj5 c?eliac?) or (comorbid* adj5 c?eliac)).ti,ab,kw. (85)

33 (high risk adj5 c?eliac?).ti,ab,kw. (103)

34 (famil* histor* adj2 c?eliac).ti,ab,kw. (93)

35 (children adj of* adj5 c?eliac).ti,ab,kw. (30)

36 celiac disease test kit/ (11)

37 or/32-36 (315)

38 12 or 31 or 37 (17603)

39 limit 38 to embase status (11243)

40 ((celiac* or coeliac*) adj2 (asymptomatic or atypical or adolesc* or adult* or cases or cohort* or child* or disease* or family or families or familial or genetic predispos* or genetic pre-dispos* or heredit* or infant* or men or patient* or people or population* or subjects or symptomatic or syndrom* or sprue or women)).ti,ab,kw. (26735)

41 limit 40 to (article-in-press status or in-process status) (372)

42 c?eliac.ti. (20558)

43 limit 38 to conference abstract status (4569)

44 42 and 43 (2794)

45 39 or 41 or 44 (14409)

46 ((celiac* or coeliac*) adj (angiograp* or arter* or axis or plexus or trunk)).ti,ab,kw,hw. (12186)

47 case report.ti. (283693)

48 ((case adj of*) and (patient or man or woman)).ti,ab. and case report.sh. (347041)

49 (editorial or letter).pt. (1747770)

50 (exp experimental organism/ or animal tissue/ or animal cell/ or exp animal disease/ or exp carnivore disease/ or exp bird/ or exp experimental animal welfare/ or exp animal husbandry/ or animal behavior/ or exp animal cell culture/ or exp marine species/ or nonhuman/ or animal.hw.) not human/ (6072159)

51 ((rat or rats or mouse or mice or rodent* or animal* or murine or porcine or feline or canine or dog or dogs or cat or cats or pig or pigs or monkey* or macaque*) not human*).ti. (1957387)

52 or/46-51 (8620748)

53 45 not 52 (12083)

54 case control study/ or hospital based case control study/ or population based case control study/ or exp longitudinal study/ or prospective study/ or retrospective study/ (1667885)

55 case finding/ or cohort analysis/ or control group/ or correlational study/ or cross-sectional study/ (1006525)

56 seroepidemiology/ (3934)

57 (cohort or case control* or case finding or cross-sectional or longitudinal).ti,ab,kw. (1661606)

For peer review only

58 study.ti. (1569542)

59 (study and (analys* or cases or clinical or control* or compar* or correlat* or associat* or epidemiolog* or evaluat* or examin* or investigat* or observ* or population based or prospectiv* or retrospectiv* or serolog*)).ti,ab,kw. (8983424)

60 (case? and control*).ab. (657067)

61 (controls or (control adj (group? or participants or patients))).ab. (1656326)

62 (groups or subgroup? or sub-group?).ab. (3028674)

63 (group? adj5 (analys* or compar* or control* or correlat* or associat*)).ab. (1489755)

64 exp regression analysis/ (434324)

65 statistical model/ (158724)

66 ((analys* or logistic*) adj1 (model* or regression*)).ab. (661228)

67 study.ab. /freq=2 (3165201)

68 or/54-67 (12212707)

69 53 and 68 (7566)

70 limit 69 to yr="1997 -Current" (7032)

****

Database: Ovid MEDLINE(R) and Epub Ahead of Print, In-Process & Other Non-Indexed Citations and Daily <1946 to February 05, 2020>

Search Strategy: ARC-Coeliac-MEDLINE-Final

----

1 Celiac Disease/ (19693)

2 c?eliac.ti,ab,kf. (27278)

3 or/1-2 (32722)

4 ((detect* or diagnos* or identif* or decision* or predict* or prognos* or risk or risks or stratif* or validat*) adj3 (model* or factor* or algorithm? or scor* or system or technique? or aid? or rule or rules or index or variable* or tool? or panel or criteri* or characteristic? or history or finding* or value* or assay or stratif* or biomarker?)).ti,ab,kf. (1978188)

5 ((detect* or diagnos* or identif* or decision* or predict* or prognos* or stratif* or validat*) adj3 (risk or risks or model* or factor* or algorithm? or scor* or system or technique? or aid? or rule or rules or index or variable* or tool? or panel or criteri* or characteristic? or history or finding* or value* or assay or stratif* or biomarker?)).ti,ab,kf. (1547617)

For peer review only

6 (clinic* adj3 (model* or algorithm? or scor* or risk or risks or rule or rules or predict* or index or tool? or panel or criteri* or decision* or stratif* or diagnos* or detect* or identif* or probabilit*)).ti,ab,kf. (420165)

7 ((multivaria* or multicomponent?) adj3 (model* or algorithm? or scor* or rule or rules or index or tool? or panel or criteri*)).ti,ab,kf. (69716)

8 (clinical decision making or (decision and logistic models)).sh. (7874)

9 or/4-8 (2337727)

10 3 and 9 (3401)

11 Risk Factors/ or Disease Predisposition/ or Genetic Predisposition to Disease/ (907433)

12 exp "bacterial infections and mycoses"/ or exp virus diseases/ or exp parasitic diseases/ or exp neoplasms/ or exp musculoskeletal diseases/ or exp stomatognathic diseases/ or exp respiratory tract diseases/ or exp otorhinolaryngologic diseases/ or exp nervous system diseases/ or exp eye diseases/ or exp male urogenital diseases/ or exp "female urogenital diseases and pregnancy complications"/ or exp cardiovascular diseases/ or exp "hemic and lymphatic diseases"/ or exp "congenital, hereditary, and neonatal diseases and abnormalities"/ or exp "skin and connective tissue diseases"/ or exp endocrine system diseases/ or exp immune system diseases/ or exp "disorders of environmental origin"/ or disease/ or digestive system diseases/ or exp biliary tract diseases/ or exp digestive system abnormalities/ or exp digestive system fistula/ or exp digestive system neoplasms/ or exp liver diseases/ or exp pancreatic diseases/ or exp peritoneal diseases/ or gastrointestinal diseases/ or exp esophageal diseases/ or exp gastroenteritis/ or exp gastrointestinal hemorrhage/ or exp gastrointestinal neoplasms/ or intestinal diseases/ or exp cecal diseases/ or exp colonic diseases/ or exp duodenal diseases/ or exp dysentery/ or exp enteritis/ or exp enterocolitis/ or exp hiv enteropathy/ or exp ileal diseases/ or exp inflammatory bowel diseases/ or exp intestinal atresia/ or exp intestinal diseases, parasitic/ or exp intestinal fistula/ or exp intestinal neoplasms/ or exp intestinal obstruction/ or exp intestinal perforation/ or exp intestinal polyposis/ or exp jejunal diseases/ or malabsorption syndromes/ or blind loop syndrome/ or collagenous sprue/ or lactose intolerance/ or short bowel syndrome/ or sprue, tropical/ or steatorrhea/ or whipple disease/ or mesenteric ischemia/ or mesenteric vascular occlusion/ or pneumatosis cystoides intestinalis/ or protein-losing enteropathies/ or exp rectal diseases/ or zollinger-ellison syndrome/ or exp stomach diseases/ or exp tuberculosis, gastrointestinal/ or exp visceral prolapse/ or exp liver diseases/ or exp pancreatic diseases/ or exp peritoneal diseases/ or "nutritional and metabolic diseases"/ or metabolic diseases/ or exp acid-base imbalance/ or exp bone diseases, metabolic/ or exp brain diseases, metabolic/ or exp calcium metabolism disorders/ or exp dna repair-deficiency disorders/ or exp glucose metabolism disorders/ or exp hyperlactatemia/ or exp iron metabolism disorders/ or exp lipid metabolism disorders/ or malabsorption syndromes/ or exp blind loop syndrome/ or exp collagenous sprue/ or exp hyperhomocysteinemia/ or exp lactose intolerance/ or exp sprue, tropical/ or exp steatorrhea/ or exp whipple disease/ or exp metabolic syndrome/ or exp metabolism, inborn errors/ or exp mitochondrial diseases/ or exp phosphorus metabolism disorders/ or exp porphyrias/ or exp proteostasis deficiencies/ or exp skin diseases, metabolic/ or exp wasting syndrome/ or exp water-electrolyte imbalance/ or exp nutrition disorders/ or "SIGNS and SYMPTOMS"/ or exp aging, premature/ or exp asthenia/ or exp body temperature changes/ or exp body weight/ or exp cardiac output, high/ or exp cardiac output, low/ or exp chills/ or exp cyanosis/ or exp edema/ or exp eye manifestations/ or exp failure to thrive/ or exp fatigue/ or exp feminization/ or exp fetal distress/ or exp heart murmurs/ or exp hot flashes/ or exp

For peer review only

hypergammaglobulinemia/ or exp hyperlactatemia/ or exp hypertriglyceridemic waist/ or exp intermittent claudication/ or exp medically unexplained symptoms/ or exp mobility limitation/ or exp motion sickness/ or exp myocardial stunning/ or exp neurologic manifestations/ or exp oral manifestations/ or exp polydipsia/ or exp prodromal symptoms/ or exp pseudophakia/ or exp renal colic/ or exp reticulocytosis/ or exp "signs and symptoms, digestive"/ or exp "signs and symptoms, respiratory"/ or exp skin manifestations/ or exp sleepiness/ or exp travel-related illness/ or exp urological manifestations/ or exp virilism/ (13521118)

13 exp "anatomy (non mesh)"/ab, pa [abnormalities, pathology] (1686610)

14 ((addison* adj3 (disease* or disorder* or syndrom*)) or alopecia or baldness or amenorrhea* or amenorrhoea* or oligomenorrhea* or oligomenorrhoea* or anxiety disorder* or anorexia or adrenal insufficienc* or adrenocortical insufficienc* or allerg* or anemi* or anaemi* or angina or aneurysm or ankylosing spondylitis or arthropath* or arthriti* or arthrosis or arthroses or asthma* or asthenia or ataxia or atrial fibrillation or atrophy or autoimmune disorder* or (autoimmune adj (disease* or disorder* or syndrom*)) or ataxia or avitaminosis or bonnevie ullrich or back pain or (biliary adj3 (disease* or disorder* or syndrom*)) or (bipolar adj3 disorder*) or blindness or blind loop syndrom* or (blood adj3 poisoning) or brain atroph* or ((bone or bones) adj3 (broken or disease* or disorder* or syndrom*)) or (bone* adj3 (mineral* or densit* or soft* or decay*)) or (brain adj3 (disease* or disorder* or syndrom*)) or (bronchi* adj3 (disease* or disorder* or syndrom*)) or (bowel* adj3 (disease* or disorder* or syndrom*)) or calcinosis or cancer* or carcinoma* or adenocarcinoma* or neoplasm* or neoplastic or metasta* or malignan* or tumour or tumours or tumor or tumors or (cardiac adj3 (arrest or arrhythmia* or disease* or disorder* or surg* or syndrom*)) or cardiomyopath* or ((cardiovascular or coronary) adj3 (disease* or disorder* or event* or syndrom*)) or cachexia or ((cecal or colon* or duoden*) adj3 (disease* or disorder* or syndrom*)) or cerebral palsy or (cerebro* adj3 (degenerat* or disease* or disorder* or event* or syndrom*)) or chronic obstructive disease* or cirrhosis or claudication or colic or copd or ((coordination or co-ordination) adj3 (impair* or lack)) or congenital abnormalit* or (congential adj3 (disease* or disorder* or syndrom*)) or ((connective or collagen* or skin) adj3 (disease* or disorder* or syndrom*)) or collagenous sprue or colitis or colotides or coxarthrosis or crohn* or cushing* or cyanosis or cystic fibrosis or cystitis or deaf* or deformit* or delayed puberty or dental or depressive disorder* or dysphoria or dysthymia or disabled or (physical adj3 (deform* or disab* or impair*)) or dermatitis or dermato* or dorsopath* or diabet* or down* syndrom* or duhring* or duehring* or duhrig* or duehrig* or dyscoordination or dys-coordination or dys-coordination or dys-co-ordination or dysentery or dyssynergia or dys-synergia or emaciat* or enteritis or enterocolitis* or enteropathy or dystonia or eczema or edema or oedema or elfin face sydrom* or encephalopath* or (endocrine adj3 (disease* or disorder* or syndrom*)) or enuresis or enteropath* or epilep* or (eye adj3 (disease* or disorder* or manifestation* or syndrom*)) or fatigue syndrome or chronic fatigue or failure to thrive or fibromyalgia or fibrosis or food hypersensitivity or fractures or gammaglobulinemia or gammaglobulinaemia or (gardner* adj3 (syndrom* or disease* or disorder*)) or gastritis or gastroenteritis or gout or (glomerul* adj3 (disease* or disorder* or syndrom*)) or (gonodal adj3 dysgen*) or (growth adj3 (disease* or disorder* or syndrom*)) or headache* or ((hemic or haemic or lymph*) adj3 (disease* or disorder* or syndrom*)) or hematuria or haematuria or hemophili* or haemophili* or ((hearing or visual or vision or sight) adj3 (aid* or impair* or loss)) or (heart adj3 (disease* or disorder* or syndrom* or manifestation* or myopath*)) or hemiplegi* or hepatitis or hemodialysis or haemodialysis or (heart adj3 (disease* or disorder* or failure or syndrom*)) or hiv or human immunodeficiency virus or acquired immunodeficieny syndrom* or heerfordt waldenstrom* or hidroa or hydroa or hippel lindau* or hypertensi* or hypotensi* or hyperthyroidism or hypothyroidism or hypocortisolism or hypocorticism or hypoadrenalism or

For peer review only

hyperthyroidism or hypothyroidism or incoordination or inco-ordination or in-coordination or in-co-ordination or infertility or subfertility or sub-fertility or sterility or inflammatory disease* or inflammatory bowel disease* or incontinen* or intestinal atresi* or intussusception or irritable bowel or ischemi* or ischaemi* or fistula* or (joint adj3 (disease* or disorder* or syndrom*)) or (jejunal adj3 (disease* or disorder* or syndrom*)) or kyphosis or lav-htlv* or leukemia or leukaemia or ((liver or hepatic) adj3 (disease* or disorder* or failure or syndrom*)) or lordosis or (lung adj3 (disease* or disorder*)) or lupus or lymphoma or lymphogranuloma* or lymphadenopath* or lymphotrop* or machado joseph* or macular degeneration or malnutrition or (mental adj2 (disorder* or disease* or health or illness*)) or mania or manic or (menstr* adj3 (ceas* or disturb* or disease* or disorder* or stop* or syndrom*)) or migraine* or (mitochondrial adj3 (disease* or disorder* or syndrom*)) or movement disorder* or mucinosis or musculoskeletal or narp syndrom* or necrotizing or nephrotic* or neuromuscular or non-hodgkin* or nonhodgkin* or multiple sclerosis or myeloma or myocarditis or myocardiopath* or myopath* or (myocardi* adj3 (deteri* or disease* or disorder* or syndrom* or manifestation*)) or nephrotic syndrome* or ((neurodegenerat* or neuro-degenerat) adj3 (disease* or disorder* or syndrom*)) or ((nutritional or metabolic) adj3 (disease* or disorder* or syndrom*)) or ((organ* or kidney or stem cell) adj3 (transplant* or recipient*)) or (nervous system adj3 (disease* or disorder* or syndrom*)) or (neurological adj3 (condition* or disease* or disorder* or syndrom*)) or neuropath* or neurosarcoido* or occlusion* or obesity or obese or orthopedic* or orthopaedic* or ((esophageal or oesophageal) adj3 (disease* or disorder* or syndrom*)) or ((oral or mouth) adj3 (disease* or disorder* or syndrom* or manifestation*)) or osteo* or otitis media or otorhinolaryngolog* or otosclerosis or (pancrea* adj3 (disease* or disorder* or syndrom*)) or papulosquamous or paraplegi* or parkinson* or (peripheral adj3 (arterial or vascular or disease* or disorder* or syndrom*)) or (peritoneal adj3 (disease* or disorder* or syndrom*)) or photodermato* or pick disease* or pneumo* or polio* or polyp* or polydipsia or polyarthropath* or polyarteritis or polyarthrosis or polyneuropath* or porphyrias or (pregnancy adj3 complicat*) or premature aging or proteostasis or pseudophakia or psoriasis or parapsoriasis or prolapse or (pulmonary adj3 (disease* or disorder* or syndrom*)) or purpur* or (recurren* adj3 (abortion* or miscarr*)) or (rect* adj3 (disease* or disorder* or syndrom*)) or ((renal or kidney) adj3 (disease* or disorder* or failure or syndrom*)) or (respiratory adj3 (disease* or disorder* or syndrom*)) or reticulocytosis or retinopathy or rheumat* or ricket* or sarcoido* or sepsis or septic* or seizure* or sclerosis or scoliosis or sickle cell or ((sjogren* or sjoegren* or sicca*) adj3 (syndrom* or disease* or disorder*)) or ((skin or connective tissue) adj3 (disease* or disorder* or syndrom*)) or sleep disorder* or sleep apnea or sleep apnoea or insomnia* or dyssomnia* or hypersomnia* or spina bifida or muscular atropy or short stature* or short bowel syndrom* or (spin* adj3 (degenerat* or disease* or disorder* or syndrom*)) or (spleen* adj3 (disease* or disorder* or syndrom*)) or spondylo* or stenosis* or stoma* or (stomach adj3 (disease* or disorder* or syndrom*)) or stroke or strokes or cerebral infarct* or tetraplegi* or thyroiditis* or (thyroid* adj3 (disease* or disorder* or dysfunction* or syndrom*)) or ((tooth or teeth or enamel) adj3 (decay* or discolo* or disease* or disorder* or dysfunction* or syndrom*)) or tropical sprue or tuberculosis or (systemic adj3 (disorder* or disease* or syndrom*)) or thrombocyto* or tremor or tremors or (turner* adj3 (syndrom* or disease* or disorder*)) or ulcer* or (urogenital adj3 (disease* or disorder* or syndrom*)) or vasculopath* or (vascular adj3 (disease* or disorder* or occlu* or syndrom*)) or vestibular or ((virus or viral or bacteri* or parasit*) adj3 (infection* or disease*)) or (wasting adj3 (disorder* or disease* or syndrom*)) or (whipple adj3 (disorder* or disease* or syndrom*)) or (william* adj3 (syndrom* or disease* or disorder*)) or zoster*).ti,ab,kf. (11316008)

15 ((digestive system* or duoden* or gastr* or intestin* or nutrition* or malabsor* or metabolic*) adj3 (disease* or disorder* or syndrom* or manifestation*)).ti,ab,kf. (180683)

16 (constipation or constipated or diarrhoea or diarrhea or (abdominal adj3 (disten* or pain or bloating or cramp or cramps)) or flatulence or meteorism or steatorrhoea or steatorrhea or ((acid base or calcium or cyanocobalamin* or electrolyte* or folate or folic acid or glucose or IgA or immunoglobulin A or iron or lactose or lipid* or phosph* or protein* or triglyceride* or vitamin* or mineral*) adj3 (disorder* or deficien* or imbalance* or insufficiency or intoleran*)) or fever* or febrile or malaise or fatigue or letharg* or exhaustion or vomiting or nausea or emesis or sickness or weight loss or wait gain or hot flashes or hot flushes or medically unexplained or unexplained symptoms or unexplained medical or (signs adj2 symptoms) or sleepiness or ((calciferol or cholecalciferol or colecalciferol or egocalciferol) adj3 (disorder* or deficien* or imbalance* or insufficiency or intoleran*)) or conditions linked or linked conditions).ti,ab,kf. (805001)

17 (medical* morbid* or (medical* adj3 comorbid*) or (medical* adj3 co-morbid*) or multimorbid* or multi* morbid* or multi* comorbid* or multi* co-morbid* or multi* physical*).ti,ab,kf. (18919)

18 (first-degree relative or family or family relation or family history or mother or father or parent or daughter or son).sh. or (brother or sister or sibling).hw. (83159)

19 (first adj5 (relative* or relation?)).ti,ab,kf. (21485)

20 (children adj of*).ti,ab,kf. (32315)

21 or/11-20 (16944516)

22 (((case? adj2 find*) or detect* or diagnos* or predict* or incidence or prevalence or seroprevalence* or risk or risk factor? or risk indicator? or screen* or test*) adj3 c?eliac*).ti,ab,kf. (4839)

23 (c?eliac and ((detect* or diagnos* or predict* or incidence or prevalence or seroprevalence* or risk or risk factor? or screen* or test*) adj2 CD)).ab. (1782)

24 (((association? or associated or indicator? or indicated or predict*) adj3 (risk? or symptom* or disease? or disorder? or histor*)) and c?eliac).ti,ab,kf. (2819)

25 ((association between or relationship between) and c?eliac?).ti,ab,kf. (1328)

26 celiac disease/di [Diagnosis] (5652)

27 celiac disease/ and (diagnosis/ or diagnosis, differential/ or seroprevalence/ or seroepidemiologic studies/ or screening/ or prediction/ or prevalence/ or incidence/) (2995)

28 or/22-27 (11534)

29 3 and 21 and 28 (9724)

30 Celiac Disease/di and (atypical or extraintestinal or extra-intestinal or nonspecific or non-specific).mp. (490)

31 (((double* or dual*) adj5 diagnos* adj5 c?eliac?) or (comorbid* adj5 c?eliac)).ti,ab,kf. (42)

32 (high risk adj5 c?eliac?).ti,ab,kf. (67)

33 (famil* histor* adj2 c?eliac).ti,ab,kf. (33)

34 (children adj of* adj5 c?eliac).ti,ab,kw. (19)

For peer review only

35 or/30-34 (642)

36 10 or 29 or 35 (11362)

37 ((celiac* or coeliac*) adj2 (asymptomatic or atypical or adolesc* or adult* or cases or cohort* or child* or disease* or family or families or familial or genetic predispos* or genetic pre-dispos* or heredit* or infant* or men or patient* or people or population* or subjects or symptomatic or syndrom* or sprue or women)).ti,ab,kf. (18800)

38 limit 37 to ("in data review" or in process or publisher) (605)

39 36 or 38 (11704)

40 ((celiac* or coeliac*) adj (angiograp* or arter* or axis or plexus or trunk)).ti,ab,kf,hw. (8461)

41 case report.ti. (230986)

42 ((case adj of*) and (patient or man or woman)).ti,ab. and case reports.pt. (252275)

43 (comment or editorial or letter or newspaper article).pt. (1825467)

44 (exp Animals/ or exp Animal Experimentation/ or exp Models, Animal/ or exp Animals, Laboratory/ or exp rodentia/) not Humans/ (4673001)

45 ((rat or rats or mouse or mice or rodent* or animal* or murine or porcine or feline or canine or dog or dogs or cat or cats or pig or pigs or monkey* or macaque*) not human*).ti. (1856683)

46 or/40-45 (7137365)

47 39 not 46 (9645)

48 exp epidemiologic studies/ (2435409)

49 (cohort or case control* or case finding or cross-sectional or longitudinal).ti,ab,kf. (1114977)

50 study.ti. (1330964)

51 (study and (analys* or cases or clinical or control* or compar* or correlat* or associat* or epidemiolog* or evaluat* or examin* or investigat* or observ* or population based or prospectiv* or retrospectiv* or serolog*)).ti,ab,kf. (6665955)

52 (case? and control*).ab. (448997)

53 (controls or (control adj (group? or participants or patients))).ab. (1189049)

54 (groups or subgroup? or sub-group?).ab. (2170205)

55 (group? adj5 (analys* or compar* or control* or correlat* or associat*)).ab. (1004351)

56 exp Regression Analysis/ (416341)

57 statistical model/ (89771)

58 ((analys* or logistic*) adj1 (model* or regression*)).ab. (456097)

59 study.ab. /freq=2 (2157950)

60 or/48-59 (9618005)

For peer review only

61 47 and 60 (5419)

62 limit 61 to yr="1997 -Current" (4786)

****

Cochrane Library, Issue 2 of 12, 2020

#1 MeSH descriptor: [Celiac Disease] this term only [63]

#2 ("celiac disease" or "coeliac disease"): kw [338]

#3 ((celiac* or coeliac*) near/3 (asymptomatic or atypical or adolesc* or adult* or cases or cohort* or child* or disease* or family or families or familial or genetic predispos* or genetic pre-dispos* or heredit* or infant* or men or patient* or people or population* or subjects or symptomatic or syndrom* or sprue or women)):ti,ab [782]

#4 (#1 or #2 or #3) [886]

#5 ((celiac* or coeliac*) next (angiograp* or arter* or axis or plexus or trunk)):ti,ab,kw [276]

#6 #4 not #5 (n=870) [870]

****

Web of Science: Science Citation Index Expanded (SCI-EXPANDED), SCI-EXPANDED, Conference Proceedings Citation Index- Science SSCI

[Limit to celiac or coeliac in title/abstract, n=6118]

#28 (#26 not #27) [7,430]

#27 (TI=("case report" or "a case of" or "a rare case of")) or (TS=(("a case of" or "a rare case of") near (patient or man or woman or child or adolescent or "an adult"))) [202,526]

#26 (#25 AND #24) [7493]
Indexes=SCI-EXPANDED, CPCI-S Timespan=1997-2020

#25 ((TS=(seroepidemiolog* or cohort or "case control*" or "case find*" or cross-sectional or longitudinal or "population based" or prospective* or retrospective*) or TI=(study) or TS=(study and (analys* or cases or clinical or control* or compar* or correlat* or associat* or epidemiolog* or evaluat* or examin* or investigat* or observ* or population based or prospectiv* or retrospectiv* or serolog*)) or TS=((case or cases) and control*) or TS=(controls or "control* group*" or "control* participant*" or "control* patient*" or groups or subgroup* or sub-group*) or TS=(group* near (analys* or compar* or control* or correlat* or associat*)) or TS=("regression analysis" or "regression model*" or "statistical model*" or "logistic* model*")) NOT (TI=(rat or rats or mouse or mice or rodent* or animal* or murine or porcine or feline or canine or dog or dogs or cat or cats or pig or pigs or monkey* or macaque* or nonhuman or "non human"))) [19,956,721
Indexes=SCI-EXPANDED, SSCI, A&HCI, CPCI-S, CPCI-SSH, ESCI Timespan=1900-2020

#24 (#23 OR #22 OR #21 OR #8) [13,272]

For peer review only

#23 (TS=((“high risk” or “famil* histor*” or “child* of”) near (celiac or coeliac)) or TS=(”celiac disease test kit”)) [233]
Indexes=SCI-EXPANDED, SSCI, A&HCI, CPCI-S, CPCI-SSH, ESCI Timespan=1900-2020

#22 (TS=((double* or dual*) near diagnos* near (celiac or coeliac))) or (TS=(comorbid* near (celiac or coeliac))) [111]
Indexes=SCI-EXPANDED, SSCI, A&HCI, CPCI-S, CPCI-SSH, ESCI Timespan=1900-2020

#21 (#20 AND #15 AND #1) [12,140]

#20 (#19 OR #18 OR #17 OR #16) [18,586]

#19 (TS=((“association* between” or “relationship between”) and (celiac or coeliac))) [1,458]
Indexes=SCI-EXPANDED, SSCI, A&HCI, CPCI-S, CPCI-SSH, ESCI Timespan=1900-2020

#18 ((TS=((association* or associated or indicator* or indicated or predict*) near (risk* or symptom* or disease* or disorder* or histor*))) and (TS=(celiac or coeliac))) [5,833]
Indexes=SCI-EXPANDED, SSCI, A&HCI, CPCI-S, CPCI-SSH, ESCI Timespan=1900-2020

#17 (TS=((celiac or coeliac) and ("detect* CD" or “CD detect*” or "detect* of CD" or "detect* with CD" or “diagnos* CD” or "CD diagnos*" or “diagnos* of CD” or "diagnos* with CD" or “predict* CD” or "CD predict*" or “predict* of CD” or "predict* with CD" or “inciden* CD” or "CD inciden*" or “inciden* of CD” or "inciden* with CD" or “prevalen* CD” or "CD prevalen*" or “prevalen* of CD” or " prevalen* with CD" or “seroprevalen* CD” or "CD seroprevalen*" or “seroprevalen* of CD” or "seroprevalen* with CD" or "CD risk*" or “risk* of CD” or “risk* for CD” or “risk* factor* of CD” or “risk factor* for CD” or “CD screen*” or "screen* for CD" or “CD test*” or "test* for CD"))) [1,489]
Indexes=SCI-EXPANDED, SSCI, A&HCI, CPCI-S, CPCI-SSH, ESCI Timespan=1900-2020

#16 (TS=(("case finding" or detect* or diagnos* or predict* or incidence or prevalence or seroprevalence* or risk or "risk factor*" or "risk indicator*" or screen* or test*) and (celiac* or coeliac*))) [17,056]
Indexes=SCI-EXPANDED, SSCI, A&HCI, CPCI-S, CPCI-SSH, ESCI Timespan=1900-2020

#15 (#14 OR #13 OR #12 OR #11 OR #10 OR #9) [14,174,134]

#14 (TI=(manifestation*)) [51,353]
Indexes=SCI-EXPANDED, SSCI, A&HCI, CPCI-S, CPCI-SSH, ESCI Timespan=1900-2020

#13 (TS=("first degree" near (related or relative* or relation*))) [11,114]
Indexes=SCI-EXPANDED, SSCI, A&HCI, CPCI-S, CPCI-SSH, ESCI Timespan=1900-2020

#12 (TS=("medical* morbid*" or (medical* near (comorbid* or co-morbid*)) or multimorbid* or "multi* morbid*" or "multi* comorbid*" or "multi* co-morbid*" or "multi* physical*")) [25,013]
Indexes=SCI-EXPANDED, SSCI, A&HCI, CPCI-S, CPCI-SSH, ESCI Timespan=1900-2020

#11 ((TS=(constipation or constipated or diarrhoea or diarrhea or (abdominal near (disten* or pain or bloating or cramp or cramps)) or flatulence or meteorism or steatorrhoea or steatorrhea)) or (TS=((“acid base” or calcium or cyanocobalamin* or electrolyte* or folate or "folic acid"or glucose or IgA or "immunoglobulin A" or iron or lactose or lipid* or phosph* or protein* or triglyceride* or vitamin* or mineral*) near (disorder* or deficien* or imbalance* or insufficiency or intoleran*))) or (TS=(fever* or febrile or malaise or fatigue or letharg* or exhaustion or vomiting or nausea or emesis or sickness or “weight loss” or “wait gain” or “hot flashes” or “hot flushes” or “medically unexplained” or “unexplained medical” or “unexplained symptom*” or “signs and symptoms” or

For peer review only

“condition* linked” or “linked condition*” or “condition* associated with” or sleepiness)) or (TS=((calciferol or cholecalciferol or colecalciferol or egocalciferol) near (disorder* or deficien* or imbalance* or insufficiency or intoleran*)))) or (TS=((anxiety or depression or mood) near (celiac or coeliac))) [1,055,350]
Indexes=SCI-EXPANDED, SSCI, A&HCI, CPCI-S, CPCI-SSH, ESCI Timespan=1900-2020

#10 (TS=("digestive system* disease*" or "digestive system* disorder*" or "digestive system* syndrom*" or "digestive system manifestation*" or "duoden* disease*" or "duoden* disorder*" or "duoden* syndrom*" or "duoden* manifestation*" or "gastr* disease*" or "gastr* disorder*" or "gastr* syndrom*" or "gastr* manifestation*" or "intestin* disease*" or "intestin* disorder*" or "intestin* syndrom*" or "intestin* manifestation*" or "malabsor* disease*" or "malabsor* disorder*" or "malabsor* syndrom*" or "malabsor* manifestation*")) [29,189]
Indexes=SCI-EXPANDED, SSCI, A&HCI, CPCI-S, CPCI-SSH, ESCI Timespan=1900-2020

#9 (TS=((addison* near (disease* or disorder* or syndrom*)) or alopecia or baldness or amenorrhea* or amenorrhoea* or oligomenorrhea* or oligomenorrhoea* or "anxiety disorder*" or anorexia or "adrenal insufficienc*" or "adrenocortical insufficienc*" or allerg* or anemi* or anaemi* or angina or aneurysm or "ankylosing spondylitis" or arthropath* or arthriti* or arthrosis or arthroses or asthma* or asthenia or ataxia or "atrial fibrillation" or atrophy or "autoimmune disorders" or "autoimmune diseases" or "autoimmune syndrom*" or ataxia or avitaminosis or "bonnevie ullrich" or "back pain" or (biliary near (disease* or disorder* or syndrom*)) or (bipolar near disorder*) or blindness or "blind loop syndrom*" or (blood near poisoning) or "brain atroph*" or ((bone or bones) near (broken or disease* or disorder* or syndrom*)) or (bone* near (mineral* or densit* or soft* or decay*)) or (brain near (disease* or disorder* or syndrom*)) or (bronchi* near (disease* or disorder* or syndrom*)) or (bowel* near (disease* or disorder* or syndrom*)) or calcinosis or cancer* or carcinoma* or adenocarcinoma* or neoplasm* or neoplastic or metasta* or malignan* or tumour or tumours or tumor or tumors or (cardiac near (arrest or arrhythmia* or disease* or disorder* or surg* or syndrom*)) or cardiomyopath* or ((cardiovascular or coronary) near (disease* or disorder* or event* or syndrom*)) or cachexia or ((cecal or colon* or duoden*) near (disease* or disorder* or syndrom*)) or "cerebral palsy" or (cerebro* near (degenerat* or disease* or disorder* or event* or syndrom*)) or "chronic obstructive disease" or cirrhosis or claudication or colic or COPD or ((coordination or co-ordination) near (impair* or lack)) or "congenital abnormalit*" or (congential near (disease or disorder* or syndrom*)) or ((connective or collagen* or skin) near (disease or disorder* or syndrom*)) or "collagenous sprue" or colitis or colotides or coxarthrosis or crohn* or cushing* or cyanosis or "cystic fibrosis" or cystitis or deaf* or deformit* or "delayed puberty" or dental or "depressive disorder*" or dysphoria or dysthymia or disabled or (physical near (deform* or disab* or impair*)) or dermatitis or dermato* or dorsopath* or diabet* or "down* syndrom*" or duhring* or duehring* or duhrig* or duehrig* or dyscoordination or dys-coordination or dys-coordination or dys-co-ordination or dysentery or dyssynergia or dys-synergia or emaciat* or enteritis or enterocolitis* or dystonia or eczema or edema or oedema or "elfin face sydrom*" or encephalopath* or (endocrine near (disease* or disorder* or syndrom*)) or enuresis or enteropath* or epilep* or (eye near (disease* or disorder* or manifestation* or syndrome*)) or "fatigue syndrome" or "chronic fatigue" or "failure to thrive" or fibromyalgia or fibrosis or "food hypersensitivity" or fractures or gammaglobulinemia or gammaglobulinaemia or (gardner* near (syndrom* or disease* or disorder*)) or gastritis or gastroenteritis or gout or (glomerul* near (disease* or disorder* or syndrom*)) or "gonodal near dysgen*" or (growth near (disease* or disorder* or syndrom*)) or headache* or ((hemic or haemic or lymph*) near (disease* or disorder* or syndrom*)) or hematuria or haematuria or hemophili* or haemophili* or ((hearing or visual or vision or sight) near (aid* or impair* or loss)) or (heart near

For peer review only

(disease* or disorder* or failure or syndrom* or manifestation* or myopathy*)) or hemiplegi* or hepatitis or hemodialysis or haemodialysis hiv or "human immunodeficiency virus" or "acquired immunodeficieny syndrom*" or "heerfordt waldenstrom*" or hidroa or hydroa or "hippel lindau*" or hypertensi* or hypotensi* or hyperthyroidism or hypothyroidism or hypocortisolism or hypocorticism or hypoadrenalism or incoordination or inco-ordination or in-coordination or in-co-ordination or infertility or subfertility or sub-fertil* or sterility or "inflammatory disease*" or "inflammatory bowel disease*" or incontinen* or "intestinal atresi*" or intussusception or "irritable bowel" or ischemi* or ischaemi* or fistula* or (joint near (disease* or disorder* or syndrom*)) or (jejunal near (disease* or disorder* or syndrom*)) or kyphosis or lav-htlv* or leukemia or leukaemia or ((liver or hepatic) near (disease* or disorder* or failure or syndrom*)) or lordosis or (lung near (disease* or disorder*)) or lupus or SLE or lymphoma or lymphogranuloma* or lymphadenopath* or lymphotrop* or "machado joseph*" or "macular degeneration" or malnutrition or (mental near (disorder* or disease* or health or illness*)) or mania or manic or (menstr* near (ceas* or disturb* or disease* or disorder* or stop* or syndrom*)) or migraine* or (mitochondrial near (disease* or disorder* or syndrom*)) or "movement disorder*" or mucinosis or musculoskeletal or "narp syndrom*" or necrotizing or nephrotic syndrom* or neuromuscular or non-hodgkin* or nonhodgkin* or "multiple sclerosis" or myeloma or myocarditis or myocardiopath* or myopath* or (myocardi* near (deteri* or disease* or disorder* or syndrom* or manifestation*)) or "nephrotic syndrome" or ((neurodegenerat* or neuro-degenerat) near (disease* or disorder* or syndrom)) or ((nutritional or metabolic) near (disease* or disorder* or syndrom*)) or ((organ* or kidney or "stem cell") near (transplant* or recipient*)) or ("nervous system" near (disease* or disorder* or syndrom*)) or (neurological near (condition* or disease* or disorder* or syndrom*)) or neuropath* or neurosarcoido* or occlusion* or obesity or obese or orthopedic* or orthopaedic* or ((esophageal or oesophageal) near (disease* or disorder* or syndrom*)) or ((oral or mouth) near (disease* or disorder* or syndrom* or manifestation*)) or osteo* or "otitis media" or otorhinolaryngolog* or otosclerosis or (pancrea* near (disease* or disorder* or syndrom*)) or papulosquamous or paraplegi* or parkinson* or (peripheral near (arterial or vascular or disease* or disorder* or syndrom*)) or (peritoneal near (disease* or disorder* or syndrom*)) or photodermato* or "pick disease*" or pneumo* or polio* or polyp* or polydipsia or polyarthropath* or polyarteritis or polyarthrosis or polyneuropath* or porphyrias or (pregnancy near complicat*) or "premature aging" or proteostasis or pseudophakia or psoriasis or parapsoriasis or prolapse or (pulmonary near (disease* or disorder* or syndrom*)) or purpur* or (recurren* near (abortion* or miscarr*)) or ((rectum or rectal) near (disease* or disorder* or syndrom*)) or ((renal or kidney) near (disease* or disorder* or failure or syndrom*)) or (respiratory near (disease* or disorder* or syndrom*)) or reticulocytosis or retinopathy or rheumat* or ricket* or sarcoido* or sepsis or septic* or seizure* or sclerosis or scoliosis or "sickle cell" or ((sjogren* or sjoegren* or sicca*) near (syndrom* or disease* or disorder*)) or ((skin or "connective tissue") near (disease* or disorder* or syndrom*)) or "sleep disorder*" or "sleep apnea" or "sleep apnoea" or insomnia* or dyssomnia* or hypersomnia* or "spina bifida" or "muscular atropy" or "short stature*" or "short bowel syndrom*" or (spin* near (degenerat* or disease* or disorder* or syndrom*)) or (spleen* near (disease* or disorder* or syndrom*)) or spondylo* or stenosis* or stoma* or (stomach near (disease* or disorder* or syndrom*)) or stroke or strokes or "cerebral infarct*" or tetraplegi* or thyroiditis* or (thyroid* near (disease* or disorder* or dysfunction* or syndrom*)) or ((tooth or teeth or enamel) near (decay* or discolo* or disease* or disorder* or dysfunction* or syndrom*)) or "tropical sprue" or tuberculosis or (systemic near (disorder* or disease* or syndrom*)) or thrombocyto* or tremor or tremors or (turner* near (syndrome* or disease* or disorder*)) or ulcer* or (urogenital near (disease* or disorder* or syndrom*)) or vasculopath* or (vascular near (disease* or disorder* or occlu* or syndrom*)) or vestibular or ((virus or viral or bacteri* or parasit*) near (infection* or disease*)) or

For peer review only

(wasting near (disorder* or disease* or syndrom*)) or (whipple near (disorder* or disease* or syndrom*)) or (william* near (syndrom* or disease* or disorder*)) or zoster*)) [13,693,359]
Indexes=SCI-EXPANDED, SSCI, A&HCI, CPCI-S, CPCI-SSH, ESCI Timespan=1900-2020

#8 (#7 and #1) [5,889]

#7 (#6 OR #5 OR #4 OR #3 OR #2) [6,827,636]

#6 (TS=("clinical decision making" or "logistic model*" or "statistical model*")) [81,189]
Indexes=SCI-EXPANDED, SSCI, A&HCI, CPCI-S, CPCI-SSH, ESCI Timespan=1900-2020

#5 (TS=((multivaria* or multicomponent*) NEAR (model* or algorithm* or score or scores or scoring or rule or rules or index or tool or tools or panel or criteri*))) [156,496]
Indexes=SCI-EXPANDED, SSCI, A&HCI, CPCI-S, CPCI-SSH, ESCI Timespan=1900-2020

#4 (TS=(clinic* NEAR (model* or algorithm* or score or scores or scoring or risk or risks or rule or rules or predict* or index or tool or tools or panel or criteri* or decision* or stratif* or diagnos* or detect* or identif* or probabilit*))) [1,061,406]
Indexes=SCI-EXPANDED, SSCI, A&HCI, CPCI-S, CPCI-SSH, ESCI Timespan=1900-2020

#3 (TS=((detect* or diagnos* or identif* or decision* or predict* or prognos* or stratif* or validat*) NEAR (risk or risks or model* or factor* or algorithm* or score or scores or scoring or system or technique* or aid or aids or rule or rules or index or variable* or tool or tools or panel or criteri* or characteristic* or history or finding* or value* or assay or stratif* or biomarker*))) [5,481,423]
Indexes=SCI-EXPANDED, SSCI, A&HCI, CPCI-S, CPCI-SSH, ESCI Timespan=1900-2020

#2 (TS=((detect* or diagnos* or identif* or decision* or predict* or prognos* or risk or risks or stratif* or validat*) NEAR (model* or factor* or algorithm* or score or scores or scoring or system or technique* or aid or aids or rule or rules or index or variable* or tool or tools or panel or criteri* or characteristic* or history or finding* or value* or assay or stratif* or biomarker*))) [6/133,858]
Indexes=SCI-EXPANDED, SSCI, A&HCI, CPCI-S, CPCI-SSH, ESCI Timespan=1900-2020

#1 TS=((celiac* OR coeliac*) NEAR (asymptomatic OR atypical OR adolesc* OR adult* OR cases OR cohort* OR child* OR disease* OR disorder* OR family OR families OR familial OR "genetic predispos*" OR "genetic pre-dispos*" OR heredit* OR infant* OR men OR patient* OR people OR population* OR subjects OR symptomatic OR syndrom* OR sprue OR women))) NOT (TS=((celiac* OR coeliac*) NEAR (angiography OR artery OR arteries OR axis OR plexus OR trunk))) [25,557]
Indexes=SCI-EXPANDED, SSCI, A&HCI, CPCI-S, CPCI-SSH, ESCI Timespan=1900-2020

For peer review only

Reporting checklist for protocol of a systematic review.

Based on the PRISMA-P guidelines.

Instructions to authors

Complete this checklist by entering the page numbers from your manuscript where readers will find each of the items listed below.

Your article may not currently address all the items on the checklist. Please modify your text to include the missing information. If you are certain that an item does not apply, please write "n/a" and provide a short explanation.

Upload your completed checklist as an extra file when you submit to a journal.

In your methods section, say that you used the PRISMA-Preporting guidelines, and cite them as:

Moher D, Shamseer L, Clarke M, Ghersi D, Liberati A, Petticrew M, Shekelle P, Stewart LA. Preferred Reporting Items for Systematic Review and Meta-Analysis Protocols (PRISMA-P) 2015 statement. Syst Rev. 2015;4(1):1.

		Reporting Item	Page Number
**Title**			
Identification	#1a	Identify the report as a protocol of a systematic review	1

For peer review only

Update	#1b	If the protocol is for an update of a previous systematic review, identify as such	NA
**Registration**			
	#2	If registered, provide the name of the registry (such as PROSPERO) and registration number	1
**Authors**			
Contact	#3a	Provide name, institutional affiliation, e-mail address of all protocol authors; provide physical mailing address of corresponding author	1
Contribution	#3b	Describe contributions of protocol authors and identify the guarantor of the review	11
**Amendments**			
	#4	If the protocol represents an amendment of a previously completed or published protocol, identify as such and list changes; otherwise, state plan for documenting important protocol amendments	6
**Support**			
Sources	#5a	Indicate sources of financial or other support for the review	11
Sponsor	#5b	Provide name for the review funder and / or sponsor	11

For peer review only

Role of sponsor or funder	#5c	Describe roles of funder(s), sponsor(s), and / or institution(s), if any, in developing the protocol	NA
**Introduction**			
Rationale	#6	Describe the rationale for the review in the context of what is already known	4-5
Objectives	#7	Provide an explicit statement of the question(s) the review will address with reference to participants, interventions, comparators, and outcomes (PICO)	5
**Methods**			
Eligibility criteria	#8	Specify the study characteristics (such as PICO, study design, setting, time frame) and report characteristics (such as years considered, language, publication status) to be used as criteria for eligibility for the review	6
Information sources	#9	Describe all intended information sources (such as electronic databases, contact with study authors, trial registers or other grey literature sources) with planned dates of coverage	6
Search strategy	#10	Present draft of search strategy to be used for at least one electronic database, including planned limits, such that it could be repeated	7 and Supplementary material

For peer review only

Study records - data management	#11a	Describe the mechanism(s) that will be used to manage records and data throughout the review	7
Study records - selection process	#11b	State the process that will be used for selecting studies (such as two independent reviewers) through each phase of the review (that is, screening, eligibility and inclusion in meta-analysis)	7
Study records - data collection process	#11c	Describe planned method of extracting data from reports (such as piloting forms, done independently, in duplicate), any processes for obtaining and confirming data from investigators	7
Data items	#12	List and define all variables for which data will be sought (such as PICO items, funding sources), any pre-planned data assumptions and simplifications	7
Outcomes and prioritization	#13	List and define all outcomes for which data will be sought, including prioritization of main and additional outcomes, with rationale	7
Risk of bias in individual studies	#14	Describe anticipated methods for assessing risk of bias of individual studies, including whether this will be done at the outcome or study level, or both; state how this information will be used in data synthesis	8
Data synthesis	#15a	Describe criteria under which study data will be quantitatively synthesised	8-9

For peer review only

Data synthesis	#15b	If data are appropriate for quantitative synthesis, describe planned summary measures, methods of handling data and methods of combining data from studies, including any planned exploration of consistency (such as I2, Kendall's τ)	8-9
Data synthesis	#15c	Describe any proposed additional analyses (such as sensitivity or subgroup analyses, meta-regression)	8-9
Data synthesis	#15d	If quantitative synthesis is not appropriate, describe the type of summary planned	8-9
Meta-bias(es)	#16	Specify any planned assessment of meta-bias(es) (such as publication bias across studies, selective reporting within studies)	8-9
Confidence in cumulative evidence	#17	Describe how the strength of the body of evidence will be assessed (such as GRADE)	NA

Notes:

- 10: 7 and Supplementary material The PRISMA-P checklist is distributed under the terms of the Creative Commons Attribution License CC-BY 4.0. This checklist was completed on 30. March 2020 using https://www.goodreports.org/, a tool made by the EQUATOR Network in collaboration with Penelope.ai

For peer review only

BMJ Open

Accuracy of potential diagnostic indicators for coeliac disease: a systematic review protocol

Journal:	*BMJ Open*
Manuscript ID	bmjopen-2020-038994.R1
Article Type:	Protocol
Date Submitted by the Author:	24-Aug-2020
Complete List of Authors:	Elwenspoek, Martha; NIHR ARC West, ARC West; University of Bristol Medical School, Populations Health Sciences<br>Jackson, Joni; NIHR ARC West, ARC West; University of Bristol Medical School, Populations Health Sciences<br>Dawson, Sarah; NIHR ARC West, ARC West; University of Bristol Medical School, Populations Health Sciences<br>Everitt, Hazel; University of Southampton, Primary Care and Population Sciences, Faculty of Medicine<br>Gillett, Peter; Royal Hospital for Sick Children, Hepatology and Nutrition Department<br>Hay, Alastair; University of Bristol, Centre for Academic Primary Care<br>Jones, Hayley E.; Univ Bristol, ; University of Bristol<br>Lane, Deborah<br>Mallett, Susan; University of Birmingham, School of Health & Population Sciences<br>Robins, Gerry; York Teaching Hospital NHS Foundation Trust, Department of Gastroenterology<br>Sheppard, Athena; University of Leicester, Department of Health Sciences<br>Stubbs, Jo<br>Thom, H; University of Bristol Medical School, Populations Health Sciences<br>Watson, Jessica; University of Bristol, School of Social and Community Medicine<br>Whiting, Penny; University of Bristol, School of Social and Community Medicine
**Primary Subject Heading**:	Gastroenterology and hepatology
Secondary Subject Heading:	Diagnostics
Keywords:	Coeliac disease < GASTROENTEROLOGY, Paediatric gastroenterology < PAEDIATRICS, Adult gastroenterology < GASTROENTEROLOGY

I, the Submitting Author has the right to grant and does grant on behalf of all authors of the Work (as defined in the below author licence), an exclusive licence and/or a non-exclusive licence for contributions from authors who are: i) UK Crown employees; ii) where BMJ has agreed a CC-BY licence shall apply, and/or iii) in accordance with the terms applicable for US Federal Government officers or employees acting as part of their official duties; on a worldwide, perpetual, irrevocable, royalty-free basis to BMJ Publishing Group Ltd ("BMJ") its licensees and where the relevant Journal is co-owned by BMJ to the co-owners of the Journal, to publish the Work in this journal and any other BMJ products and to exploit all rights, as set out in our licence.

The Submitting Author accepts and understands that any supply made under these terms is made by BMJ to the Submitting Author unless you are acting as an employee on behalf of your employer or a postgraduate student of an affiliated institution which is paying any applicable article publishing charge ("APC") for Open Access articles. Where the Submitting Author wishes to make the Work available on an Open Access basis (and intends to pay the relevant APC), the terms of reuse of such Open Access shall be governed by a Creative Commons licence – details of these licences and which Creative Commons licence will apply to this Work are set out in our licence referred to above.

Other than as permitted in any relevant BMJ Author's Self Archiving Policies, I confirm this Work has not been accepted for publication elsewhere, is not being considered for publication elsewhere and does not duplicate material already published. I confirm all authors consent to publication of this Work and authorise the granting of this licence.

For peer review only

Accuracy of potential diagnostic indicators for coeliac disease: a systematic review protocol

PROSPERO registration number: CRD42020170766

For peer review only

Authors

Martha M C Elwenspoek,[1,2] Joni Jackson,[1,2] Sarah Dawson,[2] Hazel Everitt,[3] Peter Gillett,[4] Alastair D Hay,[2] Hayley E Jones,[2] Deborah Lane,[5] Susan Mallett,[6] Gerry Robins,[7] Athena Sheppard,[8] Jo Stubbs,[5] Howard Thom,[2] Jessica Watson,[2] Penny Whiting.[2]

Affiliations

1. The National Institute for Health Research Applied Research Collaboration West (NIHR ARC West), University Hospitals Bristol NHS Foundation Trust, BS1 2NT Bristol, UK
2. Population Health Sciences, Bristol Medical School, University of Bristol, BS8 2PS Bristol, UK
3. Primary Care, Population Sciences and Medical Education, University of Southampton, Southampton SO16 5ST, UK
4. Paediatric Gastroenterology, Hepatology and Nutrition Department, Royal Hospital for Sick Children, Edinburgh EH9 1LF Scotland, UK
5. Patient representative
6. The Institute of Applied Health Research, College of Medical and Dental Sciences, University of Birmingham, Edgbaston, Birmingham, B15 2TT, UK
7. Department of Gastroenterology, York Teaching Hospital NHS Foundation Trust, York, UK
8. Department of Health Sciences, University of Leicester, Leicester, UK

Corresponding author

Martha M C Elwenspoek
9th Floor, Whitefriars, Lewins Mead,
Bristol, BS1 2NT
Email: Martha.Elwenspoek@bristol.ac.uk
Tel: +44/0 117 3427689

Email addresses co-authors

Joni.Jackson@bristol.ac.uk, Sarah.Dawson@bristol.ac.uk, H.A.Everitt@soton.ac.uk, Peter.Gillett65@gmail.com, Alastair.Hay@bristol.ac.uk, Hayley.Jones@bristol.ac.uk, Deborah.L.Lane@googlemail.com, S.Mallett@bham.ac.uk, Gerry.Robins@York.nhs.uk, als75@leicester.ac.uk, Jo.Stubbs@bristol.ac.uk, Howard.Thom@bristol.ac.uk, Jessica.Watson@bristol.ac.uk, Penny.Whiting@bristol.ac.uk

Word count: 2923

Abstract

Introduction

Coeliac disease (CD) is a systemic immune-mediated disorder triggered by gluten in genetically predisposed individuals. CD is diagnosed using a combination of serology tests and endoscopic biopsy of the small intestine. However, because of nonspecific symptoms and heterogeneous clinical presentation, diagnosing CD is challenging. Early detection of CD through improved case-finding strategies can improve the response to a gluten-free diet, patients' quality of life, and potentially reduce the risk of complications. However, there is a lack of consensus which groups may benefit from active case-finding.

Methods and analysis

We will perform a systematic review to determine the accuracy of diagnostic indicators (such as symptoms and risk factors) for CD in adults and children, and thus can help identify patients who should be offered CD testing. MEDLINE, Embase, Cochrane Library, and Web of Science will be searched from 1997 until 2020. Screening will be performed in duplicate. Data extraction will be performed by one and checked by a second reviewer. Disagreements will be resolved through discussion or referral to a third reviewer. We will produce a narrative summary of identified prediction models. Studies where 2x2 data can be extracted or reconstructed will be treated as diagnostic accuracy studies, i.e. the diagnostic indicators are the index tests and CD serology and/or biopsy is the reference standard. For each diagnostic indicator, we will perform a bivariate random effects meta-analysis of the sensitivity and specificity.

Ethics and dissemination

Results will be reported in peer-reviewed journals, academic and public presentations and social media. We will convene an implementation panel to advise on the optimum strategy for enhanced dissemination. We will discuss findings with Coeliac UK to help with dissemination to patients. Ethical approval is not applicable, as this is a systematic review and no research participants will be involved.

Registration number

CRD42020170766

Keywords

Coeliac disease, diagnosis, risk factors, symptoms, systematic review protocol.

For peer review only

Article summary

Strengths and limitations of this study

- This systematic review protocol follows the PRISMA Protocols guidelines, which offers transparency and enhances reproducibility.
- This review will follow guidelines from the Centre for Reviews and Dissemination and the Cochrane Handbook for Systematic Reviews of Diagnostic Test Accuracy, ensuring the use of robust and well-established methodologies.
- We will include studies that use serology tests and/or biopsy as reference standard to increase the inclusion rate, but we note that serology tests in particular can produce both false positive and false negative results (i.e. are not a 'gold standard'), which may lead to bias in the primary analysis.
- By balancing the sensitivity and specificity of our search strategy there is a small risk of missing relevant papers, although this will be mitigated by screening reference lists of relevant reviews.

For peer review only

Introduction

Coeliac disease (CD) is a systemic, immune-mediated disorder triggered by the ingestion of the protein gluten and related prolamins, found in wheat, rye and barley, in genetically predisposed individuals.[1 2] CD is characterised by small intestine damage, gluten-dependent clinical symptoms, and increased blood levels of CD-specific antibodies.[1] Patients typically present with gastrointestinal symptoms such as chronic diarrhoea, abdominal pain, or constipation, but can also present with non-specific symptoms such as tiredness, anaemia, or impaired growth in children.[3] In the UK and other European countries, the lifetime prevalence is estimated at around 1%.[4-7] CD is more frequently diagnosed in 'at-risk' individuals with certain conditions, such as type 1 diabetes,[8] Down's syndrome,[9] or iron deficiency anaemia,[10] and is 1.5 to 2 times more common in women than in men.[11]

In first-degree relatives CD prevalence is as high as 10%[12] and concordance in identical twins is 75% higher compared to nonidentical twins,[13] suggesting a strong genetic component. Certain human leukocyte antigen (HLA) variants are strongly associated with CD and almost all CD patients carry the HLA-DQ2/8 haplotype. However, this risk haplotype is common; up to 40% of the general population carry the HLA-DQ2/8 haplotype – most of whom will never develop CD.[14] Although environmental factors such as gluten exposure, breast feeding, and gastrointestinal infections are reported to play a role in the aetiology of CD,[15] the exact mechanism and pathophysiology of CD remain unclear.

CD is diagnosed using a combination of serology tests and endoscopic biopsy of the small intestine. These tests are only reliable when the patient is on a gluten containing diet. Current guidelines by the National Institute for Health and Care Excellence (NICE) recommend testing for total immunoglobulin A (IgA) and IgA tissue transglutaminase (tTG) as a first choice in both adults and children.[16] In case of IgA deficiency, immunoglobulin G (IgG) tTG, IgG endomysial antibody (EMA), or IgG deamidated gliadin peptide (DGP) can be measured. Seropositive adults should be referred for intestinal biopsy. Seropositive children should be referred for further investigation, which may include IgA EMA, intestinal biopsy, HLA genetic testing, or a combination thereof.[16]

The European Society for Paediatric Gastroenterology Hepatology and Nutrition (EPSGHAN) launched new guidelines in 2012[17] to avoid biopsies in children with high IgA tTG titres (>10 times upper limit of normal) and positive HLA-DQ2/8 and EMA results. Since then, new findings have supported this approach.[18] The updated EPSGHAN guidelines (2019) suggest that HLA-DQ2/8 testing can be omitted and the biopsy avoidance strategy can also be used in asymptomatic patients.[18] The biopsy avoidance strategy has been endorsed by the British Society of Paediatric Gastroenterology Hepatology and Nutrition (BSPGHAN) and Coeliac UK (2013) joint Guidelines for the diagnosis and management of coeliac disease in children,[4] but not yet by the NICE guidelines.[3] For adults, the biopsy avoidance strategy is currently not recommended.[3 19] However, a recent study suggests that adult CD can also be reliably diagnosed without biopsy in patients with high IgA tTG levels (>10× the upper limit of normal), positive EMA and HLA-DQ2/8 test results without requirement of symptoms.[20]

Patients diagnosed with CD are advised to follow a strict gluten-free diet, which is expensive and challenging to adhere to for patients, but it is the only available treatment. In most symptomatic patients a gluten-free diet reduces symptoms, increases quality of life, and seems to lower the risk of complications.[21-23] However, because of the nonspecific symptoms and heterogeneous clinical presentation, diagnosing CD is challenging. As a consequence, mean duration of symptoms before diagnosis is as long as 13 years in the UK[24] and 11 years in the US.[25] A delayed diagnosis can increase

For peer review only

the risk of more severe or non-responsive CD,[26] as well as the risk of severe complications such as osteoporosis,[27] infertility,[28] and lymphoma.[29]

Active case-finding strategies, i.e. offering tests to individuals with certain symptoms or conditions associated with CD, aim to improve timeliness of CD diagnosis. Early detection of CD through improved case-finding strategies can improve the response to a gluten-free diet, patients' quality of life, and potentially reduce the risk of complications. However, there is a lack of consensus which groups may benefit from active case-finding. Therefore, we will perform a systematic review of the literature to investigate which diagnostic indicators (such as symptoms and risk factors) are most sensitive and specific for CD, and thus can help identify patients that should be offered CD testing.

Aim

The overall aim of this review is to determine the accuracy of diagnostic indicators for CD in adults and children.

Objectives

1. Determine the accuracy of diagnostic indicators to diagnose CD in children (≤ 16 years old).
2. Determine the accuracy of diagnostic indicators in adults (> 16 years old).
3. Identify combinations of diagnostic indicators that lead to an increased probability of coeliac disease.

For peer review only

Methods

The review will follow Centre for Reviews and Dissemination (CRD) guidelines for undertaking systematic reviews[30] and methods described in the Cochrane Handbook for Systematic Reviews of Diagnostic Test Accuracy[31] and has been registered on the PROSPERO database (CRD42020170766). This protocol is reported according to the PRISMA guidance for systematic review protocols and the PRISMA statement for diagnostic test accuracy studies.[32 33] Protocol amendments will be documented and published together with the results of the systematic review.

Eligibility criteria

Studies that fulfil the following criteria will be eligible for inclusion:

- **Population**: Adults and/or children (≤ 16 years old) tested for CD.
- **Diagnostic indicators (treated as 'index tests')**: Any diagnostic indicator will be eligible for inclusion. We consider diagnostic indicators to be those that may assist a clinician in making a diagnosis during an initial consultation, for example: family history or recent test results (e.g. full blood count to test for anaemia), current symptoms, or a risk HLA-DQ genotype. Factors that are more general risk factors rather than potential diagnostic indicators such as perinatal factors, 'susceptibility genes' (other than HLA-DQ status), or age at gluten introduction will be excluded. Tests for susceptibility genes are currently not widely available to clinicians and therefore not (yet) useful in aiding diagnosis.
- **Reference standards:** the following reference standards are acceptable, (1) all patients undergo coeliac specific serological tests and duodenal biopsy, (2) all patients undergo coeliac specific serological tests and seropositive patients undergo a duodenal biopsy, (3) all patients undergo duodenal biopsy without serology tests, (4) patients undergo one or a combination of the following coeliac specific serology tests: IgA/IgG tTG, EMA, or DGP. Studies where not all patients have received the reference standard will be excluded.
- **Study design**: Studies using a "single-gate" or "multi-gate" design and prediction modelling studies are eligible for inclusion. In a single-gate design, a single set of inclusion criteria (gate) is used for all participants, whereas in a multi-gate design, two or more sets of inclusion criteria (gates) are used (e.g. one for a risk factor group or a group of coeliac patients and another for healthy controls).[34] Control groups, i.e. study participants without the diagnostic indicator or without CD, are required to be representative of the general population. Diagnostic indicator studies will be treated as diagnostic test accuracy studies and we will refer to these studies as such throughout this protocol. If sufficient single-gate studies are identified, then multi-gate studies will be excluded, as the latter have been shown to overestimate test accuracy.[35] Diagnostic accuracy studies will be required to report sufficient data to construct 2x2 tables showing the number of participants with and without CD (according to the reference standard) cross-classified against the number with and without the diagnostic indicator. There will be no specific data requirements for prediction modelling studies.
- **Additional restrictions**: We will exclude studies published before 1997 (the year in which the most important serological test, tTG, was developed), to reduce the variation in the methods used for diagnosing coeliac disease (reference standard). We will not apply any restrictions based on patient age, language, or study size.

For peer review only

Information sources

MEDLINE, Embase, Cochrane Library, and Web of Science will be searched. Ongoing and completed studies will be identified using the WHO International Clinical Trials Registry and the NIH Clinical Trials database, and internet searches will be undertaken. Reference lists of the latest guidelines from NICE and the British Society of Gastroenterology on CD and systematic reviews published after the latest NICE guidelines will be screened.

Search strategy

The search strategy incorporates three main elements: (1) conditions (CD) + prognostic/predictive research filter (based on Geersing[36] and Ingui's[37] work), (2) conditions (CD) + all physical diseases/ signs/symptoms (based on MeSH, EMTREE) + 'CD' diagnosis, (3) terms for high risk populations (see Supplementary material for a detailed search strategy). We will exclude animal studies, case reports, letters, editorials, and coeliac artery/trunk research and will apply a sensitive study design filter. No language or publication restrictions will be applied.

Study records

Data management

Identified references will be downloaded into Endnote X9 software for further assessment and handling. Rigorous records are maintained as part of the searching process.

Selection process

Study selection will be conducted in two stages using forms developed in Microsoft Access: (1) an initial screening of titles and abstracts against the inclusion criteria to identify potentially relevant papers, (2) screening of the full papers identified as possibly relevant in the initial screening. All papers excluded at the second stage will be documented along with the reasons for exclusion. Abstracts and full texts will be screened independently by at least two researchers. Disagreements about study eligibility will be resolved through discussion or by consulting a third reviewer.

Data collection process (data extraction)

Data will be extracted using standardised data extraction forms developed in Microsoft Access. Data extraction forms will be piloted on a small sample of papers and adapted as necessary. In order to minimise bias and errors, data extraction will be performed by one reviewer and checked by a second. Disagreements will be resolved through discussion or referral to a third reviewer.

To exclude diagnostic indicators with insufficient evidence, we will only extract data on diagnostic indicators that are reported in five or more studies, with the exception of "new but exceptionally promising" diagnostic indicators according to our expert panel (i.e. widely used diagnostic indicators that are deemed too important for omission). We will extract the following data from the remaining papers, where reported: study characteristics, patient characteristics, details on the diagnostic indicator, and details on how CD was diagnosed (i.e. the reference standard).

Diagnostic accuracy studies: Numerical results will be extracted as 2x2 contingency tables (i.e. number of individuals with versus without the diagnostic indicator, tabulated against CD status). Where possible, data will be extracted separately for different age groups (children and adults), sex (male and female), and symptomatic groups ("at risk" asymptomatic, symptomatic primary care or symptomatic secondary care). If 2x2 data is not reported, we will reconstruct this from estimates of sensitivity, specificity, positive and negative predictive values, or unadjusted diagnostic odds ratios,

For peer review only

intercept, and prevalence data where possible. If this is not possible the study will be excluded. We will contact authors if there are inconsistencies in the data.

Prediction modelling studies: We will extract details from the final model, including which variables were used, whether the model was validated, and measures of model performance. If 2x2 data (or sufficient data from which to recover this, such as unadjusted ORs, intercepts, prevalence) are reported for individual diagnostic indicators, these will also be extracted.

Risk of bias in individual studies

As we are treating diagnostic indicator studies as diagnostic test accuracy studies, risk of bias will be assessed using the QUADAS-2 tool[38] or QUADAS-3 tool if available (currently under development), which includes domains covering participants, index test, reference standard and flow and timing. Prediction modelling studies will be assessed using the PROBAST tool,[39] which includes domains covering participant selection, outcome, predictors, sample size and flow, and analysis. The content of the tools will be tailored to the review by adding guidance for interpreting signalling questions as appropriate. The tailored tools will be combined and piloted on a small number of studies by two reviewers and agreement assessed. If agreement is low, the tool and/or guidelines will be further refined until satisfactory agreement is reached and the tool can be applied to all included studies. If at least one of the domains is rated as “high risk” the study will be considered at high risk of bias, if all domains are judged as “low risk” the trial will be considered at low risk of bias, otherwise the trial will be considered at “unclear” risk of bias. The risk of bias assessment will be conducted as part of the data extraction process.

Data synthesis

A narrative summary of the included studies will be presented. This will include a summary of the characteristics (e.g. study design, population size, geographical location, year, baseline population characteristics, diagnostic indicators evaluated, and reference standard). A detailed commentary on the major methodological problems or biases that affected the studies will also be included.

The analysis will be stratified by age group (children ≤ 16 years old, adults, mixed) and we will group study results according to type of diagnostic indicator (symptoms, risk conditions, genetic risk factors). For each group, we will plot study-specific estimates of sensitivity and specificity in receiver operating characteristic (ROC) space and/or produce coupled forest plots.

To exclude diagnostic indicators with insufficient evidence, we will only perform meta-analyses on diagnostic indicators where 2x2 data is available from five studies or more. We will perform a bivariate random effects meta-analysis of the sensitivity and specificity on each remaining diagnostic indicator.[40 41] This will produce summary estimates of the sensitivity and specificity of each diagnostic indicator, with 95% confidence or credible ellipses, and 95% prediction ellipses. Prediction ellipses are visual representations of the amount of between-study heterogeneity in both dimensions of a diagnostic test meta-analysis. We will also report estimates of the between-study standard deviation in sensitivity and specificity on the logit scale.

Summary results from each meta-analysis will also be used to estimate positive and negative predictive values (PPVs and NPVs), i.e. the probability of CD given that the individual has (or does not have) each diagnostic indicator. Calculation of these values will require an assumed value for the prevalence of CD in the general population. This could be based on studies in the meta-analysis, or on other evidence. If estimates of prevalence are heterogeneous, we will present data or plot predictive values across a range of plausible prevalences.

For peer review only

Exploration of heterogeneity: We expect heterogeneity in study estimates of sensitivity and specificity due to variability in patient populations, reference standard definitions or methods, study design, and other study characteristics.

If sufficient data are available, we will use subgroup analysis and/or meta-regression to investigate whether the identified diagnostic indicators differ in accuracy between: (i) setting (asymptomatic "at risk", primary care symptomatic, and secondary care symptomatic patients), (ii) children vs adults (iii) CD diagnosis (biopsy and serology versus serology only), (iv) study design (single-gate versus multi-gate), (v) men versus women, (vi) low versus high risk of bias. We will also explore the possibility of adjusting for the imperfect accuracy of the serological tests in a Bayesian statistical framework, using informative prior distributions for the sensitivity and specificity of these tests based on an ongoing systematic review on the accuracy of serology tests for CD.[42]

All statistical analyses for will be performed in R and WinBUGS (version 14).

Prediction modelling studies: We will also produce a narrative summary of prediction models, describing which variables were included in the final model, measures of model performance, and whether the model was validated.

Patient and public involvement

Our team includes two patient representatives who were involved in development of the research project, of which this review is a part. The patient representatives will be invited to join regular team meetings to provide patient perspectives on the research to ensure that it addresses patient needs. They will also be involved in the interpretation and dissemination of the results from this review.

Ethics and dissemination

Results will be reported in peer-reviewed journals, academic, and public presentations and social media. Results will also be expressed in terms of hypothetical populations of 1000 patients for a plain language summary of analysis results. We will convene an implementation panel to advise on the optimum strategy for enhanced dissemination. We will discuss findings with the charity Coeliac UK to help with dissemination to patients.

Timeline

This systematic review will be finalised by April 2021 (see Table 1 for detailed timeline of the project).

Table 1. Project timeline

**Task**	**Deadline**
Protocol development	February 2020
Searches	February 2020
Abstract and full text screening	April 2020
Data extraction	September 2020
Analysis	January 2021
Manuscript submission	April 2021

For peer review only

For peer review only

Discussion

This systematic review is part of a larger project that aims to identify cost-effective strategies for active case-finding of CD in different at-risk populations. The information identified in the review will feed into economic models to determine the cost-effectiveness of active case-finding strategies, which will help commissioners, clinicians, and patients make evidence-based decisions on whether active case-finding for CD should be undertaken in primary care. There is evidence that CD is under-diagnosed in both children and adults.[6 43] Appropriate identification and treatment of CD can have significant benefits for patients in terms of symptoms, quality of life, and long-term health outcomes, as well as reducing healthcare and societal economic costs.[24 44] Different guidelines recommend different diagnostic pathways showing a lack of evidence and consensus on which groups may benefit from active case-finding, the best method of doing this, and the costs, benefits and potential harms. A rigorously undertaken high-quality evidence synthesis including health economic modelling will provide a robust summary of the current evidence base and highlight whether there is sufficient evidence to suggest an optimum strategy or whether further research is needed.

For peer review only

Funding statement

This work was supported by a National Institute for Health Research (NIHR) Health Technology Assessment Programme grant number NIHR129020. This publication presents independent research funded by the NIHR. This research was also supported by the National Institute for Health Research (NIHR) Applied Research Collaboration West (NIHR ARC West). The views expressed in this article are those of the author(s) and not necessarily those of the NIHR or the Department of Health and Social Care.

Competing interest statement

No competing interests.

Author contributions

PW conceptualised the project and obtained funding together with ME, HE, PG, AH, HJ, DL, SM, GR, AS, JS, HT, and JW. PW, ME, HE, PG, AH, HJ, SM, GR, AS, HT, and JW designed the protocol. SD, ME, AS and PW developed a search strategy. PW, ME, HJ, JJ and SM planned the data extraction and statistical analysis. HE, PG, AH, GR, and JW provided clinical insights and DL and JS provided patient perspectives. ME drafted the initial manuscript. JJ, SD, HE, PG, AH, HJ, SM, GR, AS, HT, JW, and PW contributed to the final written manuscript. All authors reviewed the manuscript and have approved and agree to be accountable for all aspects of the work in ensuring that questions related to the accuracy or integrity of any part of the work are appropriately investigated and resolved.

For peer review only

References

1. Lindfors K, Ciacci C, Kurppa K, et al. Coeliac disease. *Nature Reviews Disease Primers* 2019;5(1):3. doi: 10.1038/s41572-018-0054-z
2. Ludvigsson JF, Leffler DA, Bai JC, et al. The Oslo definitions for coeliac disease and related terms. *Gut* 2013;62(1):43-52. doi: 10.1136/gutjnl-2011-301346 [published Online First: 2012/02/22]
3. Internal Clinical Guidelines Team 2015. Clinical Guideline NG20 Coeliac Disease: Recognition, assessment and management. *National Institute for Health and Care Excellence (NICE),* 2015
4. Murch S, Jenkins H, Auth M, et al. Joint BSPGHAN and Coeliac UK guidelines for the diagnosis and management of coeliac disease in children. *Arch Dis Child* 2013;98(10):806-11. doi: 10.1136/archdischild-2013-303996 [published Online First: 2013/08/30]
5. Mustalahti K, Catassi C, Reunanen A, et al. The prevalence of celiac disease in Europe: results of a centralized, international mass screening project. *Ann Med* 2010;42(8):587-95. doi: 10.3109/07853890.2010.505931 [published Online First: 2010/11/13]
6. Bingley PJ, Williams AJ, Norcross AJ, et al. Undiagnosed coeliac disease at age seven: population based prospective birth cohort study. *BMJ* 2004;328(7435):322-3. doi: 10.1136/bmj.328.7435.322 [published Online First: 2004/02/07]
7. West J, Logan RF, Hill PG, et al. Seroprevalence, correlates, and characteristics of undetected coeliac disease in England. *Gut* 2003;52(7):960-5. doi: 10.1136/gut.52.7.960 [published Online First: 2003/06/13]
8. Ludvigsson JF, Ludvigsson J, Ekbom A, et al. Celiac disease and risk of subsequent type 1 diabetes: a general population cohort study of children and adolescents. *Diabetes Care* 2006;29(11):2483-8. doi: 10.2337/dc06-0794 [published Online First: 2006/10/27]
9. Marild K, Stephansson O, Grahnquist L, et al. Down syndrome is associated with elevated risk of celiac disease: a nationwide case-control study. *J Pediatr* 2013;163(1):237-42. doi: 10.1016/j.jpeds.2012.12.087 [published Online First: 2013/02/13]
10. Hin H, Bird G, Fisher P, et al. Coeliac disease in primary care: case finding study. *BMJ* 1999;318(7177):164-7. doi: 10.1136/bmj.318.7177.164 [published Online First: 1999/01/15]
11. Murray JA, Van Dyke C, Plevak MF, et al. Trends in the identification and clinical features of celiac disease in a North American community, 1950-2001. *Clin Gastroenterol Hepatol* 2003;1(1):19-27. doi: 10.1053/jcgh.2003.50004 [published Online First: 2004/03/16]
12. Rubio-Tapia A, Van Dyke CT, Lahr BD, et al. Predictors of family risk for celiac disease: a population-based study. *Clin Gastroenterol Hepatol* 2008;6(9):983-7. doi: 10.1016/j.cgh.2008.04.008 [published Online First: 2008/07/01]
13. Greco L, Romino R, Coto I, et al. The first large population based twin study of coeliac disease. *Gut* 2002;50(5):624-8. doi: 10.1136/gut.50.5.624 [published Online First: 2002/04/16]
14. Jamnik J, Villa CR, Dhir SB, et al. Prevalence of positive coeliac disease serology and HLA risk genotypes in a multiethnic population of adults in Canada: a cross-sectional study. 2017;7(10):bmjopen-2017-017678. doi: 10.1136/bmjopen-2017-017678 %J BMJ Open
15. Meijer C, Shamir R, Szajewska H, et al. Celiac Disease Prevention. *Front Pediatr* 2018;6:368. doi: 10.3389/fped.2018.00368 [published Online First: 2018/12/18]
16. Downey L, Houten R, Murch S, et al. Recognition, assessment, and management of coeliac disease: summary of updated NICE guidance. *BMJ* 2015;351:h4513. doi: 10.1136/bmj.h4513 [published Online First: 2015/09/04]
17. Husby S, Koletzko S, Korponay-Szabo IR, et al. European Society for Pediatric Gastroenterology, Hepatology, and Nutrition guidelines for the diagnosis of coeliac disease. *J Pediatr Gastroenterol Nutr* 2012;54(1):136-60. doi: 10.1097/MPG.0b013e31821a23d0 [published Online First: 2011/12/27]
18. Husby S, Koletzko S, Korponay-Szabo I, et al. European Society Paediatric Gastroenterology, Hepatology and Nutrition Guidelines for Diagnosing Coeliac Disease 2020. *J Pediatr*

For peer review only - http://bmjopen.bmj.com/site/about/guidelines.xhtml

Gastroenterol Nutr 2020;70(1):141-56. doi: 10.1097/MPG.0000000000002497 [published Online First: 2019/10/01]
19. Ludvigsson JF, Bai JC, Biagi F, et al. Diagnosis and management of adult coeliac disease: guidelines from the British Society of Gastroenterology. *Gut* 2014;63(8):1210-28. doi: 10.1136/gutjnl-2013-306578 [published Online First: 2014/06/12]
20. Fuchs V, Kurppa K, Huhtala H, et al. Serology-based criteria for adult coeliac disease have excellent accuracy across the range of pre-test probabilities. *Aliment Pharmacol Ther* 2019;49(3):277-84. doi: 10.1111/apt.15109 [published Online First: 2018/12/29]
21. Murray JA, Watson T, Clearman B, et al. Effect of a gluten-free diet on gastrointestinal symptoms in celiac disease. *Am J Clin Nutr* 2004;79(4):669-73. doi: 10.1093/ajcn/79.4.669 [published Online First: 2004/03/31]
22. Sanchez MI, Mohaidle A, Baistrocchi A, et al. Risk of fracture in celiac disease: gender, dietary compliance, or both? *World J Gastroenterol* 2011;17(25):3035-42. doi: 10.3748/wjg.v17.i25.3035 [published Online First: 2011/07/30]
23. Holmes GK, Prior P, Lane MR, et al. Malignancy in coeliac disease--effect of a gluten free diet. *Gut* 1989;30(3):333-8. doi: 10.1136/gut.30.3.333 [published Online First: 1989/03/01]
24. Gray AM, Papanicolas IN. Impact of symptoms on quality of life before and after diagnosis of coeliac disease: results from a UK population survey. *BMC Health Serv Res* 2010;10:105. doi: 10.1186/1472-6963-10-105 [published Online First: 2010/04/29]
25. Green PHR, Stavropoulos SN, Panagi SG, et al. Characteristics of adult celiac disease in the USA: results of a national survey. *Am J Gastroenterol* 2001;96(1):126-31. doi: 10.1111/j.1572-0241.2001.03462.x [published Online First: 2001/02/24]
26. Paarlahti P, Kurppa K, Ukkola A, et al. Predictors of persistent symptoms and reduced quality of life in treated coeliac disease patients: a large cross-sectional study. *BMC Gastroenterol* 2013;13:75. doi: 10.1186/1471-230X-13-75 [published Online First: 2013/05/02]
27. Capriles VD, Martini LA, Areas JA. Metabolic osteopathy in celiac disease: importance of a gluten-free diet. *Nutr Rev* 2009;67(10):599-606. doi: 10.1111/j.1753-4887.2009.00232.x [published Online First: 2009/09/30]
28. Gasbarrini A, Torre ES, Trivellini C, et al. Recurrent spontaneous abortion and intrauterine fetal growth retardation as symptoms of coeliac disease. *Lancet* 2000;356(9227):399-400. doi: 10.1016/S0140-6736(00)02535-6 [published Online First: 2000/09/06]
29. Elfstrom P, Granath F, Ekstrom Smedby K, et al. Risk of lymphoproliferative malignancy in relation to small intestinal histopathology among patients with celiac disease. *J Natl Cancer Inst* 2011;103(5):436-44. doi: 10.1093/jnci/djq564 [published Online First: 2011/02/04]
30. Centre for Reviews and Dissemination (CRD). Systematic Reviews. CRD's guidance for undertaking reviews in health care. *University of York* 2009
31. Cochrane Diagnostic Test Accuracy Working Group. Cochrane Handbook for Systematic Reviews of Diagnostic Test Accuracy. *The Cochrane Collaboration* 2010
32. Shamseer L, Moher D, Clarke M, et al. Preferred reporting items for systematic review and meta-analysis protocols (PRISMA-P) 2015: elaboration and explanation. *BMJ* 2015;350:g7647. doi: 10.1136/bmj.g7647 [published Online First: 2015/01/04]
33. McInnes MDF, Moher D, Thombs BD, et al. Preferred Reporting Items for a Systematic Review and Meta-analysis of Diagnostic Test Accuracy Studies: The PRISMA-DTA Statement. *JAMA* 2018;319(4):388-96. doi: 10.1001/jama.2017.19163 [published Online First: 2018/01/25]
34. Rutjes AW, Reitsma JB, Vandenbroucke JP, et al. Case-control and two-gate designs in diagnostic accuracy studies. *Clin Chem* 2005;51(8):1335-41. doi: 10.1373/clinchem.2005.048595 [published Online First: 2005/06/18]
35. Whiting PF, Rutjes AW, Westwood ME, et al. A systematic review classifies sources of bias and variation in diagnostic test accuracy studies. *J Clin Epidemiol* 2013;66(10):1093-104. doi: 10.1016/j.jclinepi.2013.05.014 [published Online First: 2013/08/21]

For peer review only

36. Geersing GJ, Bouwmeester W, Zuithoff P, et al. Search filters for finding prognostic and diagnostic prediction studies in Medline to enhance systematic reviews. *PLoS One* 2012;7(2):e32844. doi: 10.1371/journal.pone.0032844 [published Online First: 2012/03/07]
37. Ingui BJ, Rogers MA. Searching for clinical prediction rules in MEDLINE. *J Am Med Inform Assoc* 2001;8(4):391-7. doi: 10.1136/jamia.2001.0080391 [published Online First: 2001/06/22]
38. Whiting PF, Rutjes AW, Westwood ME, et al. QUADAS-2: a revised tool for the quality assessment of diagnostic accuracy studies. *Ann Intern Med* 2011;155(8):529-36. doi: 10.7326/0003-4819-155-8-201110180-00009 [published Online First: 2011/10/19]
39. Wolff RF, Moons KGM, Riley RD, et al. PROBAST: A Tool to Assess the Risk of Bias and Applicability of Prediction Model Studies. *Ann Intern Med* 2019;170(1):51-58. doi: 10.7326/M18-1376 [published Online First: 2019/01/01]
40. Reitsma JB, Glas AS, Rutjes AW, et al. Bivariate analysis of sensitivity and specificity produces informative summary measures in diagnostic reviews. *J Clin Epidemiol* 2005;58(10):982-90. doi: 10.1016/j.jclinepi.2005.02.022 [published Online First: 2005/09/20]
41. Chu H, Cole SR. Bivariate meta-analysis of sensitivity and specificity with sparse data: a generalized linear mixed model approach. *J Clin Epidemiol* 2006;59(12):1331-2; author reply 32-3. doi: 10.1016/j.jclinepi.2006.06.011 [published Online First: 2006/11/14]
42. Sheppard AL, Whiting P, Corfield V, et al. What is the accuracy of serological testing for diagnosing coeliac disease in adults and children? A systematic review: PROSPERO, 2019.
43. Ravikumara M, Nootigattu VK, Sandhu BK. Ninety percent of celiac disease is being missed. *J Pediatr Gastroenterol Nutr* 2007;45(4):497-9. doi: 10.1097/MPG.0b013e31812e5710 [published Online First: 2007/11/22]
44. Mearns ES, Taylor A, Boulanger T, et al. Systematic Literature Review of the Economic Burden of Celiac Disease. *Pharmacoeconomics* 2019;37(1):45-61. doi: 10.1007/s40273-018-0707-5 [published Online First: 2018/09/18]

For peer review only

Supplementary materials

Search strategy

Database: Embase <1974 to 2020 Week 05>

Search Strategy: *ARC-Coeliac-Embase-Final*

----

1 Celiac Disease/ (29729)

2 c?eliac.ti,kw. (22813)

3 c?eliac.af. and gastroenterology.ec. (22352)

4 (celiac or coeliac).ab. /freq=3 (9328)

5 or/1-4 (39462)

6 ((detect* or diagnos* or identif* or decision* or predict* or prognos* or risk or risks or stratif* or validat*) adj3 (model* or factor* or algorithm? or scor* or system or technique? or aid? or rule or rules or index or variable* or tool? or panel or criteri* or characteristic? or history or finding* or value* or assay or stratif* or biomarker?)).ti,ab,kw. (2855495)

7 ((detect* or diagnos* or identif* or decision* or predict* or prognos* or stratif* or validat*) adj3 (risk or risks or model* or factor* or algorithm? or scor* or system or technique? or aid? or rule or rules or index or variable* or tool? or panel or criteri* or characteristic? or history or finding* or value* or assay or stratif* or biomarker?)).ti,ab,kw. (2225766)

8 (clinic* adj3 (model* or algorithm? or scor* or risk or risks or rule or rules or predict* or index or tool? or panel or criteri* or decision* or stratif* or diagnos* or detect* or identif* or probabilit*)).ti,ab,kw. (632961)

9 ((multivaria* or multicomponent?) adj3 (model* or algorithm? or scor* or rule or rules or index or tool? or panel or criteri*)).ti,ab,kw. (110662)

10 clinical decision making/ or statistical model/ or algorithm/ (444594)

11 or/6-10 (3678637)

12 5 and 11 (6036)

13 *physical disease by body function/ or exp *abnormal blood pressure/ or exp *abnormal posture/ or *aerophagia/ or exp *appetite disorder/ or *asthenia/ or *asymptomatic disease/ or exp *autonomic dysfunction/ or exp *balance disorder/ or exp *blood clotting disorder/ or exp *body temperature disorder/ or exp *body weight disorder/ or exp *common cold symptom/ or exp *consciousness disorder/ or exp *constipation/ or exp *coughing/ or exp *cyanosis/ or exp *diarrhea/ or exp *disability/ or exp *"disorders of higher cerebral function"/ or exp *dysphagia/ or *eructation/ or exp *faintness/ or exp *fatigue/ or *functional disease/ or exp *growth disorder/ or exp *immunopathology/ or exp *incontinence/ or exp *infertility/ or *listlessness/ or *malaise/ or *meningism/ or exp *metabolic disorder/ or exp *micturition disorder/ or exp *motor dysfunction/ or exp *multiple organ failure/ or exp *"nausea and vomiting"/ or exp *nutritional disorder/ or exp

For peer review only

*pain/ or *pallor/ or exp *pregnancy disorder/ or *qi deficiency/ or *qi stagnation/ or exp *reflex disorder/ or exp *respiratory function disorder/ or exp *salivation disorder/ or exp *sensory dysfunction/ or exp *sexual dysfunction/ or exp *shock/ or exp *sleep disorder/ or exp *speech disorder/ or *weakness/ or *yang deficiency/ or *yin deficiency/ (4991033)

14 *physical disease by anatomical structure/ or exp *abdominal disease/ or exp *abnormal body build/ or exp *breast disease/ or exp *cardiovascular disease/ or exp *connective tissue disease/ or exp *digestive system disease/ or exp *ear nose throat disease/ or exp *endocrine disease/ or exp *eye disease/ or exp *"head and neck disease"/ or exp *hematologic disease/ or exp *mouth disease/ or exp *mucosal disease/ or exp *musculoskeletal disease/ or exp *neurologic disease/ or exp *pelvic disease/ or exp *respiratory tract disease/ or exp *skin disease/ or exp *soft tissue disease/ or exp *thorax disease/ or exp *urogenital tract disease/ (11959905)

15 *"physical disease by composition of body fluids, excreta and secretions"/ or exp *abnormal feces composition/ or exp *abnormal substrate concentration in blood/ or exp *abnormal urine composition/ or *dehydration/ or *hypervolemia/ (344197)

16 ((addison* adj3 (disease* or disorder* or syndrom*)) or alopecia or baldness or amenorrhea* or amenorrhoea* or oligomenorrhea* or oligomenorrhoea* or anxiety disorder* or anorexia or adrenal insufficienc* or adrenocortical insufficienc* or allerg* or anemi* or anaemi* or angina or aneurysm or ankylosing spondylitis or arthropath* or arthriti* or arthrosis or arthroses or asthma* or asthenia or ataxia or atrial fibrillation or atrophy or autoimmune disorder* or (autoimmune adj (disease* or disorder* or syndrom*)) or ataxia or avitaminosis or bonnevie ullrich or back pain or (biliary adj3 (disease* or disorder* or syndrom*)) or (bipolar adj3 disorder*) or blindness or blind loop syndrom* or (blood adj3 poisoning) or brain atroph* or ((bone or bones) adj3 (broken or disease* or disorder* or syndrom*)) or (bone* adj3 (mineral* or densit* or soft* or decay*)) or (brain adj3 (disease* or disorder* or syndrom*)) or (bronchi* adj3 (disease* or disorder* or syndrom*)) or (bowel* adj3 (disease* or disorder* or syndrom*)) or calcinosis or cancer* or carcinoma* or adenocarcinoma* or neoplasm* or neoplastic or metasta* or malignan* or tumour or tumours or tumor or tumors or (cardiac adj3 (arrest or arrhythmia* or disease* or disorder* or surg* or syndrom*)) or cardiomyopath* or ((cardiovascular or coronary) adj3 (disease* or disorder* or event* or syndrom*)) or cachexia or ((cecal or colon* or duoden*) adj3 (disease* or disorder* or syndrom*)) or cerebral palsy or (cerebro* adj3 (degenerat* or disease* or disorder* or event* or syndrom*)) or chronic obstructive disease* or cirrhosis or claudication or colic or copd or ((coordination or co-ordination) adj3 (impair* or lack)) or congenital abnormalit* or (congential adj3 (disease* or disorder* or syndrom*)) or ((connective or collagen* or skin) adj3 (disease* or disorder* or syndrom*)) or collagenous sprue or colitis or colotides or coxarthrosis or crohn* or cushing* or cyanosis or cystic fibrosis or cystitis or deaf* or deformit* or delayed puberty or dental or depressive disorder* or dysphoria or dysthymia or disabled or (physical adj3 (deform* or disab* or impair*)) or dermatitis or dermato* or dorsopath* or diabet* or down* syndrom* or duhring* or duehring* or duhrig* or duehrig* or dyscoordination or dys-coordination or dys-coordination or dys-co-ordination or dysentery or dyssynergia or dys-synergia or emaciat* or enteritis or enterocolitis* or enteropathy or dystonia or eczema or edema or oedema or elfin face sydrom* or encephalopath* or (endocrine adj3 (disease* or disorder* or syndrom*)) or enuresis or enteropath* or epilep* or (eye adj3 (disease* or disorder* or manifestation* or syndrom*)) or fatigue syndrome or chronic fatigue or failure to thrive or fibromyalgia or fibrosis or food hypersensitivity or fractures or gammaglobulinemia or gammaglobulinaemia or (gardner* adj3 (syndrom* or disease* or disorder*)) or gastritis or gastroenteritis or gout or (glomerul* adj3 (disease* or disorder* or syndrom*)) or (gonodal adj3 dysgen*) or (growth adj3 (disease* or disorder* or syndrom*)) or headache* or

For peer review only

((hemic or haemic or lymph*) adj3 (disease* or disorder* or syndrom*)) or hematuria or haematuria or hemophili* or haemophili* or ((hearing or visual or vision or sight) adj3 (aid* or impair* or loss)) or (heart adj3 (disease* or disorder* or syndrom* or manifestation* or myopath*)) or hemiplegi* or hepatitis or hemodialysis or haemodialysis or (heart adj3 (disease* or disorder* or failure or syndrom*)) or hiv or human immunodeficiency virus or acquired immunodeficieny syndrom* or heerfordt waldenstrom* or hidroa or hydroa or hippel lindau* or hypertensi* or hypotensi* or hyperthyroidism or hypothyroidism or hypocortisolism or hypocorticism or hypoadrenalism or hyperthyroidism or hypothyroidism or incoordination or inco-ordination or in-coordination or in-co-ordination or infertility or subfertility or sub-fertility or sterility or inflammatory disease* or inflammatory bowel disease* or incontinen* or intestinal atresi* or intussusception or irritable bowel or ischemi* or ischaemi* or fistula* or (joint adj3 (disease* or disorder* or syndrom*)) or (jejunal adj3 (disease* or disorder* or syndrom*)) or kyphosis or lav-htlv* or leukemia or leukaemia or ((liver or hepatic) adj3 (disease* or disorder* or failure or syndrom*)) or lordosis or (lung adj3 (disease* or disorder*)) or lupus or lymphoma or lymphogranuloma* or lymphadenopath* or lymphotrop* or machado joseph* or macular degeneration or malnutrition or (mental adj2 (disorder* or disease* or health or illness*)) or mania or manic or (menstr* adj3 (ceas* or disturb* or disease* or disorder* or stop* or syndrom*)) or migraine* or (mitochondrial adj3 (disease* or disorder* or syndrom*)) or movement disorder* or mucinosis or musculoskeletal or narp syndrom* or necrotizing or nephrotic* or neuromuscular or non-hodgkin* or nonhodgkin* or multiple sclerosis or myeloma or myocarditis or myocardiopath* or myopath* or (myocardi* adj3 (deteri* or disease* or disorder* or syndrom* or manifestation*)) or nephrotic syndrome* or ((neurodegenerat* or neuro-degenerat) adj3 (disease* or disorder* or syndrom*)) or ((nutritional or metabolic) adj3 (disease* or disorder* or syndrom*)) or ((organ* or kidney or stem cell) adj3 (transplant* or recipient*)) or (nervous system adj3 (disease* or disorder* or syndrom*)) or (neurological adj3 (condition* or disease* or disorder* or syndrom*)) or neuropath* or neurosarcoido* or occlusion* or obesity or obese or orthopedic* or orthopaedic* or ((esophageal or oesophageal) adj3 (disease* or disorder* or syndrom*)) or ((oral or mouth) adj3 (disease* or disorder* or syndrom* or manifestation*)) or osteo* or otitis media or otorhinolaryngolog* or otosclerosis or (pancrea* adj3 (disease* or disorder* or syndrom*)) or papulosquamous or paraplegi* or parkinson* or (peripheral adj3 (arterial or vascular or disease* or disorder* or syndrom*)) or (peritoneal adj3 (disease* or disorder* or syndrom*)) or photodermato* or pick disease* or pneumo* or polio* or polyp* or polydipsia or polyarthropath* or polyarteritis or polyarthrosis or polyneuropath* or porphyrias or (pregnancy adj3 complicat*) or premature aging or proteostasis or pseudophakia or psoriasis or parapsoriasis or prolapse or (pulmonary adj3 (disease* or disorder* or syndrom*)) or purpur* or (recurren* adj3 (abortion* or miscarr*)) or (rect* adj3 (disease* or disorder* or syndrom*)) or ((renal or kidney) adj3 (disease* or disorder* or failure or syndrom*)) or (respiratory adj3 (disease* or disorder* or syndrom*)) or reticulocytosis or retinopathy or rheumat* or ricket* or sarcoido* or sepsis or septic* or seizure* or sclerosis or scoliosis or sickle cell or ((sjogren* or sjoegren* or sicca*) adj3 (syndrom* or disease* or disorder*)) or ((skin or connective tissue) adj3 (disease* or disorder* or syndrom*)) or sleep disorder* or sleep apnea or sleep apnoea or insomnia* or dyssomnia* or hypersomnia* or spina bifida or muscular atropy or short stature* or short bowel syndrom* or (spin* adj3 (degenerat* or disease* or disorder* or syndrom*)) or (spleen* adj3 (disease* or disorder* or syndrom*)) or spondylo* or stenosis* or stoma* or (stomach adj3 (disease* or disorder* or syndrom*)) or stroke or strokes or cerebral infarct* or tetraplegi* or thyroiditis* or (thyroid* adj3 (disease* or disorder* or dysfunction* or syndrom*)) or ((tooth or teeth or enamel) adj3 (decay* or discolo* or disease* or disorder* or dysfunction* or syndrom*)) or tropical sprue or tuberculosis or (systemic adj3 (disorder* or disease* or syndrom*)) or thrombocyto* or tremor or tremors or (turner* adj3 (syndrom* or disease* or disorder*)) or ulcer* or (urogenital adj3 (disease*

For peer review only

or disorder* or syndrom*)) or vasculopath* or (vascular adj3 (disease* or disorder* or occlu* or syndrom*)) or vestibular or ((virus or viral or bacteri* or parasit*) adj3 (infection* or disease*)) or (wasting adj3 (disorder* or disease* or syndrom*)) or (whipple adj3 (disorder* or disease* or syndrom*)) or (william* adj3 (syndrom* or disease* or disorder*)) or zoster*).ti,ab,kw. (14232139)

17 ((digestive system* or duoden* or gastr* or intestin* or nutrition* or malabsor* or metabolic*) adj3 (disease* or disorder* or syndrom* or manifestation*)).ti,ab,kw. (254303)

18 (constipation or constipated or diarrhoea or diarrhea or (abdominal adj3 (disten* or pain or bloating or cramp or cramps)) or flatulence or meteorism or steatorrhoea or steatorrhea or ((acid base or calcium or cyanocobalamin* or electrolyte* or folate or folic acid or glucose or IgA or immunoglobulin A or iron or lactose or lipid* or phosph* or protein* or triglyceride* or vitamin* or mineral*) adj3 (disorder* or deficien* or imbalance* or insufficiency or intoleran*)) or fever* or febrile or malaise or fatigue or letharg* or exhaustion or vomiting or nausea or emesis or sickness or weight loss or wait gain or hot flashes or hot flushes or medically unexplained or unexplained symptoms or unexplained medical or (signs adj2 symptoms) or sleepiness or ((calciferol or cholecalciferol or colecalciferol or egocalciferol) adj3 (disorder* or deficien* or imbalance* or insufficiency or intoleran*)) or conditions linked or linked conditions).ti,ab,kw. (1126673)

19 (medical* morbid* or (medical* adj3 comorbid*) or (medical* adj3 co-morbid*) or multimorbid* or multi* morbid* or multi* comorbid* or multi* co-morbid* or multi* physical*).ti,ab,kw. (30923)

20 first-degree relative/ (6249)

21 ((first adj5 (relative* or relation?)) and c?eliac).ti,ab,kw. (452)

22 or/13-21 (18446309)

23 (((case? adj2 find*) or detect* or diagnos* or predict* or incidence or prevalence or seroprevalence* or risk or risk factor? or risk indicator? or screen* or test*) adj3 c?eliac*).ti,ab,kw. (7675)

24 (c?eliac and ((detect* or diagnos* or predict* or incidence or prevalence or seroprevalence* or risk or risk factor? or screen* or test*) adj2 CD)).ab. (3440)

25 (((association? or associated or indicator? or indicated or predict*) adj3 (risk? or symptom* or disease? or disorder? or histor*)) and c?eliac).ti,ab,kw. (4503)

26 ((association between or relationship between) and c?eliac?).ti,ab,kw. (2279)

27 *celiac disease/di [Diagnosis] (5931)

28 celiac disease/ and (*diagnosis/ or *differential diagnosis/ or *seroprevalence/ or *screening/ or *screening test/ or *incidence/ or *prevalence/) (1064)

29 celiac disease/ and ("prediction and forecasting"/ or prediction/ or predictive validity/ or predictive value/) (755)

30 or/23-29 (16111)

31 5 and 22 and 30 (15090)

32 (((double* or dual*) adj5 diagnos* adj5 c?eliac?) or (comorbid* adj5 c?eliac)).ti,ab,kw. (85)

For peer review only

33 (high risk adj5 c?eliac?).ti,ab,kw. (103)

34 (famil* histor* adj2 c?eliac).ti,ab,kw. (93)

35 (children adj of* adj5 c?eliac).ti,ab,kw. (30)

36 celiac disease test kit/ (11)

37 or/32-36 (315)

38 12 or 31 or 37 (17603)

39 limit 38 to embase status (11243)

40 ((celiac* or coeliac*) adj2 (asymptomatic or atypical or adolesc* or adult* or cases or cohort* or child* or disease* or family or families or familial or genetic predispos* or genetic pre-dispos* or heredit* or infant* or men or patient* or people or population* or subjects or symptomatic or syndrom* or sprue or women)).ti,ab,kw. (26735)

41 limit 40 to (article-in-press status or in-process status) (372)

42 c?eliac.ti. (20558)

43 limit 38 to conference abstract status (4569)

44 42 and 43 (2794)

45 39 or 41 or 44 (14409)

46 ((celiac* or coeliac*) adj (angiograp* or arter* or axis or plexus or trunk)).ti,ab,kw,hw. (12186)

47 case report.ti. (283693)

48 ((case adj of*) and (patient or man or woman)).ti,ab. and case report.sh. (347041)

49 (editorial or letter).pt. (1747770)

50 (exp experimental organism/ or animal tissue/ or animal cell/ or exp animal disease/ or exp carnivore disease/ or exp bird/ or exp experimental animal welfare/ or exp animal husbandry/ or animal behavior/ or exp animal cell culture/ or exp marine species/ or nonhuman/ or animal.hw.) not human/ (6072159)

51 ((rat or rats or mouse or mice or rodent* or animal* or murine or porcine or feline or canine or dog or dogs or cat or cats or pig or pigs or monkey* or macaque*) not human*).ti. (1957387)

52 or/46-51 (8620748)

53 45 not 52 (12083)

54 case control study/ or hospital based case control study/ or population based case control study/ or exp longitudinal study/ or prospective study/ or retrospective study/ (1667885)

55 case finding/ or cohort analysis/ or control group/ or correlational study/ or cross-sectional study/ (1006525)

56 seroepidemiology/ (3934)

57 (cohort or case control* or case finding or cross-sectional or longitudinal).ti,ab,kw. (1661606)

For peer review only

58 study.ti. (1569542)

59 (study and (analys* or cases or clinical or control* or compar* or correlat* or associat* or epidemiolog* or evaluat* or examin* or investigat* or observ* or population based or prospectiv* or retrospectiv* or serolog*)).ti,ab,kw. (8983424)

60 (case? and control*).ab. (657067)

61 (controls or (control adj (group? or participants or patients))).ab. (1656326)

62 (groups or subgroup? or sub-group?).ab. (3028674)

63 (group? adj5 (analys* or compar* or control* or correlat* or associat*)).ab. (1489755)

64 exp regression analysis/ (434324)

65 statistical model/ (158724)

66 ((analys* or logistic*) adj1 (model* or regression*)).ab. (661228)

67 study.ab. /freq=2 (3165201)

68 or/54-67 (12212707)

69 53 and 68 (7566)

70 limit 69 to yr="1997 -Current" (7032)

****

Database: Ovid MEDLINE(R) and Epub Ahead of Print, In-Process & Other Non-Indexed Citations and Daily <1946 to February 05, 2020>

Search Strategy: ARC-Coeliac-MEDLINE-Final

----

1 Celiac Disease/ (19693)

2 c?eliac.ti,ab,kf. (27278)

3 or/1-2 (32722)

4 ((detect* or diagnos* or identif* or decision* or predict* or prognos* or risk or risks or stratif* or validat*) adj3 (model* or factor* or algorithm? or scor* or system or technique? or aid? or rule or rules or index or variable* or tool? or panel or criteri* or characteristic? or history or finding* or value* or assay or stratif* or biomarker?)).ti,ab,kf. (1978188)

5 ((detect* or diagnos* or identif* or decision* or predict* or prognos* or stratif* or validat*) adj3 (risk or risks or model* or factor* or algorithm? or scor* or system or technique? or aid? or rule or rules or index or variable* or tool? or panel or criteri* or characteristic? or history or finding* or value* or assay or stratif* or biomarker?)).ti,ab,kf. (1547617)

6 (clinic* adj3 (model* or algorithm? or scor* or risk or risks or rule or rules or predict* or index or tool? or panel or criteri* or decision* or stratif* or diagnos* or detect* or identif* or probabilit*)).ti,ab,kf. (420165)

7 ((multivaria* or multicomponent?) adj3 (model* or algorithm? or scor* or rule or rules or index or tool? or panel or criteri*)).ti,ab,kf. (69716)

8 (clinical decision making or (decision and logistic models)).sh. (7874)

9 or/4-8 (2337727)

10 3 and 9 (3401)

11 Risk Factors/ or Disease Predisposition/ or Genetic Predisposition to Disease/ (907433)

12 exp "bacterial infections and mycoses"/ or exp virus diseases/ or exp parasitic diseases/ or exp neoplasms/ or exp musculoskeletal diseases/ or exp stomatognathic diseases/ or exp respiratory tract diseases/ or exp otorhinolaryngologic diseases/ or exp nervous system diseases/ or exp eye diseases/ or exp male urogenital diseases/ or exp "female urogenital diseases and pregnancy complications"/ or exp cardiovascular diseases/ or exp "hemic and lymphatic diseases"/ or exp "congenital, hereditary, and neonatal diseases and abnormalities"/ or exp "skin and connective tissue diseases"/ or exp endocrine system diseases/ or exp immune system diseases/ or exp "disorders of environmental origin"/ or disease/ or digestive system diseases/ or exp biliary tract diseases/ or exp digestive system abnormalities/ or exp digestive system fistula/ or exp digestive system neoplasms/ or exp liver diseases/ or exp pancreatic diseases/ or exp peritoneal diseases/ or gastrointestinal diseases/ or exp esophageal diseases/ or exp gastroenteritis/ or exp gastrointestinal hemorrhage/ or exp gastrointestinal neoplasms/ or intestinal diseases/ or exp cecal diseases/ or exp colonic diseases/ or exp duodenal diseases/ or exp dysentery/ or exp enteritis/ or exp enterocolitis/ or exp hiv enteropathy/ or exp ileal diseases/ or exp inflammatory bowel diseases/ or exp intestinal atresia/ or exp intestinal diseases, parasitic/ or exp intestinal fistula/ or exp intestinal neoplasms/ or exp intestinal obstruction/ or exp intestinal perforation/ or exp intestinal polyposis/ or exp jejunal diseases/ or malabsorption syndromes/ or blind loop syndrome/ or collagenous sprue/ or lactose intolerance/ or short bowel syndrome/ or sprue, tropical/ or steatorrhea/ or whipple disease/ or mesenteric ischemia/ or mesenteric vascular occlusion/ or pneumatosis cystoides intestinalis/ or protein-losing enteropathies/ or exp rectal diseases/ or zollinger-ellison syndrome/ or exp stomach diseases/ or exp tuberculosis, gastrointestinal/ or exp visceral prolapse/ or exp liver diseases/ or exp pancreatic diseases/ or exp peritoneal diseases/ or "nutritional and metabolic diseases"/ or metabolic diseases/ or exp acid-base imbalance/ or exp bone diseases, metabolic/ or exp brain diseases, metabolic/ or exp calcium metabolism disorders/ or exp dna repair-deficiency disorders/ or exp glucose metabolism disorders/ or exp hyperlactatemia/ or exp iron metabolism disorders/ or exp lipid metabolism disorders/ or malabsorption syndromes/ or exp blind loop syndrome/ or exp collagenous sprue/ or exp hyperhomocysteinemia/ or exp lactose intolerance/ or exp sprue, tropical/ or exp steatorrhea/ or exp whipple disease/ or exp metabolic syndrome/ or exp metabolism, inborn errors/ or exp mitochondrial diseases/ or exp phosphorus metabolism disorders/ or exp porphyrias/ or exp proteostasis deficiencies/ or exp skin diseases, metabolic/ or exp wasting syndrome/ or exp water-electrolyte imbalance/ or exp nutrition disorders/ or "SIGNS and SYMPTOMS"/ or exp aging, premature/ or exp asthenia/ or exp body temperature changes/ or exp body weight/ or exp cardiac output, high/ or exp cardiac output, low/ or exp chills/ or exp cyanosis/ or exp edema/ or exp eye manifestations/ or exp failure to thrive/ or exp fatigue/ or exp feminization/ or exp fetal distress/ or exp heart murmurs/ or exp hot flashes/ or exp

For peer review only

hypergammaglobulinemia/ or exp hyperlactatemia/ or exp hypertriglyceridemic waist/ or exp intermittent claudication/ or exp medically unexplained symptoms/ or exp mobility limitation/ or exp motion sickness/ or exp myocardial stunning/ or exp neurologic manifestations/ or exp oral manifestations/ or exp polydipsia/ or exp prodromal symptoms/ or exp pseudophakia/ or exp renal colic/ or exp reticulocytosis/ or exp "signs and symptoms, digestive"/ or exp "signs and symptoms, respiratory"/ or exp skin manifestations/ or exp sleepiness/ or exp travel-related illness/ or exp urological manifestations/ or exp virilism/ (13521118)

13 exp "anatomy (non mesh)"/ab, pa [abnormalities, pathology] (1686610)

14 ((addison* adj3 (disease* or disorder* or syndrom*)) or alopecia or baldness or amenorrhea* or amenorrhoea* or oligomenorrhea* or oligomenorrhoea* or anxiety disorder* or anorexia or adrenal insufficienc* or adrenocortical insufficienc* or allerg* or anemi* or anaemi* or angina or aneurysm or ankylosing spondylitis or arthropath* or arthriti* or arthrosis or arthroses or asthma* or asthenia or ataxia or atrial fibrillation or atrophy or autoimmune disorder* or (autoimmune adj (disease* or disorder* or syndrom*)) or ataxia or avitaminosis or bonnevie ullrich or back pain or (biliary adj3 (disease* or disorder* or syndrom*)) or (bipolar adj3 disorder*) or blindness or blind loop syndrom* or (blood adj3 poisoning) or brain atroph* or ((bone or bones) adj3 (broken or disease* or disorder* or syndrom*)) or (bone* adj3 (mineral* or densit* or soft* or decay*)) or (brain adj3 (disease* or disorder* or syndrom*)) or (bronchi* adj3 (disease* or disorder* or syndrom*)) or (bowel* adj3 (disease* or disorder* or syndrom*)) or calcinosis or cancer* or carcinoma* or adenocarcinoma* or neoplasm* or neoplastic or metasta* or malignan* or tumour or tumours or tumor or tumors or (cardiac adj3 (arrest or arrhythmia* or disease* or disorder* or surg* or syndrom*)) or cardiomyopath* or ((cardiovascular or coronary) adj3 (disease* or disorder* or event* or syndrom*)) or cachexia or ((cecal or colon* or duoden*) adj3 (disease* or disorder* or syndrom*)) or cerebral palsy or (cerebro* adj3 (degenerat* or disease* or disorder* or event* or syndrom*)) or chronic obstructive disease* or cirrhosis or claudication or colic or copd or ((coordination or co-ordination) adj3 (impair* or lack)) or congenital abnormalit* or (congential adj3 (disease* or disorder* or syndrom*)) or ((connective or collagen* or skin) adj3 (disease* or disorder* or syndrom*)) or collagenous sprue or colitis or colotides or coxarthrosis or crohn* or cushing* or cyanosis or cystic fibrosis or cystitis or deaf* or deformit* or delayed puberty or dental or depressive disorder* or dysphoria or dysthymia or disabled or (physical adj3 (deform* or disab* or impair*)) or dermatitis or dermato* or dorsopath* or diabet* or down* syndrom* or duhring* or duehring* or duhrig* or duehrig* or dyscoordination or dys-coordination or dys-coordination or dys-co-ordination or dysentery or dyssynergia or dys-synergia or emaciat* or enteritis or enterocolitis* or enteropathy or dystonia or eczema or edema or oedema or elfin face sydrom* or encephalopath* or (endocrine adj3 (disease* or disorder* or syndrom*)) or enuresis or enteropath* or epilep* or (eye adj3 (disease* or disorder* or manifestation* or syndrom*)) or fatigue syndrome or chronic fatigue or failure to thrive or fibromyalgia or fibrosis or food hypersensitivity or fractures or gammaglobulinemia or gammaglobulinaemia or (gardner* adj3 (syndrom* or disease* or disorder*)) or gastritis or gastroenteritis or gout or (glomerul* adj3 (disease* or disorder* or syndrom*)) or (gonodal adj3 dysgen*) or (growth adj3 (disease* or disorder* or syndrom*)) or headache* or ((hemic or haemic or lymph*) adj3 (disease* or disorder* or syndrom*)) or hematuria or haematuria or hemophili* or haemophili* or ((hearing or visual or vision or sight) adj3 (aid* or impair* or loss)) or (heart adj3 (disease* or disorder* or syndrom* or manifestation* or myopath*)) or hemiplegi* or hepatitis or hemodialysis or haemodialysis or (heart adj3 (disease* or disorder* or failure or syndrom*)) or hiv or human immunodeficiency virus or acquired immunodeficieny syndrom* or heerfordt waldenstrom* or hidroa or hydroa or hippel lindau* or hypertensi* or hypotensi* or hyperthyroidism or hypothyroidism or hypocortisolism or hypocorticism or hypoadrenalism or

For peer review only

hyperthyroidism or hypothyroidism or incoordination or inco-ordination or in-coordination or in-co-ordination or infertility or subfertility or sub-fertility or sterility or inflammatory disease* or inflammatory bowel disease* or incontinen* or intestinal atresi* or intussusception or irritable bowel or ischemi* or ischaemi* or fistula* or (joint adj3 (disease* or disorder* or syndrom*)) or (jejunal adj3 (disease* or disorder* or syndrom*)) or kyphosis or lav-htlv* or leukemia or leukaemia or ((liver or hepatic) adj3 (disease* or disorder* or failure or syndrom*)) or lordosis or (lung adj3 (disease* or disorder*)) or lupus or lymphoma or lymphogranuloma* or lymphadenopath* or lymphotrop* or machado joseph* or macular degeneration or malnutrition or (mental adj2 (disorder* or disease* or health or illness*)) or mania or manic or (menstr* adj3 (ceas* or disturb* or disease* or disorder* or stop* or syndrom*)) or migraine* or (mitochondrial adj3 (disease* or disorder* or syndrom*)) or movement disorder* or mucinosis or musculoskeletal or narp syndrom* or necrotizing or nephrotic* or neuromuscular or non-hodgkin* or nonhodgkin* or multiple sclerosis or myeloma or myocarditis or myocardiopath* or myopath* or (myocardi* adj3 (deteri* or disease* or disorder* or syndrom* or manifestation*)) or nephrotic syndrome* or ((neurodegenerat* or neuro-degenerat) adj3 (disease* or disorder* or syndrom*)) or ((nutritional or metabolic) adj3 (disease* or disorder* or syndrom*)) or ((organ* or kidney or stem cell) adj3 (transplant* or recipient*)) or (nervous system adj3 (disease* or disorder* or syndrom*)) or (neurological adj3 (condition* or disease* or disorder* or syndrom*)) or neuropath* or neurosarcoido* or occlusion* or obesity or obese or orthopedic* or orthopaedic* or ((esophageal or oesophageal) adj3 (disease* or disorder* or syndrom*)) or ((oral or mouth) adj3 (disease* or disorder* or syndrom* or manifestation*)) or osteo* or otitis media or otorhinolaryngolog* or otosclerosis or (pancrea* adj3 (disease* or disorder* or syndrom*)) or papulosquamous or paraplegi* or parkinson* or (peripheral adj3 (arterial or vascular or disease* or disorder* or syndrom*)) or (peritoneal adj3 (disease* or disorder* or syndrom*)) or photodermato* or pick disease* or pneumo* or polio* or polyp* or polydipsia or polyarthropath* or polyarteritis or polyarthrosis or polyneuropath* or porphyrias or (pregnancy adj3 complicat*) or premature aging or proteostasis or pseudophakia or psoriasis or parapsoriasis or prolapse or (pulmonary adj3 (disease* or disorder* or syndrom*)) or purpur* or (recurren* adj3 (abortion* or miscarr*)) or (rect* adj3 (disease* or disorder* or syndrom*)) or ((renal or kidney) adj3 (disease* or disorder* or failure or syndrom*)) or (respiratory adj3 (disease* or disorder* or syndrom*)) or reticulocytosis or retinopathy or rheumat* or ricket* or sarcoido* or sepsis or septic* or seizure* or sclerosis or scoliosis or sickle cell or ((sjogren* or sjoegren* or sicca*) adj3 (syndrom* or disease* or disorder*)) or ((skin or connective tissue) adj3 (disease* or disorder* or syndrom*)) or sleep disorder* or sleep apnea or sleep apnoea or insomnia* or dyssomnia* or hypersomnia* or spina bifida or muscular atropy or short stature* or short bowel syndrom* or (spin* adj3 (degenerat* or disease* or disorder* or syndrom*)) or (spleen* adj3 (disease* or disorder* or syndrom*)) or spondylo* or stenosis* or stoma* or (stomach adj3 (disease* or disorder* or syndrom*)) or stroke or strokes or cerebral infarct* or tetraplegi* or thyroiditis* or (thyroid* adj3 (disease* or disorder* or dysfunction* or syndrom*)) or ((tooth or teeth or enamel) adj3 (decay* or discolo* or disease* or disorder* or dysfunction* or syndrom*)) or tropical sprue or tuberculosis or (systemic adj3 (disorder* or disease* or syndrom*)) or thrombocyto* or tremor or tremors or (turner* adj3 (syndrom* or disease* or disorder*)) or ulcer* or (urogenital adj3 (disease* or disorder* or syndrom*)) or vasculopath* or (vascular adj3 (disease* or disorder* or occlu* or syndrom*)) or vestibular or ((virus or viral or bacteri* or parasit*) adj3 (infection* or disease*)) or (wasting adj3 (disorder* or disease* or syndrom*)) or (whipple adj3 (disorder* or disease* or syndrom*)) or (william* adj3 (syndrom* or disease* or disorder*)) or zoster*).ti,ab,kf. (11316008)

15 ((digestive system* or duoden* or gastr* or intestin* or nutrition* or malabsor* or metabolic*) adj3 (disease* or disorder* or syndrom* or manifestation*)).ti,ab,kf. (180683)

16 (constipation or constipated or diarrhoea or diarrhea or (abdominal adj3 (disten* or pain or bloating or cramp or cramps)) or flatulence or meteorism or steatorrhoea or steatorrhea or ((acid base or calcium or cyanocobalamin* or electrolyte* or folate or folic acid or glucose or IgA or immunoglobulin A or iron or lactose or lipid* or phosph* or protein* or triglyceride* or vitamin* or mineral*) adj3 (disorder* or deficien* or imbalance* or insufficiency or intoleran*)) or fever* or febrile or malaise or fatigue or letharg* or exhaustion or vomiting or nausea or emesis or sickness or weight loss or wait gain or hot flashes or hot flushes or medically unexplained or unexplained symptoms or unexplained medical or (signs adj2 symptoms) or sleepiness or ((calciferol or cholecalciferol or colecalciferol or egocalciferol) adj3 (disorder* or deficien* or imbalance* or insufficiency or intoleran*)) or conditions linked or linked conditions).ti,ab,kf. (805001)

17 (medical* morbid* or (medical* adj3 comorbid*) or (medical* adj3 co-morbid*) or multimorbid* or multi* morbid* or multi* comorbid* or multi* co-morbid* or multi* physical*).ti,ab,kf. (18919)

18 (first-degree relative or family or family relation or family history or mother or father or parent or daughter or son).sh. or (brother or sister or sibling).hw. (83159)

19 (first adj5 (relative* or relation?)).ti,ab,kf. (21485)

20 (children adj of*).ti,ab,kf. (32315)

21 or/11-20 (16944516)

22 (((case? adj2 find*) or detect* or diagnos* or predict* or incidence or prevalence or seroprevalence* or risk or risk factor? or risk indicator? or screen* or test*) adj3 c?eliac*).ti,ab,kf. (4839)

23 (c?eliac and ((detect* or diagnos* or predict* or incidence or prevalence or seroprevalence* or risk or risk factor? or screen* or test*) adj2 CD)).ab. (1782)

24 (((association? or associated or indicator? or indicated or predict*) adj3 (risk? or symptom* or disease? or disorder? or histor*)) and c?eliac).ti,ab,kf. (2819)

25 ((association between or relationship between) and c?eliac?).ti,ab,kf. (1328)

26 celiac disease/di [Diagnosis] (5652)

27 celiac disease/ and (diagnosis/ or diagnosis, differential/ or seroprevalence/ or seroepidemiologic studies/ or screening/ or prediction/ or prevalence/ or incidence/) (2995)

28 or/22-27 (11534)

29 3 and 21 and 28 (9724)

30 Celiac Disease/di and (atypical or extraintestinal or extra-intestinal or nonspecific or non-specific).mp. (490)

31 (((double* or dual*) adj5 diagnos* adj5 c?eliac?) or (comorbid* adj5 c?eliac)).ti,ab,kf. (42)

32 (high risk adj5 c?eliac?).ti,ab,kf. (67)

33 (famil* histor* adj2 c?eliac).ti,ab,kf. (33)

34 (children adj of* adj5 c?eliac).ti,ab,kw. (19)

35 or/30-34 (642)

36 10 or 29 or 35 (11362)

37 ((celiac* or coeliac*) adj2 (asymptomatic or atypical or adolesc* or adult* or cases or cohort* or child* or disease* or family or families or familial or genetic predispos* or genetic pre-dispos* or heredit* or infant* or men or patient* or people or population* or subjects or symptomatic or syndrom* or sprue or women)).ti,ab,kf. (18800)

38 limit 37 to ("in data review" or in process or publisher) (605)

39 36 or 38 (11704)

40 ((celiac* or coeliac*) adj (angiograp* or arter* or axis or plexus or trunk)).ti,ab,kf,hw. (8461)

41 case report.ti. (230986)

42 ((case adj of*) and (patient or man or woman)).ti,ab. and case reports.pt. (252275)

43 (comment or editorial or letter or newspaper article).pt. (1825467)

44 (exp Animals/ or exp Animal Experimentation/ or exp Models, Animal/ or exp Animals, Laboratory/ or exp rodentia/) not Humans/ (4673001)

45 ((rat or rats or mouse or mice or rodent* or animal* or murine or porcine or feline or canine or dog or dogs or cat or cats or pig or pigs or monkey* or macaque*) not human*).ti. (1856683)

46 or/40-45 (7137365)

47 39 not 46 (9645)

48 exp epidemiologic studies/ (2435409)

49 (cohort or case control* or case finding or cross-sectional or longitudinal).ti,ab,kf. (1114977)

50 study.ti. (1330964)

51 (study and (analys* or cases or clinical or control* or compar* or correlat* or associat* or epidemiolog* or evaluat* or examin* or investigat* or observ* or population based or prospectiv* or retrospectiv* or serolog*)).ti,ab,kf. (6665955)

52 (case? and control*).ab. (448997)

53 (controls or (control adj (group? or participants or patients))).ab. (1189049)

54 (groups or subgroup? or sub-group?).ab. (2170205)

55 (group? adj5 (analys* or compar* or control* or correlat* or associat*)).ab. (1004351)

56 exp Regression Analysis/ (416341)

57 statistical model/ (89771)

58 ((analys* or logistic*) adj1 (model* or regression*)).ab. (456097)

59 study.ab. /freq=2 (2157950)

60 or/48-59 (9618005)

For peer review only

61 47 and 60 (5419)

62 limit 61 to yr="1997 -Current" (4786)

****

Cochrane Library, Issue 2 of 12, 2020

#1 MeSH descriptor: [Celiac Disease] this term only [63]

#2 ("celiac disease" or "coeliac disease"): kw [338]

#3 ((celiac* or coeliac*) near/3 (asymptomatic or atypical or adolesc* or adult* or cases or cohort* or child* or disease* or family or families or familial or genetic predispos* or genetic pre-dispos* or heredit* or infant* or men or patient* or people or population* or subjects or symptomatic or syndrom* or sprue or women)):ti,ab [782]

#4 (#1 or #2 or #3) [886]

#5 ((celiac* or coeliac*) next (angiograp* or arter* or axis or plexus or trunk)):ti,ab,kw [276]

#6 #4 not #5 (n=870) [870]

****

Web of Science: Science Citation Index Expanded (SCI-EXPANDED), SCI-EXPANDED, Conference Proceedings Citation Index- Science SSCI

[Limit to celiac or coeliac in title/abstract, n=6118]

#28 (#26 not #27) [7,430]

#27 (TI=("case report" or "a case of" or "a rare case of")) or (TS=(("a case of" or "a rare case of") near (patient or man or woman or child or adolescent or "an adult"))) [202,526]

#26 (#25 AND #24) [7493]
Indexes=SCI-EXPANDED, CPCI-S Timespan=1997-2020

#25 ((TS=(seroepidemiolog* or cohort or "case control*" or "case find*" or cross-sectional or longitudinal or "population based" or prospective* or retrospective*) or TI=(study) or TS=(study and (analys* or cases or clinical or control* or compar* or correlat* or associat* or epidemiolog* or evaluat* or examin* or investigat* or observ* or population based or prospectiv* or retrospectiv* or serolog*)) or TS=((case or cases) and control*) or TS=(controls or "control* group*" or "control* participant*" or "control* patient*" or groups or subgroup* or sub-group*) or TS=(group* near (analys* or compar* or control* or correlat* or associat*)) or TS=("regression analysis" or "regression model*" or "statistical model*" or "logistic* model*")) NOT (TI=(rat or rats or mouse or mice or rodent* or animal* or murine or porcine or feline or canine or dog or dogs or cat or cats or pig or pigs or monkey* or macaque* or nonhuman or "non human"))) [19,956,721
Indexes=SCI-EXPANDED, SSCI, A&HCI, CPCI-S, CPCI-SSH, ESCI Timespan=1900-2020

#24 (#23 OR #22 OR #21 OR #8) [13,272]

For peer review only

#23 (TS=((“high risk” or “famil* histor*” or “child* of”) near (celiac or coeliac)) or TS=(”celiac disease test kit”)) [233]
Indexes=SCI-EXPANDED, SSCI, A&HCI, CPCI-S, CPCI-SSH, ESCI Timespan=1900-2020

#22 (TS=((double* or dual*) near diagnos* near (celiac or coeliac))) or (TS=(comorbid* near (celiac or coeliac))) [111]
Indexes=SCI-EXPANDED, SSCI, A&HCI, CPCI-S, CPCI-SSH, ESCI Timespan=1900-2020

#21 (#20 AND #15 AND #1) [12,140]

#20 (#19 OR #18 OR #17 OR #16) [18,586]

#19 (TS=((“association* between” or “relationship between”) and (celiac or coeliac))) [1,458]
Indexes=SCI-EXPANDED, SSCI, A&HCI, CPCI-S, CPCI-SSH, ESCI Timespan=1900-2020

#18 ((TS=((association* or associated or indicator* or indicated or predict*) near (risk* or symptom* or disease* or disorder* or histor*))) and (TS=(celiac or coeliac))) [5,833]
Indexes=SCI-EXPANDED, SSCI, A&HCI, CPCI-S, CPCI-SSH, ESCI Timespan=1900-2020

#17 (TS=((celiac or coeliac) and ("detect* CD" or “CD detect*” or "detect* of CD" or "detect* with CD" or “diagnos* CD” or "CD diagnos*" or “diagnos* of CD” or "diagnos* with CD" or “predict* CD” or "CD predict*" or “predict* of CD” or "predict* with CD" or “inciden* CD” or "CD inciden*" or “inciden* of CD” or "inciden* with CD" or “prevalen* CD” or "CD prevalen*" or “prevalen* of CD” or " prevalen* with CD" or “seroprevalen* CD” or "CD seroprevalen*" or “seroprevalen* of CD” or "seroprevalen* with CD" or "CD risk*" or “risk* of CD” or “risk* for CD” or “risk* factor* of CD” or “risk factor* for CD” or “CD screen*” or "screen* for CD" or “CD test*” or "test* for CD"))) [1,489]
Indexes=SCI-EXPANDED, SSCI, A&HCI, CPCI-S, CPCI-SSH, ESCI Timespan=1900-2020

#16 (TS=(("case finding" or detect* or diagnos* or predict* or incidence or prevalence or seroprevalence* or risk or "risk factor*" or "risk indicator*" or screen* or test*) and (celiac* or coeliac*))) [17,056]
Indexes=SCI-EXPANDED, SSCI, A&HCI, CPCI-S, CPCI-SSH, ESCI Timespan=1900-2020

#15 (#14 OR #13 OR #12 OR #11 OR #10 OR #9) [14,174,134]

#14 (TI=(manifestation*)) [51,353]
Indexes=SCI-EXPANDED, SSCI, A&HCI, CPCI-S, CPCI-SSH, ESCI Timespan=1900-2020

#13 (TS=("first degree" near (related or relative* or relation*))) [11,114]
Indexes=SCI-EXPANDED, SSCI, A&HCI, CPCI-S, CPCI-SSH, ESCI Timespan=1900-2020

#12 (TS=("medical* morbid*" or (medical* near (comorbid* or co-morbid*)) or multimorbid* or "multi* morbid*" or "multi* comorbid*" or "multi* co-morbid*" or "multi* physical*")) [25,013]
Indexes=SCI-EXPANDED, SSCI, A&HCI, CPCI-S, CPCI-SSH, ESCI Timespan=1900-2020

#11 ((TS=(constipation or constipated or diarrhoea or diarrhea or (abdominal near (disten* or pain or bloating or cramp or cramps)) or flatulence or meteorism or steatorrhoea or steatorrhea)) or (TS=((“acid base” or calcium or cyanocobalamin* or electrolyte* or folate or "folic acid"or glucose or IgA or "immunoglobulin A" or iron or lactose or lipid* or phosph* or protein* or triglyceride* or vitamin* or mineral*) near (disorder* or deficien* or imbalance* or insufficiency or intoleran*))) or (TS=(fever* or febrile or malaise or fatigue or letharg* or exhaustion or vomiting or nausea or emesis or sickness or “weight loss” or “wait gain” or “hot flashes” or “hot flushes” or “medically unexplained” or “unexplained medical” or “unexplained symptom*” or “signs and symptoms” or

For peer review only

“condition* linked” or “linked condition*” or “condition* associated with” or sleepiness)) or (TS=((calciferol or cholecalciferol or colecalciferol or egocalciferol) near (disorder* or deficien* or imbalance* or insufficiency or intoleran*)))) or (TS=((anxiety or depression or mood) near (celiac or coeliac))) [1,055,350]
Indexes=SCI-EXPANDED, SSCI, A&HCI, CPCI-S, CPCI-SSH, ESCI Timespan=1900-2020

#10 (TS=("digestive system* disease*" or "digestive system* disorder*" or "digestive system* syndrom*" or "digestive system manifestation*" or "duoden* disease*" or "duoden* disorder*" or "duoden* syndrom*" or "duoden* manifestation*" or "gastr* disease*" or "gastr* disorder*" or "gastr* syndrom*" or "gastr* manifestation*" or "intestin* disease*" or "intestin* disorder*" or "intestin* syndrom*" or "intestin* manifestation*" or "malabsor* disease*" or "malabsor* disorder*" or "malabsor* syndrom*" or "malabsor* manifestation*")) [29,189]
Indexes=SCI-EXPANDED, SSCI, A&HCI, CPCI-S, CPCI-SSH, ESCI Timespan=1900-2020

#9 (TS=((addison* near (disease* or disorder* or syndrom*)) or alopecia or baldness or amenorrhea* or amenorrhoea* or oligomenorrhea* or oligomenorrhoea* or "anxiety disorder*" or anorexia or "adrenal insufficienc*" or "adrenocortical insufficienc*" or allerg* or anemi* or anaemi* or angina or aneurysm or "ankylosing spondylitis" or arthropath* or arthriti* or arthrosis or arthroses or asthma* or asthenia or ataxia or "atrial fibrillation" or atrophy or "autoimmune disorders" or "autoimmune diseases" or "autoimmune syndrom*" or ataxia or avitaminosis or "bonnevie ullrich" or "back pain" or (biliary near (disease* or disorder* or syndrom*)) or (bipolar near disorder*) or blindness or "blind loop syndrom*" or (blood near poisoning) or "brain atroph*" or ((bone or bones) near (broken or disease* or disorder* or syndrom*)) or (bone* near (mineral* or densit* or soft* or decay*)) or (brain near (disease* or disorder* or syndrom*)) or (bronchi* near (disease* or disorder* or syndrom*)) or (bowel* near (disease* or disorder* or syndrom*)) or calcinosis or cancer* or carcinoma* or adenocarcinoma* or neoplasm* or neoplastic or metasta* or malignan* or tumour or tumours or tumor or tumors or (cardiac near (arrest or arrhythmia* or disease* or disorder* or surg* or syndrom*)) or cardiomyopath* or ((cardiovascular or coronary) near (disease* or disorder* or event* or syndrom*)) or cachexia or ((cecal or colon* or duoden*) near (disease* or disorder* or syndrom*)) or "cerebral palsy" or (cerebro* near (degenerat* or disease* or disorder* or event* or syndrom*)) or "chronic obstructive disease" or cirrhosis or claudication or colic or COPD or ((coordination or co-ordination) near (impair* or lack)) or "congenital abnormalit*" or (congential near (disease or disorder* or syndrom*)) or ((connective or collagen* or skin) near (disease or disorder* or syndrom*)) or "collagenous sprue" or colitis or colotides or coxarthrosis or crohn* or cushing* or cyanosis or "cystic fibrosis" or cystitis or deaf* or deformit* or "delayed puberty" or dental or "depressive disorder*" or dysphoria or dysthymia or disabled or (physical near (deform* or disab* or impair*)) or dermatitis or dermato* or dorsopath* or diabet* or "down* syndrom*" or duhring* or duehring* or duhrig* or duehrig* or dyscoordination or dys-coordination or dys-coordination or dys-co-ordination or dysentery or dyssynergia or dys-synergia or emaciat* or enteritis or enterocolitis* or dystonia or eczema or edema or oedema or "elfin face sydrom*" or encephalopath* or (endocrine near (disease* or disorder* or syndrom*)) or enuresis or enteropath* or epilep* or (eye near (disease* or disorder* or manifestation* or syndrome*)) or "fatigue syndrome" or "chronic fatigue" or "failure to thrive" or fibromyalgia or fibrosis or "food hypersensitivity" or fractures or gammaglobulinemia or gammaglobulinaemia or (gardner* near (syndrom* or disease* or disorder*)) or gastritis or gastroenteritis or gout or (glomerul* near (disease* or disorder* or syndrom*)) or "gonodal near dysgen*" or (growth near (disease* or disorder* or syndrom*)) or headache* or ((hemic or haemic or lymph*) near (disease* or disorder* or syndrom*)) or hematuria or haematuria or hemophili* or haemophili* or ((hearing or visual or vision or sight) near (aid* or impair* or loss)) or (heart near

For peer review only

(disease* or disorder* or failure or syndrom* or manifestation* or myopathy*)) or hemiplegi* or hepatitis or hemodialysis or haemodialysis hiv or "human immunodeficiency virus" or "acquired immunodeficieny syndrom*" or "heerfordt waldenstrom*" or hidroa or hydroa or "hippel lindau*" or hypertensi* or hypotensi* or hyperthyroidism or hypothyroidism or hypocortisolism or hypocorticism or hypoadrenalism or incoordination or inco-ordination or in-coordination or in-co-ordination or infertility or subfertility or sub-fertil* or sterility or "inflammatory disease*" or "inflammatory bowel disease*" or incontinen* or "intestinal atresi*" or intussusception or "irritable bowel" or ischemi* or ischaemi* or fistula* or (joint near (disease* or disorder* or syndrom*)) or (jejunal near (disease* or disorder* or syndrom*)) or kyphosis or lav-htlv* or leukemia or leukaemia or ((liver or hepatic) near (disease* or disorder* or failure or syndrom*)) or lordosis or (lung near (disease* or disorder*)) or lupus or SLE or lymphoma or lymphogranuloma* or lymphadenopath* or lymphotrop* or "machado joseph*" or "macular degeneration" or malnutrition or (mental near (disorder* or disease* or health or illness*)) or mania or manic or (menstr* near (ceas* or disturb* or disease* or disorder* or stop* or syndrom*)) or migraine* or (mitochondrial near (disease* or disorder* or syndrom*)) or "movement disorder*" or mucinosis or musculoskeletal or "narp syndrom*" or necrotizing or nephrotic syndrom* or neuromuscular or non-hodgkin* or nonhodgkin* or "multiple sclerosis" or myeloma or myocarditis or myocardiopath* or myopath* or (myocardi* near (deteri* or disease* or disorder* or syndrom* or manifestation*)) or "nephrotic syndrome" or ((neurodegenerat* or neuro-degenerat) near (disease* or disorder* or syndrom)) or ((nutritional or metabolic) near (disease* or disorder* or syndrom*)) or ((organ* or kidney or "stem cell") near (transplant* or recipient*)) or ("nervous system" near (disease* or disorder* or syndrom*)) or (neurological near (condition* or disease* or disorder* or syndrom*)) or neuropath* or neurosarcoido* or occlusion* or obesity or obese or orthopedic* or orthopaedic* or ((esophageal or oesophageal) near (disease* or disorder* or syndrom*)) or ((oral or mouth) near (disease* or disorder* or syndrom* or manifestation*)) or osteo* or "otitis media" or otorhinolaryngolog* or otosclerosis or (pancrea* near (disease* or disorder* or syndrom*)) or papulosquamous or paraplegi* or parkinson* or (peripheral near (arterial or vascular or disease* or disorder* or syndrom*)) or (peritoneal near (disease* or disorder* or syndrom*)) or photodermato* or "pick disease*" or pneumo* or polio* or polyp* or polydipsia or polyarthropath* or polyarteritis or polyarthrosis or polyneuropath* or porphyrias or (pregnancy near complicat*) or "premature aging" or proteostasis or pseudophakia or psoriasis or parapsoriasis or prolapse or (pulmonary near (disease* or disorder* or syndrom*)) or purpur* or (recurren* near (abortion* or miscarr*)) or ((rectum or rectal) near (disease* or disorder* or syndrom*)) or ((renal or kidney) near (disease* or disorder* or failure or syndrom*)) or (respiratory near (disease* or disorder* or syndrom*)) or reticulocytosis or retinopathy or rheumat* or ricket* or sarcoido* or sepsis or septic* or seizure* or sclerosis or scoliosis or "sickle cell" or ((sjogren* or sjoegren* or sicca*) near (syndrom* or disease* or disorder*)) or ((skin or "connective tissue") near (disease* or disorder* or syndrom*)) or "sleep disorder*" or "sleep apnea" or "sleep apnoea" or insomnia* or dyssomnia* or hypersomnia* or "spina bifida" or "muscular atropy" or "short stature*" or "short bowel syndrom*" or (spin* near (degenerat* or disease* or disorder* or syndrom*)) or (spleen* near (disease* or disorder* or syndrom*)) or spondylo* or stenosis* or stoma* or (stomach near (disease* or disorder* or syndrom*)) or stroke or strokes or "cerebral infarct*" or tetraplegi* or thyroiditis* or (thyroid* near (disease* or disorder* or dysfunction* or syndrom*)) or ((tooth or teeth or enamel) near (decay* or discolo* or disease* or disorder* or dysfunction* or syndrom*)) or "tropical sprue" or tuberculosis or (systemic near (disorder* or disease* or syndrom*)) or thrombocyto* or tremor or tremors or (turner* near (syndrome* or disease* or disorder*)) or ulcer* or (urogenital near (disease* or disorder* or syndrom*)) or vasculopath* or (vascular near (disease* or disorder* or occlu* or syndrom*)) or vestibular or ((virus or viral or bacteri* or parasit*) near (infection* or disease*)) or

For peer review only

(wasting near (disorder* or disease* or syndrom*)) or (whipple near (disorder* or disease* or syndrom*)) or (william* near (syndrom* or disease* or disorder*)) or zoster*)) [13,693,359]
Indexes=SCI-EXPANDED, SSCI, A&HCI, CPCI-S, CPCI-SSH, ESCI Timespan=1900-2020

#8 (#7 and #1) [5,889]

#7 (#6 OR #5 OR #4 OR #3 OR #2) [6,827,636]

#6 (TS=("clinical decision making" or "logistic model*" or "statistical model*")) [81,189]
Indexes=SCI-EXPANDED, SSCI, A&HCI, CPCI-S, CPCI-SSH, ESCI Timespan=1900-2020

#5 (TS=((multivaria* or multicomponent*) NEAR (model* or algorithm* or score or scores or scoring or rule or rules or index or tool or tools or panel or criteri*))) [156,496]
Indexes=SCI-EXPANDED, SSCI, A&HCI, CPCI-S, CPCI-SSH, ESCI Timespan=1900-2020

#4 (TS=(clinic* NEAR (model* or algorithm* or score or scores or scoring or risk or risks or rule or rules or predict* or index or tool or tools or panel or criteri* or decision* or stratif* or diagnos* or detect* or identif* or probabilit*))) [1,061,406]
Indexes=SCI-EXPANDED, SSCI, A&HCI, CPCI-S, CPCI-SSH, ESCI Timespan=1900-2020

#3 (TS=((detect* or diagnos* or identif* or decision* or predict* or prognos* or stratif* or validat*) NEAR (risk or risks or model* or factor* or algorithm* or score or scores or scoring or system or technique* or aid or aids or rule or rules or index or variable* or tool or tools or panel or criteri* or characteristic* or history or finding* or value* or assay or stratif* or biomarker*))) [5,481,423]
Indexes=SCI-EXPANDED, SSCI, A&HCI, CPCI-S, CPCI-SSH, ESCI Timespan=1900-2020

#2 (TS=((detect* or diagnos* or identif* or decision* or predict* or prognos* or risk or risks or stratif* or validat*) NEAR (model* or factor* or algorithm* or score or scores or scoring or system or technique* or aid or aids or rule or rules or index or variable* or tool or tools or panel or criteri* or characteristic* or history or finding* or value* or assay or stratif* or biomarker*))) [6/133,858]
Indexes=SCI-EXPANDED, SSCI, A&HCI, CPCI-S, CPCI-SSH, ESCI Timespan=1900-2020

#1 TS=((celiac* OR coeliac*) NEAR (asymptomatic OR atypical OR adolesc* OR adult* OR cases OR cohort* OR child* OR disease* OR disorder* OR family OR families OR familial OR "genetic predispos*" OR "genetic pre-dispos*" OR heredit* OR infant* OR men OR patient* OR people OR population* OR subjects OR symptomatic OR syndrom* OR sprue OR women))) NOT (TS=((celiac* OR coeliac*) NEAR (angiography OR artery OR arteries OR axis OR plexus OR trunk))) [25,557]
Indexes=SCI-EXPANDED, SSCI, A&HCI, CPCI-S, CPCI-SSH, ESCI Timespan=1900-2020

For peer review only

Reporting checklist for protocol of a systematic review.

Based on the PRISMA-P guidelines.

Instructions to authors

Complete this checklist by entering the page numbers from your manuscript where readers will find each of the items listed below.

Your article may not currently address all the items on the checklist. Please modify your text to include the missing information. If you are certain that an item does not apply, please write "n/a" and provide a short explanation.

Upload your completed checklist as an extra file when you submit to a journal.

In your methods section, say that you used the PRISMA-Preporting guidelines, and cite them as:

Moher D, Shamseer L, Clarke M, Ghersi D, Liberati A, Petticrew M, Shekelle P, Stewart LA. Preferred Reporting Items for Systematic Review and Meta-Analysis Protocols (PRISMA-P) 2015 statement. Syst Rev. 2015;4(1):1.

		Reporting Item	Page Number
**Title**			
Identification	#1a	Identify the report as a protocol of a systematic review	1

For peer review only

Update	#1b	If the protocol is for an update of a previous systematic review, identify as such	NA
**Registration**			
	#2	If registered, provide the name of the registry (such as PROSPERO) and registration number	1
**Authors**			
Contact	#3a	Provide name, institutional affiliation, e-mail address of all protocol authors; provide physical mailing address of corresponding author	1
Contribution	#3b	Describe contributions of protocol authors and identify the guarantor of the review	11
**Amendments**			
	#4	If the protocol represents an amendment of a previously completed or published protocol, identify as such and list changes; otherwise, state plan for documenting important protocol amendments	6
**Support**			
Sources	#5a	Indicate sources of financial or other support for the review	11
Sponsor	#5b	Provide name for the review funder and / or sponsor	11

For peer review only

Role of sponsor or funder	#5c	Describe roles of funder(s), sponsor(s), and / or institution(s), if any, in developing the protocol	NA
**Introduction**			
Rationale	#6	Describe the rationale for the review in the context of what is already known	4-5
Objectives	#7	Provide an explicit statement of the question(s) the review will address with reference to participants, interventions, comparators, and outcomes (PICO)	5
**Methods**			
Eligibility criteria	#8	Specify the study characteristics (such as PICO, study design, setting, time frame) and report characteristics (such as years considered, language, publication status) to be used as criteria for eligibility for the review	6
Information sources	#9	Describe all intended information sources (such as electronic databases, contact with study authors, trial registers or other grey literature sources) with planned dates of coverage	6
Search strategy	#10	Present draft of search strategy to be used for at least one electronic database, including planned limits, such that it could be repeated	7 and Supplementary material

For peer review only

Study records - data management	#11a	Describe the mechanism(s) that will be used to manage records and data throughout the review	7
Study records - selection process	#11b	State the process that will be used for selecting studies (such as two independent reviewers) through each phase of the review (that is, screening, eligibility and inclusion in meta-analysis)	7
Study records - data collection process	#11c	Describe planned method of extracting data from reports (such as piloting forms, done independently, in duplicate), any processes for obtaining and confirming data from investigators	7
Data items	#12	List and define all variables for which data will be sought (such as PICO items, funding sources), any pre-planned data assumptions and simplifications	7
Outcomes and prioritization	#13	List and define all outcomes for which data will be sought, including prioritization of main and additional outcomes, with rationale	7
Risk of bias in individual studies	#14	Describe anticipated methods for assessing risk of bias of individual studies, including whether this will be done at the outcome or study level, or both; state how this information will be used in data synthesis	8
Data synthesis	#15a	Describe criteria under which study data will be quantitatively synthesised	8-9

For peer review only

Data synthesis	#15b	If data are appropriate for quantitative synthesis, describe planned summary measures, methods of handling data and methods of combining data from studies, including any planned exploration of consistency (such as I2, Kendall's τ)	8-9
Data synthesis	#15c	Describe any proposed additional analyses (such as sensitivity or subgroup analyses, meta-regression)	8-9
Data synthesis	#15d	If quantitative synthesis is not appropriate, describe the type of summary planned	8-9
Meta-bias(es)	#16	Specify any planned assessment of meta-bias(es) (such as publication bias across studies, selective reporting within studies)	8-9
Confidence in cumulative evidence	#17	Describe how the strength of the body of evidence will be assessed (such as GRADE)	NA

Notes:

- 10: 7 and Supplementary material The PRISMA-P checklist is distributed under the terms of the Creative Commons Attribution License CC-BY 4.0. This checklist was completed on 30. March 2020 using https://www.goodreports.org/, a tool made by the EQUATOR Network in collaboration with Penelope.ai

For peer review only
